# Identification of candidate biomarkers and therapeutic agents for heart failure by bioinformatics analysis

**DOI:** 10.1186/s12872-021-02146-8

**Published:** 2021-07-04

**Authors:** Vijayakrishna Kolur, Basavaraj Vastrad, Chanabasayya Vastrad, Shivakumar Kotturshetti, Anandkumar Tengli

**Affiliations:** 1 Vihaan Heart Care & Super Specialty Centre, Vivekananda General Hospital, Deshpande Nagar, Hubli, Karnataka 580029 India; 2 Department of Biochemistry, Basaveshwar College of Pharmacy, Gadag, Karnataka 582103 India; 3 Biostatistics and Bioinformatics, Chanabasava Nilaya, Bharthinagar, Dharwad, 580001 Karnataka India; 4 Department of Pharmaceutical Chemistry, JSS College of Pharmacy, Mysuru and JSS Academy of Higher Education & Research, Mysuru, Karnataka 570015 India

**Keywords:** Heart failure, Differentially expressed genes, Molecular docking, Enrichment analysis, Prognosis

## Abstract

**Introduction:**

Heart failure (HF) is a heterogeneous clinical syndrome and affects millions of people all over the world. HF occurs when the cardiac overload and injury, which is a worldwide complaint. The aim of this study was to screen and verify hub genes involved in developmental HF as well as to explore active drug molecules.

**Methods:**

The expression profiling by high throughput sequencing of GSE141910 dataset was downloaded from the Gene Expression Omnibus (GEO) database, which contained 366 samples, including 200 heart failure samples and 166 non heart failure samples. The raw data was integrated to find differentially expressed genes (DEGs) and were further analyzed with bioinformatics analysis. Gene ontology (GO) and REACTOME enrichment analyses were performed via ToppGene; protein–protein interaction (PPI) networks of the DEGs was constructed based on data from the HiPPIE interactome database; modules analysis was performed; target gene—miRNA regulatory network and target gene—TF regulatory network were constructed and analyzed; hub genes were validated; molecular docking studies was performed.

**Results:**

A total of 881 DEGs, including 442 up regulated genes and 439 down regulated genes were observed. Most of the DEGs were significantly enriched in biological adhesion, extracellular matrix, signaling receptor binding, secretion, intrinsic component of plasma membrane, signaling receptor activity, extracellular matrix organization and neutrophil degranulation. The top hub genes ESR1, PYHIN1, PPP2R2B, LCK, TP63, PCLAF, CFTR, TK1, ECT2 and FKBP5 were identified from the PPI network. Module analysis revealed that HF was associated with adaptive immune system and neutrophil degranulation. The target genes, miRNAs and TFs were identified from the target gene—miRNA regulatory network and target gene—TF regulatory network. Furthermore, receiver operating characteristic (ROC) curve analysis and RT-PCR analysis revealed that ESR1, PYHIN1, PPP2R2B, LCK, TP63, PCLAF, CFTR, TK1, ECT2 and FKBP5 might serve as prognostic, diagnostic biomarkers and therapeutic target for HF. The predicted targets of these active molecules were then confirmed.

**Conclusion:**

The current investigation identified a series of key genes and pathways that might be involved in the progression of HF, providing a new understanding of the underlying molecular mechanisms of HF.

**Supplementary Information:**

The online version contains supplementary material available at 10.1186/s12872-021-02146-8.

## Introduction

Heart failure (HF) is a cardiovascular disease characterized by tachycardia, tachypnoea, pulmonary rales, pleural effusion, raised jugular venous pressure, peripheral oedema and hepatomegaly [1]. Morbidity and mortality linked with HF is a prevalent worldwide health problem holding a universal position as the leading cause of death [2]. The numbers of cases of HF are rising globally and it has become a key health issue. According to a survey, the prevalence HF is expected to exceed 50% of the global population [3]. Research suggests that modification in multiple genes and signaling pathways are associated in controlling the advancement of HF. However, a lack of investigation on the precise molecular mechanisms of HF development limits the treatment efficacy of the disease at present.

Previous study showed that HF was related to the expression of MECP2 [4] RBM20 [5], CaMKII [6], troponin I [7] and SERCA2a [8]. Toll-Like receptor signaling pathway [9], activin type II receptor signaling pathway [10], CaMKII signaling pathways [11], Drp1 signaling pathways [12] and JAK-STAT signaling pathway [13] were liable for progression of HF. More investigations are required to focus on treatments that enhance the outcome of patients with HF, to strictly make the diagnosis of the disease based on screening of biomarkers. These investigations can upgrade prognosis of patients by lowering the risk of advancement of HF and related complications. So it is essential to recognize the mechanism and find biomarkers with a good specificity and sensitivity.

The recent high-throughput RNA sequencing data has been widely employed to screen the differentially expressed genes (DEGs) between normal samples and HF samples in human beings, which makes it accessible for us to further explore the entire molecular alterations in HF at multiple levels involving DNA, RNA, proteins, epigenetic alterations, and metabolism [14]. However, there still exist obstacles to put these RNA seq data in application in clinic for the reason that the number of DEGs found by expression profiling by high throughput sequencing were massive and the statistical analyses were also too sophisticated [15–19]

In this study, first, we had chosen dataset GSE141910 from Gene Expression Omnibus (GEO) (http:// www.ncbi.nlm.nih.gov/geo/) [20]. Second, we applied for limma tool in R software to obtain the differentially expressed genes (DEGs) in this dataset. Third, the ToppGene was used to analyze these DEGs including biological process (BP), cellular component (CC) and molecular function (MF) REACTOME pathways. Fourth, we established protein–protein interaction (PPI) network and then applied Cytotype PEWCC1 for module analysis of the DEGs which would identify some hub genes. Fifth, we established target gene—miRNA regulatory network and target gene—TF regulatory network. In addition, we further validated the hub genes by receiver operating characteristic (ROC) curve analysis and RT-PCR analysis. Finally, we performed molecular docking studies for over expressed hub genes. Results from the present investigation might provide new vision into potential prognostic and therapeutic targets for HF.

## Materials and methods

### Data resource

Expression profiling by high throughput sequencing with series number GSE141910 based on platform GPL16791 was downloaded from the GEO database. The dataset of GSE141910 contained 200 heart failure samples and 166 non heart failure samples. It was downloaded from the GEO database in NCBI based on the platform of GPL16791 Illumina HiSeq 2500 (Homo sapiens).

### Identification of DEGs in HF

DEGs of dataset GSE141910 between HF groups and non heart failure groups were respectively analyzed using the limma package in R [21]. Fold changes (FCs) in the expression of individual genes were calculated and DEGs with *P* < 0.05, |log FC|> 1.158 for up regulated genes and |log FC|< − 0.83 for down regulated genes were considered to be significant. Hierarchical clustering and visualization were used by Heat-map package of R.

### Functional enrichment analysis

Gene Ontology (GO) analysis and REACTOME pathway analysis were performed to determine the functions of DEGs using the ToppGene (ToppFun) (https://toppgene.cchmc.org/enrichment.jsp) [22] GO terms (http://geneontology.org/) [23] included biological processes (BP), cellular components (CC) and molecular functions (MF) of genomic products. REACTOME (https://reactome.org/) [24] analyzes pathways of important gene products. ToppGene is a bioinformatics database for analyzing the functional interpretation of lists of proteins and genes. The cutoff value was set to *P* < 0.05.

### Protein–protein interaction network construction and module screening

PPI networks are used to establish all protein coding genes into a massive biological network that serves an advance compassionate of the functional system of the proteome [25]. The HiPPIE interactome (https://cbdm.uni-mainz.de/hippie/) [26] database furnish information regarding predicted and experimental interactions of proteins. In the current investigation, the DEGs were mapped into the HiPPIE interactome database to find significant protein pairs with a combined score of > 0.4. The PPI network was subsequently constructed using Cytoscape software, version 3.8.2 (www.cytoscape.org) [27]. The nodes with a higher node degree [28], higher betweenness centrality [29], higher stress centrality [30] and higher closeness centrality [31] were considered as hub genes. Additionally, cluster analysis for identifying significant function modules with a degree cutoff > 2 in the PPI network was performed using the PEWCC1 (http://apps.cytoscape.org/apps/PEWCC1) [32] in Cytoscape.

### Target gene—miRNA regulatory network construction

The miRNet database (https://www.mirnet.ca/) [33] contains information on miRNA and the regulated genes. Using information collected from the miRNet database, hub genes were matched with their associated miRNA. The target gene—miRNA regulatory network then was constructed using Cytoscape software. MiRNAs and target are selected based on highest node degree.

### Target gene—TF regulatory network construction

The NetworkAnalyst database (https://www.networkanalyst.ca/) [34] contains information on TF and the regulated genes. Using information collected from the NetworkAnalyst database, hub genes were matched with their associated TF. The target gene—TF regulatory network then was constructed using Cytoscape software. TFs and target genes are selected based on highest node degree.

### Receiver operating characteristic (ROC) curve analysis

Then ROC curve analysis was implementing to classify the sensitivity and specificity of the hub genes for HF diagnosis and we investigated how large the area under the curve (AUC) was by using the statistical package pROC in R software [35].

### RT-PCR analysis

H9C2 cells (ATCC) were cultured in Dulbecco’s minimal essential medium (DMEM) (Sigma-Aldrich) supplemented with 10% fetal calf serum (Sigma-Aldrich) and 1% streptomycin (Sigma-Aldrich) at 37 °C in 5% CO_2_. HL-1 cells (ATCC) was culture in Claycomb medium (Sigma-Aldrich) supplemented with 10% fetal bovine serum (Sigma-Aldrich), 1% streptomycin (Sigma-Aldrich), 1% glutamax (Sigma-Aldrich) and 0.1 mM norepinephrine (Sigma-Aldrich) at 37 °C in 5% CO_2_. Total RNA was isolated from cell culture of H9C2 for HF and HL-1 for normal control using the TRI Reagent (Sigma, USA). cDNA was synthesized using 2.0 μg of total RNA with the Reverse transcription cDNA kit (Thermo Fisher Scientific, Waltham, MA, USA). The 7 Flex real-time PCR system (Thermo Fisher Scientific, Waltham, MA, USA) was employed to detect the relative mRNA expression. The relative expression levels were determined by the 2-ΔΔCt method and normalized to internal control beta-actin [36]. All RT-PCR reactions were performed in triplicate. The primers used to explore mRNA expression of ten hub genes were listed in Table 1.

**Table 1:** The sequences of primers for quantitative RT-PCR

Genes	Forward primers	Reverse primers
ESR1	CCTCTGGCTACCATTATGGG	AGTCATTGTGTCCTTGAATGC
PYHIN1	GCAAGATCAGTACGACAGAG	AGATAACTGAGCAACCTGTG
PPP2R2B	ACCAGAGACTATCTGACCG	GTAGTCATGAACCTGGTATGTC
LCK	CTAGTCCGGCTTTATGCAG	AAATCTACTAGGCTCCCGT
TP63	ATTCAATGAGGGACAGATTGC	GGGTCTTCTACATACTGGGC
PCLAF	GACCAATATAAACTGTGGCGGG	CCAGGGTAAACAAGGAGACGTT
CFTR	CTGTGGCCTTGGTTTACTG	CTCTGATCTCTGTACTTCACCA
TK1	AGATTCAGGTGATTCTCGGG	ACTTGTACTGGGCGATCTG
ECT2	GCTGTATTGTACGAGTATGCT	GTCACCAATTTGACAAGCTC
FKBP5	CCTAAGTTTGGCATTGACCC	CCAAGATTCTTTGGCCTTCTC

### Identification of candidate small molecules

SYBYL-X 2.0 perpetual drug design software has been used for surflex-docking studies of the designed novel molecules and the standard on over expressed genes of PDB protein. Using ChemDraw Software, all designed molecules and standards were sketched, imported and saved using open babel free software in sdf. template. The protein of over expressed genes of ESR1, LCK, PPP2R2B, TP63 and their co-crystallised protein of PDB code 4PXM, 1KSW, 2HV7, 3VD8 and 6RU6 were extracted from Protein Data Bank [37–40]. Optimizations of the designed molecules were performed by standard process by applying Gasteiger Huckel (GH) charges together with the TRIPOS force field. In addition, energy minimization was achieved using MMFF94s and MMFF94 algorithm methods. The preparation of the protein was done after protein incorporation. The co-crystallized ligand and all water molecules have been eliminated from the crystal structure; more hydrogen’s were added and the side chain was set, TRIPOS force field was used for the minimization of structure. The interaction efficiency of the compounds with the receptor was expressed in kcal/mol units by the Surflex-Dock score. The best location was integrated into the molecular region by the interaction between the protein and the ligand. Using Discovery Studio Visualizer, the visualisation of ligand interaction with receptor is performed.

## Results

### Identification of DEGs in HF

**Fig. 1:**
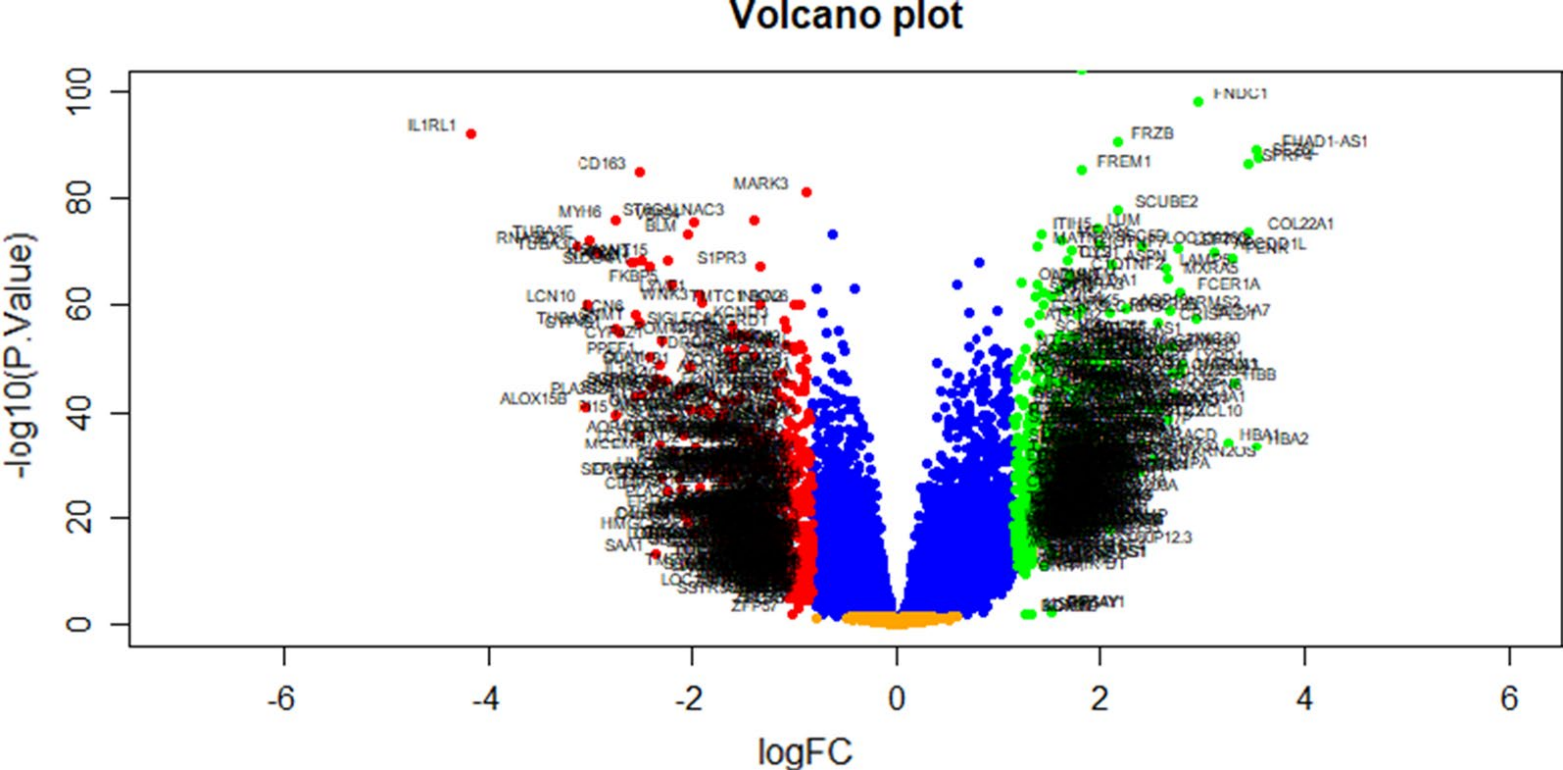
Volcano plot of differentially expressed genes. Genes with a significant change of more than two-fold were selected. Green dot represented up regulated significant genes and red dot represented down regulated significant genes

**Fig. 2:**
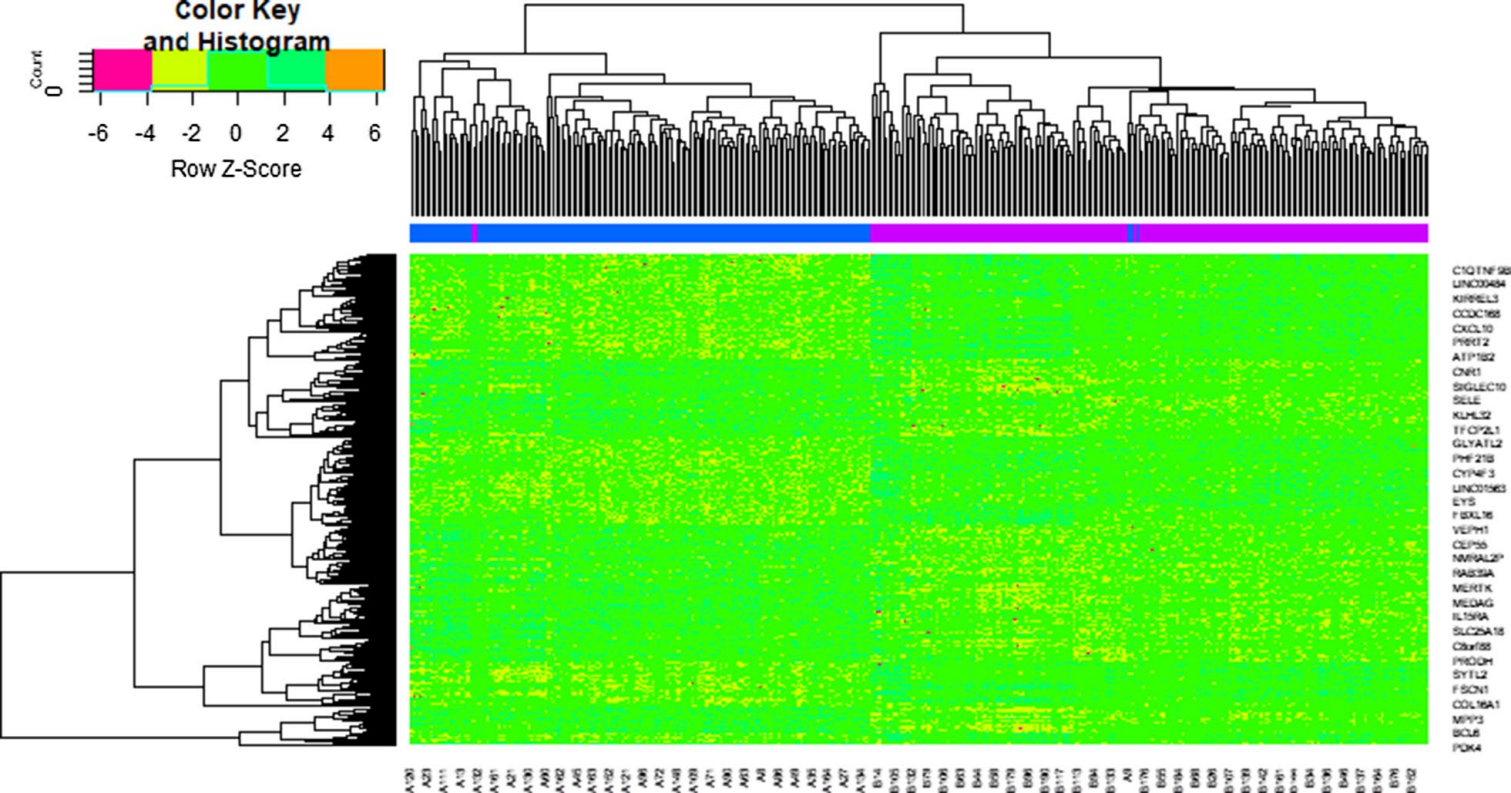
Heat map of differentially expressed genes. Legend on the top left indicate log fold change of genes. (A1 – A200 = heart failure samples; B1 – B166 = non heart failure samples)

We identified 881 DEGs in the GSE141910 dataset using the limma package in R. Based on the limma analysis, using the adj *P* val < 0.05, |log FC|> 1.158 for up regulated genes and |log FC|< − 0.83 for down regulated genes, a total of 881 DEGs were identified, consisting of 442 genes were up regulated and 439 genes were down regulated. The DEGs are listed in Additional file 1: Table S1. The volcano plot for DEGs is illustrated in Fig. 1. Figure 2 is the hierarchical clustering heat-map.

### Functional enrichment analysis

Results of GO analysis showed that the up regulated genes were significantly enriched in BP, CC, and MF, including biological adhesion, regulation of immune system process, extracellular matrix, cell surface, signaling receptor binding and molecular function regulator (Table 2); the down regulated genes were significantly enriched in BP, CC, and MF, including secretion, defense response, intrinsic component of plasma membrane, whole membrane, signaling receptor activity and molecular transducer activity (Table 2). Pathway analysis showed that the up regulated genes were significantly enriched in extracellular matrix organization and immunoregulatory interactions between a lymphoid and a non-lymphoid cell (Table 3); the down regulated genes were significantly enriched in neutrophil degranulation and SLC-mediated transmembrane transport (Table 3).

**Table 2:** The enriched GO terms of the up and down regulated differentially expressed genes

GO ID	CATEGORY	GO Name	P Value	FDR B&H	FDR B&Y	Bonferroni	Gene Count	Gene
*Up regulated genes*								
GO:0022610	BP	biological adhesion	1.32E−13	3.37E−10	3.08E−09	6.75E−10	72	HLA-DQA1, DACT2, CD83, MDK, UBASH3A, ITGBL1, FAP, MFAP4, SERPINE2, NRXN2, COL14A1, CCR7, ALOX15, COL1A1, LAMB4, COL8A2, STAB2, COL16A1, COMP, TBX21, FERMT1, XG, CCDC80, APOA1, PODXL2, ZAP70, HAPLN1, TENM4, SKAP1, CNTNAP2, PDE5A, CARD11, CTNNA2, SLAMF7, ATP1B2, CX3CR1, LRRC15, IDO1, MYOC, SIGLEC8, ISLR, SMOC2, ITGAL, ITGB7, FREM1, PTN, KIRREL3, NTM, GLI2, FBLN7, DPT, NT5E, ECM2, LCK, OMG, OPCML, TGFB2, RASGRP1, CD2, CD3E, THBS4, CD5, CD6, THY1, TIGIT, CD27, CD40LG, ROBO2, GREM1, LY9, HBB, LEF1
GO:0002682	BP	regulation of immune system process	2.20E−10	2.25E−07	2.05E−06	1.12E−06	72	IL34, HLA-DQA1, ESR1, TLR7, CD83, IL17D, MDK, UBASH3A, TNFRSF4, PYHIN1, ZBP1, FCER1A, MS4A2, FCER2, FCN1, CCR7, SMPD3, CCL24, SCARA3, ALOX15, COL1A1, IL31RA, TBX21, XG, CXCL14, APOA1, ZAP70, SH2D1B, SKAP1, PDE5A, CARD11, SLAMF7, CTSG, CX3CR1, IDO1, CXCL10, ITGAL, ACE, SIT1, ITGB7, PTN, TBC1D10C, FCRL3, BPI, GLI2, KLRB1, NPPA, CAMK4, LCK, TGFB2, RASGRP1, CD1C, CD1E, CD2, CD3D, CD3E, THBS4, CD3G, CD247, CD5, CD6, THY1, TIGIT, MS4A1, CD27, GPR68, CD40LG, CD48, GREM1, SH2D1A, LEF1, LRRC17
GO:0031012	CC	extracellular matrix	1.09E−20	2.77E−18	1.89E−17	5.54E−18	52	MATN2, COL22A1, MDK, COLQ, MFAP4, SERPINE2, HMCN2, AEBP1, FCN1, CMA1, CTHRC1, COL14A1, SCARA3, COL1A1, LAMB4, COL8A2, COL9A1, COL9A2, COL10A1, MXRA5, FMOD, COL16A1, COMP, CCDC80, APOA1, HAPLN1, CTSG, ADAMTSL2, LRRC15, ASPN, MYOC, NDP, SMOC2, FREM1, PTN, SSC5D, SULF1, DPT, NPPA, ADAMTSL1, ECM2, OGN, ITIH5, TGFB2, LEFTY2, EYS, THBS4, P3H2, LTBP2, GREM1, LUM, LRRC17
GO:0009986	CC	cell surface	5.07E−17	8.61E−15	5.86E−14	2.58E−14	63	HYAL4, NRG1, HLA-DQA1, CD83, TNFRSF4, ITGBL1, FAP, SERPINE2, FCER1A, MS4A2, FCER2, FCN1, CXCL9, CCR7, IL31RA, SFRP4, STAB2, DUOX2, APOA1, ACKR4, FCRL6, SCUBE2, CNTNAP2, SLAMF7, CTSG, IL2RB, CX3CR1, LRRC15, CXCL10, NDP, ITGAL, ACE, ITGB7, GFRA3, PTN, PROM1, SSC5D, FCRL3, SULF1, MRC2, NTM, CLEC9A, NT5E, TGFB2, LHCGR, CD1C, HHIP, CD1E, CD2, CD3D, CD3E, CD3G, CD5, CD6, THY1, TIGIT, MS4A1, CD27, CD40LG, CD48, ROBO2, GREM1, LY9
GO:0005102	MF	signaling receptor binding	1.36E−09	5.99E−07	4.41E−06	1.20E−06	73	IL34, NRG1, HLA-DQA1, ESR1, GDF6, PENK, TAC4, KDM5D, IL17D, MDK, ITGBL1, FAP, SERPINE2, FCER2, NRXN2, FCN1, CLEC11A, UCHL1, AGTR2, CXCL9, NGEF, CTHRC1, C1QTNF2, CCL22, CCL24, CXCL11, COL16A1, COMP, WNT10B, WNT9A, CXCL14, APOA1, FCRL6, GNA14, OASL, RASL11B, LRRC15, CXCL10, ADAM18, MYOC, SYTL2, NDP, ACE, GDNF, ITGB7, GFRA3, PTN, LYPD1, SCG2, NPPA, NPPB, MCHR1, ECM2, CMTM2, ESM1, LCK, OGN, TGFB2, LEFTY2, CD2, CD3E, THBS4, CD3G, THY1, TIGIT, MS4A1, C1QTNF9, CD40LG, LTB, GREM1, SYTL1, LEF1, LGI1
GO:0098772	MF	molecular function regulator	3.79E−04	1.59E−02	1.17E−01	3.34E−01	58	IL34, NRG1, ESR1, GDF6, PENK, TAC4, IL17D, MDK, KCNIP1, SERPINE2, MYOZ1, NRXN2, CLEC11A, SCN2B, AGTR2, CXCL9, NGEF, CCL22, CCL24, CXCL11, HTR2B, PI16, SCG5, WNT10B, WNT9A, CXCL14, APOA1, LRRC55, PPP2R2B, ATP1B2, CXCL10, NDP, BIRC7, GDNF, PTN, TBC1D10C, LYPD1, SCG2, NPPA, NPPB, AZIN2, CMTM2, OGN, RGS4, ITIH5, TGFB2, LEFTY2, RASGRP1, THBS4, THY1, CD27, C1QTNF9, CD40LG, RGS17, LTB, GREM1, LEF1, INKA1
*Down regulated genes*								
GO:0046903	BP	secretion	1.07E−11	5.64E−08	5.16E−07	5.64E−08	78	SERPINA3, HK3, SYN3, ACP3, TRPC4, CFTR, CD109, HMOX2, CHI3L1, F5, F8, F13A1, S100A8, S100A9, SAA1, FCER1G, MGST1, PIK3C2A, HP, AGTR1, PLA2G2A, CCR1, FGF10, C1QTNF1, PLA2G4F, FGR, MERTK, SERPINF2, ALOX5, SYT13, IL17RB, CNR1, ALOX15B, FLT3, ANPEP, P2RY12, ANXA3, FPR1, CR1, SLC1A1, SLC2A1, ARG1, ARNTL, SLC11A1, SLC22A16, LGI3, NSG1, ATP2A2, IL10, SIGLEC9, GPR84, NHLRC2, SSTR5, HPSE, KCNB1, IL1R2, PTX3, GLUL, SYN2, BANK1, WNK3, KNG1, CRISPLD2, CACNA1E, CD177, SIGLEC14, EDN1, EDN2, EDNRB, THBS1, RNASE2, CD38, TLR2, SERPINE1, ELANE, STEAP3, IL1RL1, MCEMP1
GO:0006952	BP	defense response	1.04E−06	1.63E−04	1.49E−03	5.50E−03	65	SERPINA3, EREG, VSIG4, TMIGD3, CLEC7A, RAET1E, CHI3L1, F8, CD163, S100A8, S100A9, SAA1, FCER1G, HP, HPR, AGTR1, PLA2G2A, CCR1, FGR, SERPINF2, ALOX5, ALOX5AP, IL17RB, CNR1, SELE, ADAMTS4, ANXA3, FPR1, APOB, SAMHD1, CR1, FCN3, AQP4, ARG1, SLC11A1, MARCO, IL10, BCL6, IL18R1, GGT5, IL1R2, PTX3, SIGLEC10, KNG1, CACNA1E, CD177, SOCS3, SIGLEC14, ADAMTS5, LBP, S1PR3, EDN1, EDNRB, FOSL1, THBS1, RNASE2, NAMPT, TLR2, SERPINE1, ELANE, IRAK3, ELF3, IL1RL1, CALCRL, OSMR
GO:0031226	CC	intrinsic component of plasma membrane	1.74E−10	4.55E−08	3.11E−07	9.10E−08	74	TPO, EREG, OPN4, TRPC4, CFTR, TMIGD3, KCNIP2, CD163, FCER1G, SCN3A, AGTR1, CCR1, C1QTNF1, MERTK, SYT13, IL17RB, CNR1, TRHDE, SELE, LRRC8E, FLT3, SLC4A7, P2RY12, SLC31A2, CR1, LGR5, AQP3, AQP4, SLC1A1, SLC2A1, SLC5A1, MSR1, SLC11A1, SIGLEC7, ART3, SLCO2A1, ATP2A2, MARCO, GABRR2, SIGLEC9, SLCO4A1, GPR84, SSTR2, SSTR5, IL18R1, LAPTM5, GGT5, SLC52A3, LYVE1, KCNA7, KCNB1, KCND3, NECTIN1, KCNK1, KCNK3, KCNS2, ADGRD1, CACNA1E, GPR4, GPR12, SLC38A4, GPR183, GPRC5A, RGR, S1PR3, RHAG, EDNRB, TGFBR3, TLR2, LGR6, CALCRL, OSMR, HAS2, CDH16
GO:0098805	CC	whole membrane	1.91E−03	4.99E−02	3.41E−01	9.97E−01	51	EREG, SYN3, ACP3, TRPC4, CFTR, CD109, HMOX2, CD163, FCER1G, MGST1, PLA2G4F, GPAT2, MOG, CNR1, SELE, ANPEP, P2RY12, ANXA3, FPR1, APOB, SCGN, CR1, AQP4, SLC1A1, SLC2A1, ARG1, MSR1, SLC11A1, NSG1, RAB39A, MARCO, SIGLEC9, GPR84, HPSE, LAPTM5, KCND3, SYN2, SLC9A7, WASF1, CD177, SIGLEC14, GRB14, STEAP4, EDNRB, GRIP1, CD38, TLR2, STEAP3, HAS2, SERPINA5, MCEMP1
GO:0038023	MF	signaling receptor activity	2.36E−04	1.97E−02	1.49E−01	2.53E−01	55	EREG, OPN4, CLEC7A, FCER1G, FCGR3A, AGTR1, CCR1, ADGRF5, ADGRF4, MERTK, IL17RB, CNR1, SELE, FLT3, MYOT, ANPEP, P2RY12, ANXA3, FPR1, CR1, LGR5, SIGLEC7, MARCO, PALLD, IL10, GABRR2, IL15RA, GPR82, DNER, PAQR5, GPR84, IL20RA, SSTR2, SSTR5, IL18R1, LYVE1, IL1R2, NECTIN1, ADGRD1, GPR4, GPR12, NPTX2, GPR183, GPRC5A, PKHD1L1, RGR, S1PR3, EDNRB, TGFBR3, TLR2, SERPINE1, IL1RL1, LGR6, CALCRL, OSMR
GO:0060089	MF	molecular transducer activity	4.71E−04	2.10E−02	1.59E−01	5.04E−01	58	EREG, OPN4, CLEC7A, FCER1G, FCGR3A, AGTR1, CCR1, ADGRF5, ADGRF4, MERTK, IL17RB, CNR1, SELE, FLT3, MYOT, ANPEP, P2RY12, ANXA3, FPR1, CR1, LGR5, SIGLEC7, MARCO, PALLD, IL10, GABRR2, IL15RA, GPR82, DNER, PAQR5, GPR84, IL20RA, STOX1, SSTR2, SSTR5, IL18R1, BLM, CDKL5, LYVE1, IL1R2, NECTIN1, ADGRD1, GPR4, GPR12, NPTX2, GPR183, GPRC5A, PKHD1L1, RGR, S1PR3, EDNRB, TGFBR3, TLR2, SERPINE1, IL1RL1, LGR6, CALCRL, OSMR

Biological Process(BP), Cellular Component(CC) and Molecular Functions (MF)

**Table 3:** The enriched pathway terms of the up and down regulated differentially expressed genes

Pathway ID	Pathway name	*P*-value	FDR B&H	FDR B&Y	Bonferroni	Gene count	Gene
*Up regulated genes*							
1270244	Extracellular matrix organization	3.33E−08	1.80E−05	1.23E−04	1.80E−05	24	COL22A1, MFAP4, CMA1, COL14A1, COL1A1, COL8A2, COL9A1, COL9A2, COL10A1, FMOD, COL16A1, COMP, HAPLN1, ADAMTS14, CTSG, ASPN, ITGAL, ITGB7, CAPN6, TGFB2, P3H2, TLL2, LTBP2, LUM
1269201	Immunoregulatory interactions between a Lymphoid and a non-Lymphoid cell	8.13E−06	7.31E−04	5.03E−03	4.39E−03	13	SH2D1B, SLAMF7, SIGLEC8, ITGAL, ITGB7, KLRB1, CD1C, CD3D, CD3E, CD3G, CD247, CD40LG, SH2D1A
1269544	GPCR ligand binding	3.92E−04	1.51E−02	1.04E−01	2.12E−01	22	GNG8, PENK, F2RL2, AGTR2, APLNR, CXCL9, CCR7, CXCL11, OXER1, HTR2A, HTR2B, WNT10B, WNT9A, ACKR4, CRHBP, S1PR5, FZD2, CX3CR1, CXCL10, MCHR1, LHCGR, GPR68
1268749	Metabolism of Angiotensinogen to Angiotensins	5.26E−04	1.85E−02	1.27E−01	2.84E−01	4	CMA1, CTSG, ACE, GZMH
1269868	Muscle contraction	3.44E−02	4.03E−01	1.00E+00	1.00E+00	9	KCNIP1, RYR3, SCN2B, ATP1A4, ATP1B2, MYL1, KCNK17, NPPA, TNNI1
1269340	Hemostasis	6.57E−02	5.21E−01	1.00E+00	1.00E+00	20	GNG8, CEACAM3, F2RL2, SERPINE2, APOA1, GNA14, PDE5A, ATP1B2, IL2RB, CTSW, ISLR, ITGAL, LCK, TGFB2, LEFTY2, RASGRP1, CD2, P2RX6, CD48, HBB
1269171	Adaptive Immune System	1.32E−01	6.87E−01	1.00E+00	1.00E+00	23	NRG1, HLA-DQA1, ZAP70, SH2D1B, CARD11, SLAMF7, SIGLEC8, ITGAL, ITGB7, ASB18, IER3, MRC2, KLRB1, LCK, RASGRP1, CD1C, CD3D, CD3E, CD3G, CD247, FBXL16, CD40LG, SH2D1A
**Down regulated genes**							
1457780	Neutrophil degranulation	4.82E−06	3.14E−03	2.22E−02	3.14E−03	28	SERPINA3, HK3, ACP3, HMOX2, CHI3L1, S100A8, S100A9, FCER1G, MGST1, HP, FGR, ALOX5, ANPEP, FPR1, CR1, ARG1, SLC11A1, SIGLEC9, GPR84, HPSE, PTX3, CRISPLD2, CD177, SIGLEC14, RNASE2, TLR2, ELANE, MCEMP1
1269907	SLC-mediated transmembrane transport	6.91E−04	4.74E−02	3.35E−01	4.51E−01	16	HK3, SLC7A11, SLC4A7, SLC1A1, SLC2A1, SLC5A1, SLC11A1, SLC22A16, SLCO2A1, SLCO4A1, GCKR, LCN15, SLC9A7, SLC25A18, SLC38A4, RHAG
1269545	Class A/1 (Rhodopsin-like receptors)	8.72E−04	4.74E−02	3.35E−01	5.69E−01	17	OPN4, SAA1, AGTR1, CCR1, CNR1, P2RY12, FPR1, SSTR2, SSTR5, KNG1, GPR4, GPR183, RGR, S1PR3, EDN1, EDN2, EDNRB
1269340	Hemostasis	2.18E−03	7.11E−02	5.02E−01	1.00E+00	26	SERPINA3, CD109, SLC7A11, F5, F8, F13A1, SERPINB8, FCER1G, FGR, MERTK, SERPINF2, SELE, P2RY12, APOB, KIF18B, ATP2A2, NHLRC2, KNG1, PDE11A, CD177, DOCK9, GRB7, GRB14, THBS1, SERPINE1, SERPINA5
1269903	Transmembranetransport of small molecules	4.89E−03	1.28E−01	9.01E−01	1.00E+00	26	HK3, TRPC4, CFTR, HMOX2, SLC7A11, ABCB1, SLC4A7, AQP3, AQP4, SLC1A1, SLC2A1, SLC5A1, SLC11A1, SLC22A16, SLCO2A1, ATP2A2, GABRR2, SLCO4A1, GCKR, LCN15, SLC9A7, WNK3, SLC25A18, SLC38A4, RHAG, STEAP3
1269203	Innate Immune System	9.62E−03	1.96E−01	1.00E+00	1.00E+00	42	SERPINA3, EREG, HK3, MARK3, ACP3, CLEC7A, HMOX2, CHI3L1, S100A8, S100A9, SAA1, FCER1G, MGST1, FCGR3A, HP, GRAP2, PLA2G2A, FGF5, FGF10, FGR, ALOX5, ANPEP, FPR1, APOB, CR1, FCN3, ARG1, SLC11A1, SIGLEC9, GPR84, HPSE, PTX3, WASF1, CRISPLD2, CD177, SIGLEC14, LBP, RNASE2, TLR2, ELANE, IRAK3, MCEMP1
1269310	Cytokine Signaling in Immune system	8.42E−02	4.76E−01	1.00E+00	1.00E+00	23	EREG, MARK3, F13A1, SAA1, FGF5, CCR1, FGF10, ALOX5, IL17RB, FLT3, FPR1, SAMHD1, IL10, IL15RA, IL20RA, BCL6, IL18R1, IL1R2, SOCS3, LBP, IRAK3, IL1RL1, OSMR

### Protein–protein interaction (PPI) network and module analysis

**Fig. 3:**
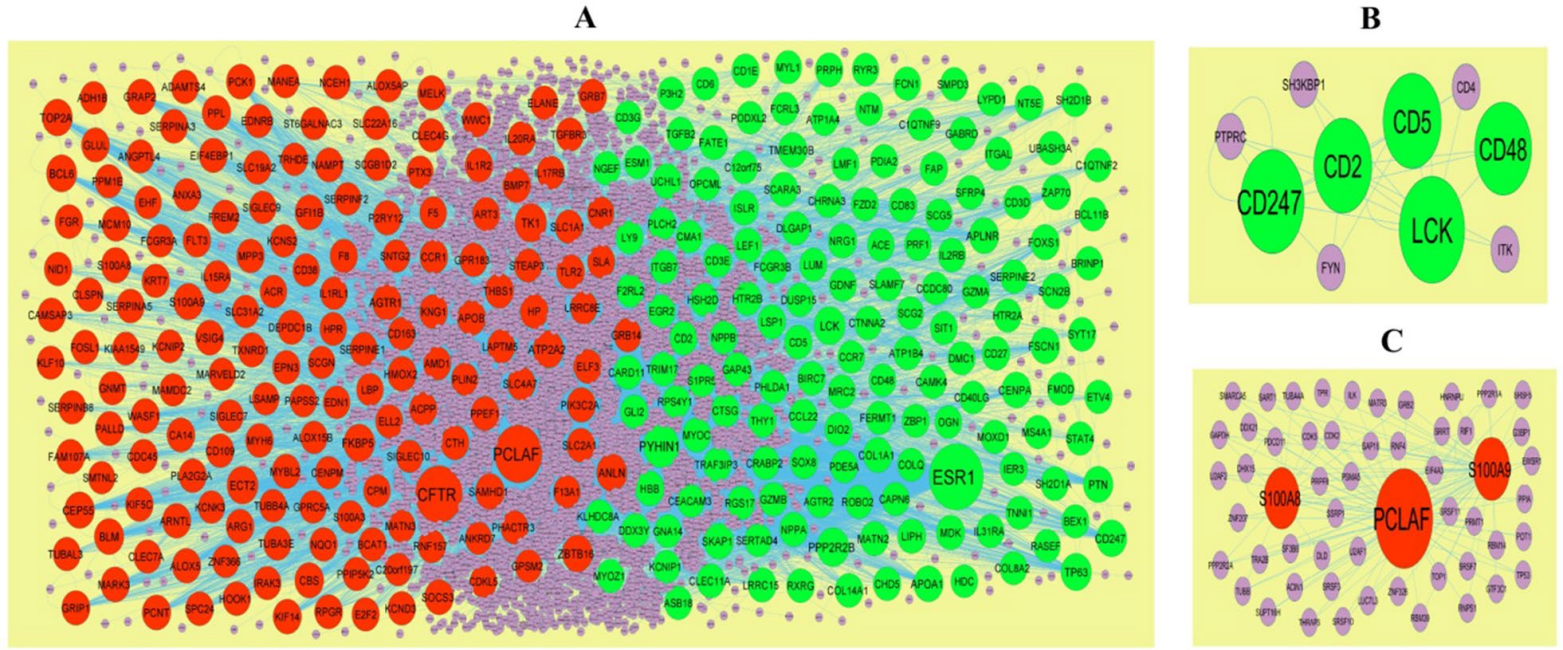
PPI network and the most significant modules of DEGs. **A** The PPI network of DEGs was constructed using Cytoscape. **B** The most significant module was obtained from PPI network with 4 nodes and 6 edges for up regulated genes. **C** The most significant module was obtained from PPI network with 6 nodes and 10 edges for down regulated genes. Up regulated genes are marked in green; down regulated genes are marked in red

Based on the HiPPIE interactome database, the PPI network for the DEGs (including 6541 nodes and 13,909 edges) was constructed (Fig. 3A). Up regulated gene with higher node degree, higher betweenness centrality, higher stress centrality and higher closeness centrality were as follows: ESR1, PYHIN1, PPP2R2B, LCK, TP63 and so on. Down regulated genes had higher node degree, higher betweenness centrality, higher stress centrality and higher closeness centrality were as follows PCLAF, CFTR, TK1, ECT2, FKBP5 and so on. The node degree, betweenness centrality, stress centrality and closeness centrality are listed in Table 4.

**Table 4:** Topology table for up and down regulated genes

Regulation	Node	Degree	Betweenness	Stress	Closeness
Up	ESR1	1094	0.250896	7.4E+08	0.392769
Up	PYHIN1	342	0.054258	1.1E+08	0.339882
Up	PPP2R2B	199	0.023115	35,268,762	0.346839
Up	LCK	162	0.033618	18,589,354	0.353915
Up	TP63	142	0.018577	39,426,608	0.319664
Up	CD247	129	0.013832	18,625,274	0.317784
Up	PTN	105	0.016638	36,892,166	0.300662
Up	APLNR	103	0.016611	40,715,208	0.288093
Up	APOA1	100	0.018834	13,935,332	0.315866
Up	CENPA	98	0.009846	31,131,318	0.301786
Up	SKAP1	97	0.01599	10,109,482	0.313338
Up	FSCN1	88	0.010226	9,387,016	0.335367
Up	SCN2B	86	0.01089	24,233,242	0.284756
Up	TMEM30B	79	0.016411	10,490,230	0.270807
Up	FOXS1	79	0.009408	26,398,078	0.293247
Up	COL1A1	76	0.010472	9,315,670	0.308098
Up	ZAP70	75	0.007096	5,315,196	0.317614
Up	UCHL1	74	0.009753	9,381,024	0.331525
Up	HBB	72	0.009984	14,209,592	0.304767
Up	NRG1	70	0.01151	13,449,066	0.29526
Up	LEF1	61	0.007998	18,260,094	0.290744
Up	NT5E	60	0.009599	11,470,634	0.301563
Up	MDK	59	0.006889	7,533,352	0.305822
Up	ISLR	58	0.010139	9,992,806	0.294012
Up	FATE1	57	0.011609	9,248,788	0.281581
Up	LRRC15	56	0.010069	4,772,792	0.299094
Up	MATN2	54	0.004491	9,580,600	0.286792
Up	LIPH	54	0.008197	6,873,658	0.282347
Up	MYOC	49	0.005138	12,313,276	0.291029
Up	SCARA3	49	0.006492	11,494,220	0.290151
Up	NPPA	46	0.009502	4,868,574	0.307143
Up	CD83	43	0.005149	5,484,906	0.272841
Up	COL14A1	41	0.006129	5,699,128	0.292893
Up	CTSG	40	0.003821	1,913,996	0.296155
Up	SFRP4	40	0.004006	7,876,966	0.282518
Up	TRAF3IP3	38	0.006447	4,263,002	0.290602
Up	CLEC11A	38	0.005085	3,520,012	0.283596
Up	ATP1B4	38	0.005722	2,476,482	0.245939
Up	CD3E	37	0.003892	1,537,326	0.297624
Up	SH2D1A	37	0.003969	2,117,220	0.308113
Up	DDX3Y	37	0.003754	1,632,916	0.32095
Up	PRPH	37	0.001871	1,994,266	0.301494
Up	BIRC7	35	0.004835	2,567,254	0.28549
Up	CARD11	35	0.002249	2,510,852	0.292173
Up	RXRG	35	0.002394	7,134,478	0.26225
Up	CCL22	34	0.005706	3,916,024	0.281423
Up	CD27	33	0.003915	2,076,446	0.294263
Up	GZMB	32	0.003277	6,141,522	0.285602
Up	THY1	32	0.003561	1,793,684	0.291509
Up	CHRNA3	32	0.004247	3,415,720	0.236631
Up	LSP1	32	0.003371	5,734,010	0.281836
Up	IL2RB	31	0.002351	1,938,172	0.305808
Up	HTR2B	30	0.004018	2,165,998	0.27434
Up	DLGAP1	29	0.003816	6,072,882	0.277754
Up	TRIM17	29	0.002943	5,543,810	0.275137
Up	CTNNA2	29	0.003554	3,511,510	0.30254
Up	SERPINE2	28	0.002577	3,031,536	0.27426
Up	CD1E	28	0.003282	3,419,392	0.255229
Up	MRC2	28	0.003395	2,950,548	0.296276
Up	C1QTNF2	28	0.003203	2,505,388	0.270147
Up	SH2D1B	27	0.001864	1,043,570	0.29028
Up	BRINP1	27	0.001516	4,402,948	0.27726
Up	PDIA2	27	0.001967	2,895,182	0.286453
Up	CHD5	27	0.001918	5,223,636	0.286503
Up	FAP	27	0.003531	5,820,086	0.268583
Up	IL31RA	26	0.002073	1,606,954	0.263603
Up	GAP43	25	0.002745	2,793,268	0.279858
Up	CD5	25	0.001695	655,904	0.295861
Up	UBASH3A	25	0.001652	1,347,264	0.290951
Up	ROBO2	25	0.002959	3,726,616	0.267048
Up	ITGB7	24	0.002686	3,351,660	0.276124
Up	HTR2A	24	0.002571	2,472,010	0.275833
Up	MOXD1	24	0.002391	2,492,756	0.259926
Up	ASB18	24	9.77E−04	3,202,162	0.273001
Up	CD2	23	0.002221	926,408	0.287954
Up	BCL11B	23	8.18E−04	1,888,298	0.28836
Up	STAT4	23	0.001786	2,435,506	0.277166
Up	NGEF	23	0.001809	1,548,206	0.277636
Up	SMPD3	23	0.002518	2,175,774	0.281
Up	FZD2	22	0.003673	3,239,390	0.250335
Up	DUSP15	22	0.001253	2,474,532	0.284472
Up	CD3D	21	0.001725	1,111,132	0.288589
Up	SYT17	21	0.002549	2,482,574	0.285802
Up	FCGR3B	21	0.002748	1,492,022	0.282286
Up	EGR2	21	0.002934	3,438,856	0.266406
Up	ZBP1	21	0.001876	2,664,938	0.26006
Up	CAMK4	21	0.001773	3,472,134	0.272716
Up	DMC1	20	0.002511	4,659,098	0.254277
Up	GDNF	20	0.002515	3,274,958	0.244751
Up	FCN1	20	0.002571	1,243,380	0.236742
Up	LUM	20	0.002276	1,515,870	0.283903
Up	GZMA	20	0.001051	3,258,230	0.276498
Up	TGFB2	20	0.002259	1,632,566	0.277119
Up	SLAMF7	20	0.00211	1,035,482	0.271708
Up	MS4A1	20	0.002857	1,169,078	0.288398
Up	ETV4	20	0.001747	1,674,080	0.301883
Up	GLI2	20	0.001398	2,977,194	0.285902
Up	PHLDA1	19	4.37E−04	818,256	0.298194
Up	COL8A2	19	0.00147	1,089,762	0.273835
Up	GABRD	19	0.002826	2,629,544	0.25748
Up	LMF1	19	0.004342	2,024,228	0.265132
Up	F2RL2	19	0.001554	790,338	0.282933
Up	LYPD1	19	0.003123	3,995,458	0.266276
Up	CAPN6	19	0.001415	3,046,736	0.267802
Up	SOX8	19	0.003361	2,763,024	0.251306
Up	IER3	18	0.001921	3,613,164	0.282982
Up	BEX1	18	0.001034	1,286,206	0.273606
Up	COLQ	18	0.001173	1,414,826	0.261234
Up	NTM	18	0.00284	2,486,684	0.275102
Up	RPS4Y1	18	0.001013	1,200,768	0.287713
Up	FERMT1	18	0.001713	4,279,868	0.270315
Up	RGS17	18	0.002928	3,868,106	0.249895
Up	TNNI1	17	0.001349	1,550,004	0.266765
Up	MYOZ1	17	0.00128	2,111,156	0.283203
Up	KLHDC8A	17	0.001147	7,007,508	0.251036
Up	MYL1	17	7.90E−04	1,213,666	0.289945
Up	DIO2	16	0.001161	1,959,228	0.279416
Up	ITGAL	16	0.001182	1,521,116	0.271527
Up	CRABP2	16	4.13E−04	675,182	0.272171
Up	HSH2D	16	0.001425	889,856	0.26034
Up	CD48	3	0	0	0.265422
Up	CD3G	2	0	0	0.23833
Up	LY9	2	0	0	0.240141
Up	SIT1	2	0	0	0.264221
Up	ATP1A4	2	1.16E−04	79,140	0.235049
Up	FMOD	2	3.96E−05	20,526	0.240707
Up	CCDC80	2	3.58E−05	499,704	0.288908
Up	CCR7	2	0	0	0.244312
Up	KCNIP1	1	0	0	0.219995
Up	CD6	1	0	0	0.22832
Up	FCRL3	1	0	0	0.241062
Up	SERTAD4	1	0	0	0.257531
Up	PRF1	1	0	0	0.222162
Up	C1QTNF9	1	0	0	0.226548
Up	OPCML	1	0	0	0.215756
Up	ESM1	1	0	0	0.213551
Up	CD40LG	1	0	0	0.240053
Up	S1PR5	1	0	0	0.24224
Up	AGTR2	1	0	0	0.259256
Up	NPPB	1	0	0	0.211726
Up	SCG5	1	0	0	0.238721
Up	PDE5A	1	0	0	0.243548
Up	RYR3	1	0	0	0.274755
Up	RASEF	1	0	0	0.274755
Up	PODXL2	1	0	0	0.213106
Up	OGN	1	0	0	0.226548
Up	PLCH2	1	0	0	0.238721
Up	SCG2	1	0	0	0.267704
Up	P3H2	1	0	0	0.207132
Up	C12orf75	1	0	0	0.217608
Up	ACE	1	0	0	0.241159
Up	GNA14	1	0	0	0.217608
Up	HDC	1	0	0	0.216614
Up	CMA1	1	0	0	0.226713
Up	CEACAM3	1	0	0	0.265519
Down	PCLAF	817	0.135529	4.95E+08	0.365547
Down	CFTR	800	0.168404	4.5E+08	0.378823
Down	TK1	188	0.034997	43,663,230	0.331089
Down	ECT2	164	0.020509	39,431,940	0.325989
Down	FKBP5	157	0.028064	15,963,868	0.346288
Down	ANLN	153	0.021564	38,168,832	0.325066
Down	ATP2A2	148	0.027131	19,656,040	0.363859
Down	BCL6	142	0.022279	29,419,916	0.314181
Down	TOP2A	132	0.018571	16,838,266	0.361426
Down	ZBTB16	132	0.025165	14,500,206	0.349976
Down	S100A9	124	0.01355	11,186,464	0.352219
Down	CEP55	123	0.019583	21,505,878	0.316891
Down	BLM	108	0.014259	18,458,556	0.321945
Down	AGTR1	100	0.019518	14,083,216	0.313564
Down	SAMHD1	94	0.011463	12,340,270	0.337357
Down	S100A8	88	0.011637	8,662,548	0.361486
Down	GRAP2	86	0.011721	16,819,438	0.305936
Down	CBS	83	0.011248	20,466,334	0.301591
Down	SOCS3	83	0.011071	9,067,820	0.324888
Down	GFI1B	80	0.011791	21,469,034	0.299012
Down	APOB	78	0.014102	9,290,092	0.319133
Down	PCK1	77	0.004102	12,732,476	0.305408
Down	MARK3	76	0.008497	19,265,788	0.304512
Down	HMOX2	75	0.011098	15,258,770	0.312053
Down	PCNT	74	0.011297	9,190,430	0.312261
Down	PIK3C2A	69	0.005568	8,768,122	0.313053
Down	KIF14	69	0.01035	12,506,564	0.304668
Down	WASF1	67	0.009478	18,219,554	0.29633
Down	ARNTL	65	0.00974	19,494,854	0.295526
Down	ALOX5	65	0.010921	7,343,824	0.306424
Down	MCM10	64	0.006773	8,807,396	0.306438
Down	THBS1	64	0.008915	6,203,038	0.312694
Down	VSIG4	64	0.010353	10,534,518	0.302037
Down	WWC1	64	0.007241	14,604,594	0.301647
Down	MELK	63	0.008554	18,629,232	0.283953
Down	P2RY12	63	0.008962	9,063,088	0.286641
Down	PPL	62	0.007851	16,137,388	0.297313
Down	MYBL2	59	0.006189	16,766,208	0.291964
Down	FAM107A	59	0.006172	12,510,302	0.289688
Down	GRIP1	58	0.008069	3,384,760	0.320055
Down	ELF3	56	0.004945	7,205,142	0.309118
Down	PALLD	55	0.00509	15,467,012	0.293274
Down	CTH	54	0.007754	5,179,060	0.296908
Down	EIF4EBP1	53	0.005367	9,748,852	0.303946
Down	KNG1	53	0.007017	4,204,766	0.304243
Down	GLUL	51	0.00728	10,616,752	0.30116
Down	SLC2A1	51	0.004557	8,307,646	0.303974
Down	HP	51	0.006741	3,398,298	0.314741
Down	RPGR	50	0.004441	10,097,488	0.29384
Down	TLR2	50	0.00754	8,322,330	0.294595
Down	GRB7	49	0.004147	4,832,846	0.308113
Down	PPEF1	49	0.001983	3,327,232	0.298276
Down	TXNRD1	49	0.00627	2,860,122	0.328561
Down	NAMPT	48	0.005035	10,420,710	0.290538
Down	BMP7	47	0.007622	4,442,306	0.286981
Down	CA14	47	0.005218	4,884,858	0.279189
Down	CCR1	46	0.008305	11,217,842	0.27812
Down	CDC45	45	0.004479	3,231,162	0.30618
Down	ARG1	45	0.004931	2,347,856	0.32381
Down	SPC24	43	0.005356	6,589,710	0.294834
Down	FGR	43	0.003434	3,020,950	0.303452
Down	KIF5C	42	0.004876	2,365,086	0.319586
Down	IL1R2	42	0.006825	9,489,620	0.289265
Down	SERPINA3	42	0.005518	4,047,110	0.293168
Down	DEPDC1B	42	0.002978	9,632,346	0.260838
Down	SLC4A7	41	0.006314	2,752,106	0.316232
Down	SERPINA5	41	0.003604	13,750,404	0.273206
Down	MPP3	40	0.008262	9,328,402	0.297003
Down	NCEH1	40	0.009405	3,554,728	0.304214
Down	SLC1A1	38	0.008678	2,278,154	0.320573
Down	CLSPN	38	0.003668	3,801,300	0.294343
Down	BCAT1	38	0.0052	9,066,238	0.269657
Down	MYH6	38	0.005049	1,731,786	0.308578
Down	IL20RA	37	0.005252	8,769,348	0.267901
Down	HOOK1	37	0.005558	7,195,380	0.279177
Down	FLT3	37	0.002948	1,938,440	0.292408
Down	ADAMTS4	37	0.005524	2,338,476	0.307519
Down	CAMSAP3	36	0.003339	4,795,892	0.29578
Down	PLA2G2A	35	0.003637	1,686,594	0.300565
Down	FOSL1	34	0.004151	10,955,318	0.269402
Down	NQO1	34	0.001945	5,351,260	0.289201
Down	ELANE	34	0.005024	2,289,646	0.302834
Down	KCND3	34	0.002555	9,153,178	0.28203
Down	EPN3	34	0.005073	7,423,282	0.280302
Down	GPR183	34	0.003642	4,243,792	0.256783
Down	CD109	34	0.006381	3,655,940	0.303565
Down	TUBA3E	34	0.003459	6,711,886	0.289048
Down	TGFBR3	33	0.005143	1,983,082	0.267386
Down	NID1	33	0.004536	1,702,236	0.311503
Down	STEAP3	33	0.004665	2,788,716	0.285365
Down	AMD1	32	0.005714	3,154,782	0.29099
Down	EDNRB	31	0.003092	7,273,156	0.265368
Down	IL17RB	31	0.004227	6,381,040	0.261527
Down	SLC19A2	30	0.004653	2,505,974	0.281302
Down	SLC22A16	30	0.004545	3,831,872	0.240618
Down	PHACTR3	29	0.002193	6,417,862	0.280976
Down	LAPTM5	29	0.003298	2,735,158	0.274317
Down	ANGPTL4	29	0.003467	1,447,446	0.325163
Down	PPM1E	29	0.002894	5,733,032	0.270427
Down	E2F2	28	0.002816	5,508,320	0.28041
Down	SERPINE1	28	0.001474	2,497,574	0.271302
Down	ACPP	28	0.003084	2,749,550	0.291223
Down	KRT7	28	0.002861	1,288,592	0.315774
Down	SERPINB8	28	0.002944	3,167,812	0.28186
Down	FREM2	28	0.003954	3,395,758	0.276661
Down	RNF157	28	0.002172	6,196,626	0.265551
Down	PPIP5K2	28	0.003886	8,572,108	0.270014
Down	F8	27	0.002839	4,879,016	0.274836
Down	TUBAL3	27	0.002055	1,052,840	0.318915
Down	ELL2	26	0.003971	6,281,508	0.255859
Down	GRB14	25	0.002326	3,092,024	0.28378
Down	IRAK3	25	0.00257	6,900,170	0.265897
Down	MANEA	25	0.004508	5,075,608	0.263869
Down	CLEC7A	25	0.004246	4,293,212	0.277095
Down	KLF10	24	0.001607	3,013,994	0.281339
Down	GNMT	24	0.00165	3,015,768	0.269136
Down	ART3	24	0.002904	2,401,360	0.255748
Down	LRRC8E	24	0.003739	4,188,308	0.288665
Down	SLA	23	0.001714	1,003,510	0.289329
Down	CLEC4G	23	0.002667	2,376,260	0.277495
Down	TUBB4A	5	1.28E−04	181,440	0.250652
Down	CD38	4	0	0	0.268176
Down	FCGR3A	4	1.01E−04	73,756	0.268385
Down	F5	3	8.10E−07	708	0.248641
Down	EHF	2	7.11E−06	4180	0.254842
Down	KIAA1549	2	4.16E−04	193,604	0.261391
Down	S100A3	2	1.84E−05	45,822	0.254376
Down	ADH1B	2	3.40E−05	28,184	0.233546
Down	PAPSS2	2	1.05E−05	8726	0.251868
Down	PTX3	1	0	0	0.19143
Down	IL15RA	1	0	0	0.234199
Down	EDN1	1	0	0	0.209723
Down	SERPINF2	1	0	0	0.232451
Down	ZNF366	1	0	0	0.282018
Down	ACR	1	0	0	0.214588
Down	MATN3	1	0	0	0.222881
Down	CNR1	1	0	0	0.216205
Down	LBP	1	0	0	0.240053
Down	ALOX5AP	1	0	0	0.23456
Down	SCGN	1	0	0	0.23353
Down	MAMDC2	1	0	0	0.248745
Down	CDKL5	1	0	0	0.219891
Down	CENPM	1	0	0	0.231833
Down	KCNIP2	1	0	0	0.219995
Down	CPM	1	0	0	0.24533
Down	GPSM2	1	0	0	0.245855
Down	LSAMP	1	0	0	0.215756
Down	KCNK3	1	0	0	0.219353
Down	ALOX15B	1	0	0	0.234981
Down	ST6GALNAC3	1	0	0	0.233263
Down	GPRC5A	1	0	0	0.274755
Down	SLC31A2	1	0	0	0.215287
Down	MARVELD2	1	0	0	0.218671
Down	SNTG2	1	0	0	0.229
Down	TRHDE	1	0	0	0.208786
Down	SIGLEC7	1	0	0	0.245229
Down	SMTNL2	1	0	0	0.265519
Down	ANXA3	1	0	0	0.274755
Down	F13A1	1	0	0	0.248745
Down	ANKRD7	1	0	0	0.233438
Down	KCNS2	1	0	0	0.219721
Down	SIGLEC9	1	0	0	0.227565
Down	SIGLEC10	1	0	0	0.282018
Down	C20orf197	1	0	0	0.282018
Down	SCGB1D2	1	0	0	0.226548
Down	IL1RL1	1	0	0	0.21698
Down	PLIN2	1	0	0	0.241935
Down	CD163	1	0	0	0.239403
Down	HPR	1	0	0	0.240053

Additionally, two significant modules, including module 1 (10 nodes and 24 edges) and module 2 (5 nodes and 10 edges) (Fig. 3B) and module 3 (55 nodes and 115 edges), were acquired by PEWCC1 plug-in (Fig. 3C). Furthermore, GO terms and REACTOME pathways were significantly enriched by module 1, including adaptive immune system, immunoregulatory interactions between a lymphoid and a non-lymphoid cell, hemostasis, biological adhesion and regulation of immune system process. Meanwhile, the nodes in module 2 were significantly enriched in GO terms and REACTOME pathways, including neutrophil degranulation and secretion.

### Target gene—miRNA regulatory network construction

**Fig. 4:**
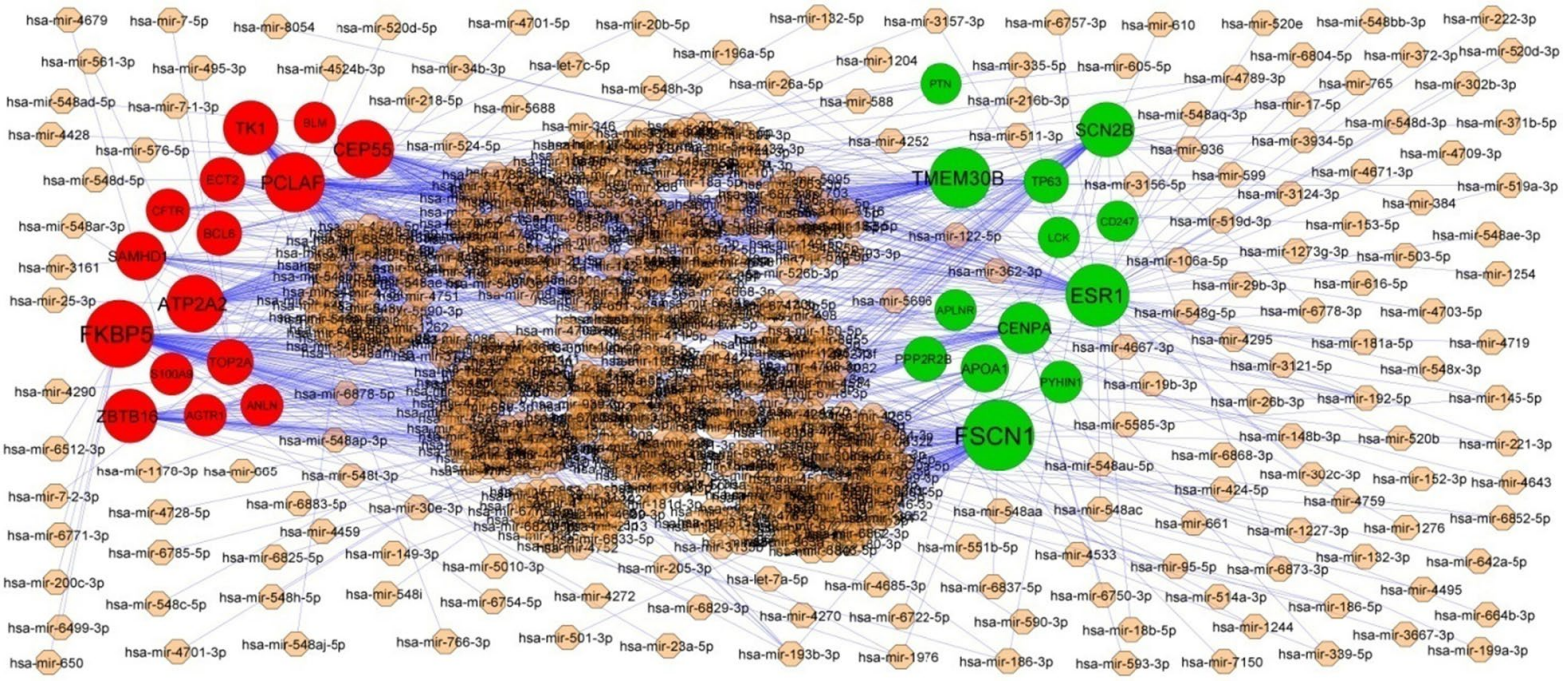
Target gene—miRNA regulatory network between target genes. The light orange color diamond nodes represent the key miRNAs; up regulated genes are marked in green; down regulated genes are marked in red

Associations between 2063 miRNAs and their 319 target genes were collected from the target gene—miRNA regulatory network (Fig. 4). MiRNAs of hsa-mir-4533, hsa-mir-548ac, hsa-mir-548i, hsa-mir-5585-3p, hsa-mir-6750-3p, hsa-mir-200c-3p, hsa-mir-1273 g-3p, hsa-mir-1244, hsa-mir-4789-3p and hsa-mir-766-3p, which exhibited a high degree of interaction, were selected from this network. Furthermore, the results also showed that FSCN1 was the target of hsa-mir-4533, ESR1 was the target of hsa-mir-548ac, TMEM30B was the target of hsa-mir-548i, SCN2B was the target of hsa-mir-5585-3p, CENPA was the target of hsa-mir-6750-3p, FKBP5 was the target of hsa-mir-200c-3p, PCLAF was the target of hsa-mir-1273g-3p, CEP55 was the target of hsa-mir-1244, ATP2A2 was the target of hsa-mir-4789-3p and TK1 was the target of hsa-mir-766-3p, and are listed in Table 5.

**Table 5:** miRNA—target gene and TF—target gene interaction

Regulation	Target Genes	Degree	MicroRNA	Regulation	Target Genes	Degree	TF
Up	FSCN1	99	hsa-mir-4533	Up	FSCN1	62	ESRRA
Up	ESR1	72	hsa-mir-548ac	Up	APOA1	48	RERE
Up	TMEM30B	64	hsa-mir-548i	Up	COL1A1	21	HMG20B
Up	SCN2B	46	hsa-mir-5585-3p	Up	HBB	16	THRAP3
Up	CENPA	35	hsa-mir-6750-3p	Up	LCK	15	ATF1
Up	APOA1	22	hsa-mir-6722-5p	Up	FOXS1	14	YBX1
Up	PPP2R2B	14	hsa-mir-149-3p	Up	CENPA	10	SAP30
Up	TP63	12	hsa-mir-1178-3p	Up	SCN2B	5	RCOR2
Up	PYHIN1	5	hsa-mir-205-3p	Up	TMEM30B	5	ZNF24
Up	APLNR	2	hsa-mir-10b-5p	Up	APLNR	4	FOXJ2
Up	PTN	1	hsa-mir-155-5p	Up	NRG1	2	SUZ12
Up	LCK	1	hsa-mir-335-5p	Up	PTN	2	L3MBTL2
Up	CD247	1	hsa-mir-346	Up	UCHL1	2	MAZ
Down	FKBP5	88	hsa-mir-200c-3p	Up	ESR1	1	EZH2
Down	PCLAF	62	hsa-mir-1273g-3p	Up	ZAP70	1	ZFX
Down	CEP55	57	hsa-mir-1244	Down	SOCS3	48	MXD3
Down	ATP2A2	55	hsa-mir-4789-3p	Down	BCL6	44	ARID4B
Down	TK1	45	hsa-mir-766-3p	Down	FKBP5	43	CBFB
Down	ZBTB16	43	hsa-mir-1976	Down	ANLN	38	TAF7
Down	SAMHD1	26	hsa-mir-3124-3p	Down	ATP2A2	35	CREM
Down	TOP2A	17	hsa-mir-186-5p	Down	CBS	31	IKZF1
Down	BCL6	13	hsa-mir-339-5p	Down	BLM	19	ZNF501
Down	ECT2	13	hsa-mir-132-3p	Down	ECT2	15	KLF16
Down	CFTR	9	hsa-mir-145-5p	Down	CEP55	10	FOSL2
Down	S100A9	7	hsa-mir-4679	Down	GRAP2	10	CEBPD
Down	AGTR1	5	hsa-mir-410-3p	Down	ZBTB16	4	TRIM28
Down	ANLN	5	hsa-mir-503-5p	Down	S100A8	3	STAT3
Down	BLM	3	hsa-mir-193b-3p	Down	S100A9	2	CEBPG
				Down	AGTR1	1	EZH2

### Target gene—TF regulatory network construction

**Fig. 5:**
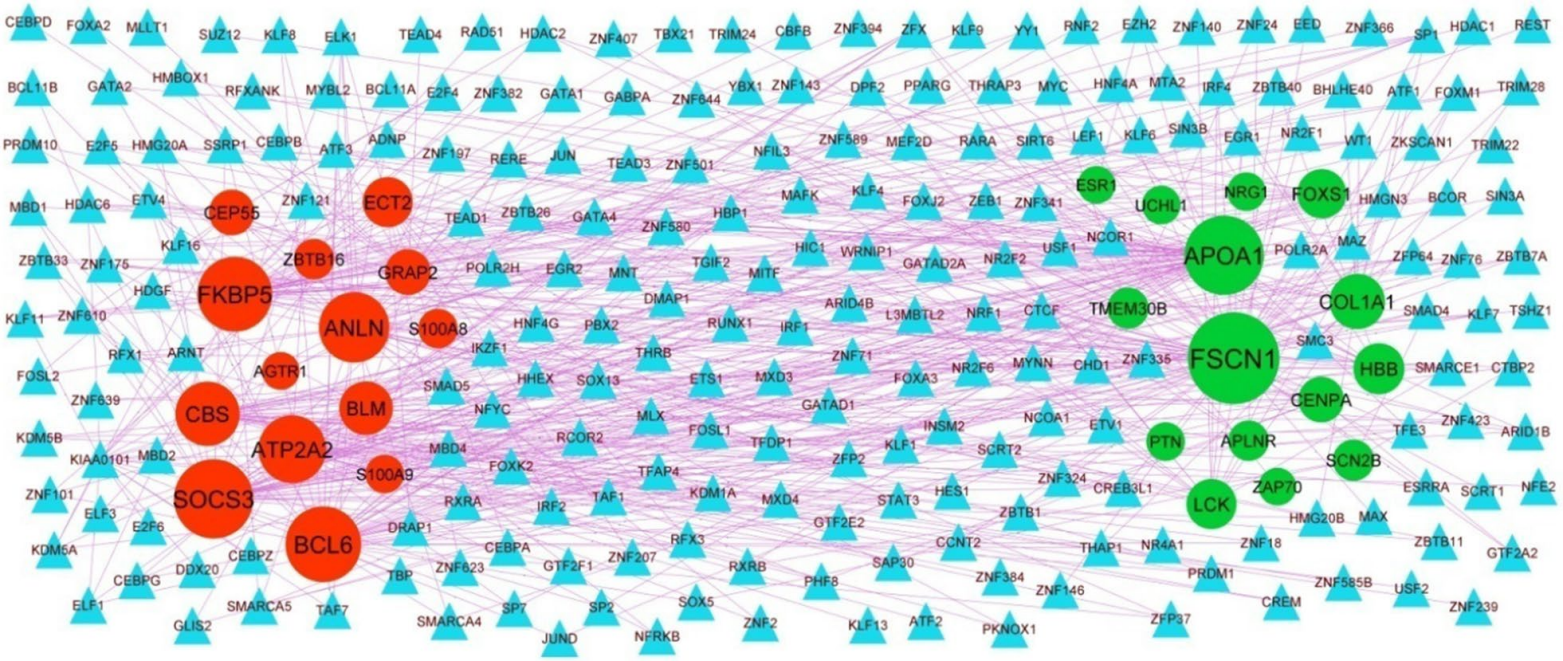
Target gene—TF regulatory network between target genes. The sky blue color triangle nodes represent the key TFs; up regulated genes are marked in green; down regulated genes are marked in red

Associations between 330 TFs and their 247 target genes were collected from the target gene—TF regulatory network (Fig. 5). TFs of ESRRA, RERE, HMG20B, THRAP3, ATF1, MXD3, ARID4B, CBFB, TAF7 and CREM, which exhibited a high degree of interaction, were selected from this network. Furthermore, the results also showed that FSCN1 was the target of ESRRA, APOA1 was the target of RERE, COL1A1 was the target of HMG20B, HBB was the target of THRAP3, LCK was the target of ATF1, SOCS3 was the target of MXD3, BCL6 was the target of ARID4B, FKBP5 was the target of CBFB, ANLN was the target of TAF7 and ATP2A2 was the target of CREM, and are listed in Table 5.

### Receiver operating characteristic (ROC) curve analysis

**Fig. 6:**
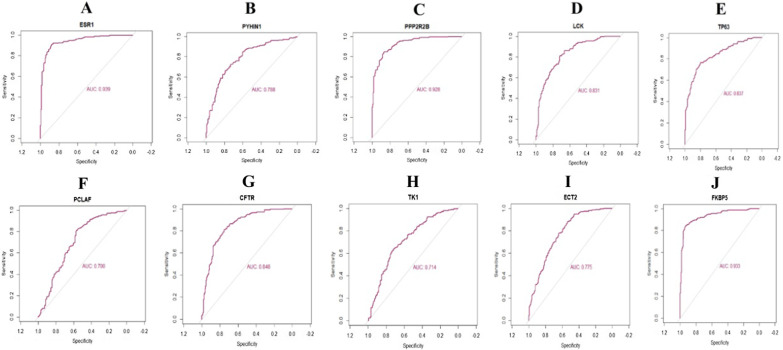
ROC curve analyses of hub genes. **A** ESR1, **B** PYHIN1, **C** PPP2R2B, **D** LCK, **E** TP63, **F** PCLAF, **G** CFTR, **H** TK1, **I** ECT2, **J** FKBP5

First of all, we performed the ROC curve analysis among 10 hub genes based on the GSE141910. The results showed that ESR1, PYHIN1, PPP2R2B, LCK, TP63, PCLAF, CFTR, TK1, ECT2 and FKBP5 achieved an AUC value of > 0.7, demonstrating that these ten genes have high sensitivity and specificity for HF, suggesting they can be served as biomarkers for the diagnosis of HF (Fig. 6).

### RT-PCR analysis

**Fig. 7:**
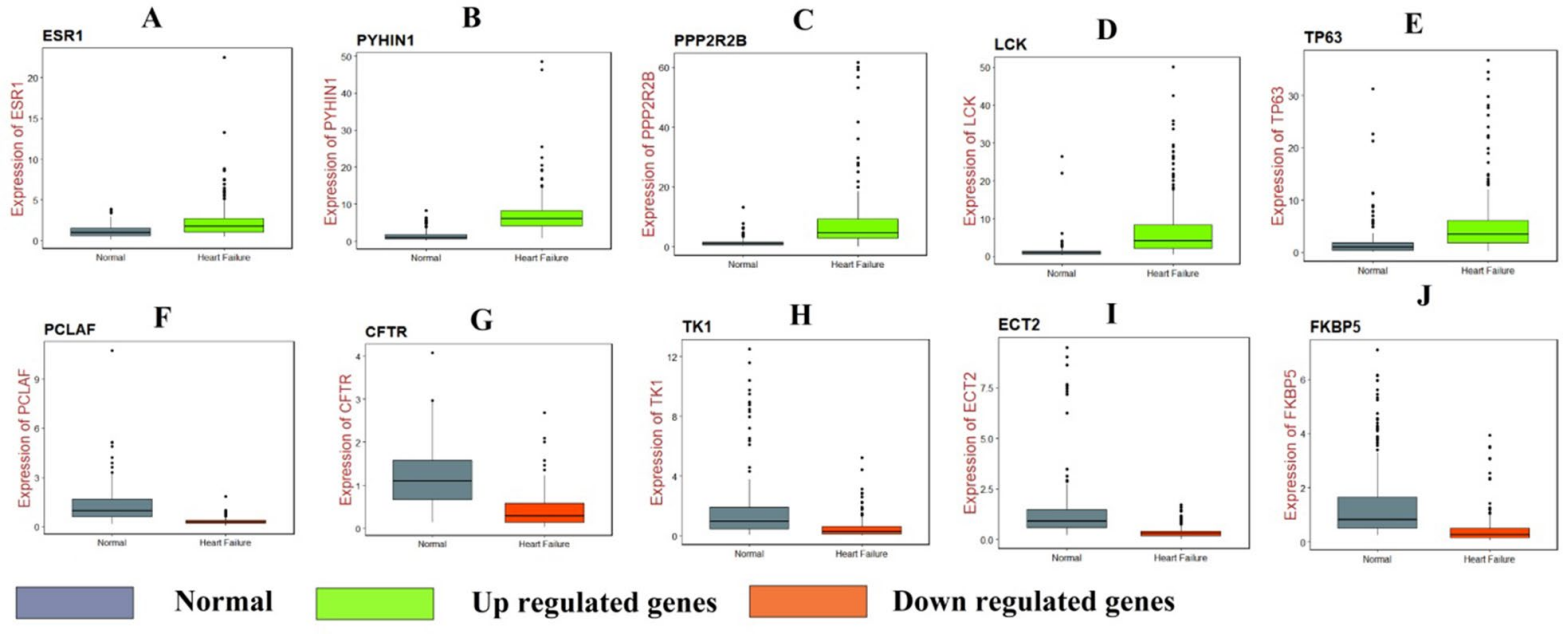
RT-PCR analyses of hub genes. **A** ESR1, **B** PYHIN1, **C** PPP2R2B, **D** LCK, **E** TP63, **F** PCLAF, **G**) CFTR **H** TK1, **I** ECT2, **J** FKBP5

RT-PCR was used to validate the hub genes between normal and HF cell lines. The results suggested that the mRNA expression level of ESR1, PYHIN1, PPP2R2B, LCK and TP63 were significantly increased in HF compared with that in normal, while PCLAF, CFTR, TK1, ECT2 and FKBP5 were significantly decreased in HF compared with that in normal and are shown in Fig. 7.

### Identification of candidate small molecules

**Fig. 8:**
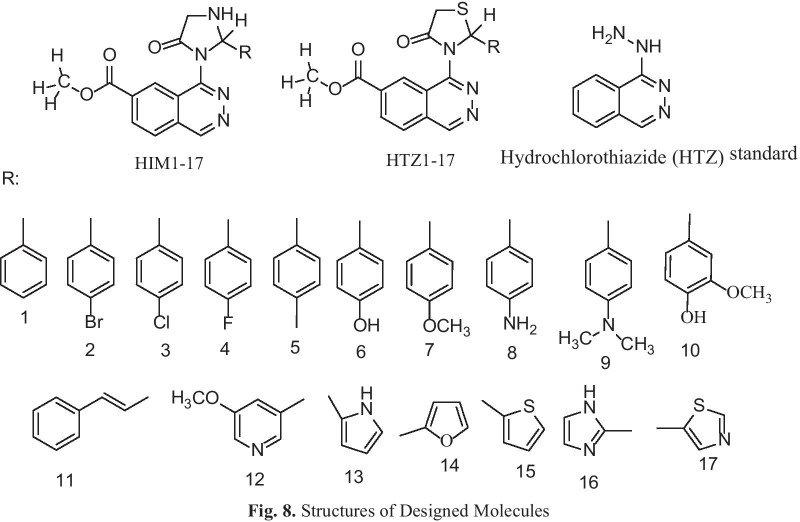
Structures of designed molecules

In the present study docking simulations are performed to spot the active site and foremost interactions accountable for complex stability with the receptor binding sites. In heart failure recognized over expressed genes and their proteins of x-ray crystallographic structure are chosen from PDB for docking studies. Most generally, medications containing benzothiadiazine ring hydrochlorothiazide are used in heart failure either alone or in conjunction with other drugs, based on this the molecules containing heterocyclic ring of benzothiadiazine are designed and hydrochlorothiazide is uses as a reference standard. Docking experiments using Sybyl-X 2.1.1. drug design perpetual software were used on the designed molecules. Docking studies were performed in order to understand the biding interaction of standard hydrochlorothiazide and designed molecules on over expressed protein. The X- RAY crystallographic structure of one proteins from each over expressed genes of ESR1, LCK, PPP2R2B, PYHIN1, TP63 and their co-crystallised protein of PDB code 4PXM, 1KSW, 2HV7, 3VD8 and 6RU6 respectively were selected for the docking studies to identify and predict the potential molecule based on the binding score with the protein and successful in heart failure. For the docking tests, a total of 34 molecules were built and the molecule with binding score greater than 5 is believed to be good. The designed molecules obtained docking score of 5 to 7 were HIM10, HTZ5, HIM6, HTZ31, HIM3, HIM14, HIM1, HIM7 and HIM11, HIM16, HTZ9, HIM17, HIM12, HTZ12, HIM6, HTZ7, HIM10, HTZ3 and HIM8, HTZ9, HIM6, HIM4, HIM13, HTZ16, HIM9, HIM7, HTZ5, HIM16, HTZ7, HIM10, HIM5, HIM12, HIM15, HTZ12, HIM3, HIM14 and HIM14, HIM6, HIM17, HTZ7, HIM10, HIM1, HTZ9, HIM3, HIM16, HIM15, HIM8, HIM9, HIM7, HTZ10, HTZ3, HTZ5, HTZ1, HIM13, HTZ4, HIM11, HTZ12, HTZ14, HIM2 and HIM7, HTZ13, HTZ5, HIM15, HIM12, HIM6, HTZ11, HIM14, HTZ9, HIM11, HIM13, HIM9, HIM8, HIM10, HIM1, HIM5, HIM4, HTZ12, HIM2, HIM17, HIM3, HTZ1, HTZ8, HIM3, HTZ14, HTZ3 with proteins 4PXM and, 1KSW and 2HV7 and 3VD8 and 6RUR respectively (Fig. 8). The molecules obtained binding score of less than 5 were HTZ13, HTZ12, HTZ10, HIM3, HIM15, HIM16, HIM13, HIM8, HTZ16, HIM2, HIM4, HIM17, HTZ17, HIM11, HTZ5, HTZ3, HIM9, HTZ15, HTZ5, HTZ9, HTZ11, HIM5, HTZ8 and HTZ14, HIM14, HTZ13, HIM13, HTZ16, HIM2, HIM3, HTZ10, HIM7, HIM1, HTZ1, HTZ4, HIM8, HIM5, HTZ2, HIM9, HTZ5, HTZ15, HTZ3, HIM4, HIM15, HTZ17, HTZ8, HTZ11 and HTZ14, HIM2, HIM1, HTZ11, HIM17, HTZ13, HTZ4, HTZ2, HIM3, HTZ15, HTZ8, HTZ17, HTZ1, HTZ3 and HTZ8, HIM4, HTZ16, HTZ15, HIM5, HTZ11, HTZ13, HIM3, HTZ17, HTZ2 and HTZ7, HTZ4, HTZ2, HTZ17, HTZ15 with proteins 4PXM and, 1KSW and 2HV7 and 3VD8 and 6RUR respectively. The molecules obtained very less binding score are HTZ1, HIM12, HTZ2, HTZ4 with protein 4PXM and the standard hydrochlorothiazide (HTZ) obtained less binding score with all proteins, the values are depicted in Table 6.

**Table 6:** Docking results of Designed Molecules on Over Expressed Proteins

Sl. No/ Code	Over expressed gene: ESR1			Over expressed gene: LCK			Over expressed gene: PPP2R2B			Over expressed gene: PYHIN 1			Over expressed gene: TP63		
	PDB: 4PXM			PDB:1KSW			PDB: 2HV7			PDB: 3VD8			PDB: 6RU6		
	Total Score	Crash (-Ve)	Polar	Total Score	Crash (-Ve)	Polar	Total Score	Crash (−Ve)	Polar	Total Score	Crash (−Ve)	Polar	Total Score	Crash (−Ve)	Polar
HIM1	5.097	− 4.375	0.114	4.258	− 1.555	1.784	4.904	− 0.870	0.004	5.794	− 0.514	3.828	5.770	− 1.440	2.231
HIM2	4.057	− 6.172	0.039	4.624	− 0.997	1.959	4.906	− 0.468	1.517	5.052	− 0.780	3.415	5.414	− 1.529	2.444
HIM3	5.353	− 5.309	0.161	4.578	− 1.492	1.790	5.042	− 1.845	1.073	5.680	− 0.804	3.956	5.350	− 1.170	2.316
HIM4	3.976	− 5.132	0.167	3.839	− 1.635	1.196	6.328	− 1.172	1.128	4.966	− 0.666	1.818	5.627	− 1.416	2.320
HIM5	2.707	− 7.759	0.179	4.067	− 0.997	1.987	5.254	− 0.674	1.618	4.563	− 1.068	2.935	5.698	− 1.240	2.485
HIM6	5.948	− 3.902	1.796	5.229	− 0.707	3.656	6.766	− 1.424	1.858	6.670	− 0.941	5.519	6.218	− 1.468	2.578
HIM7	5.019	− 7.055	0.203	4.382	− 2.443	3.329	6.028	− 0.629	1.660	5.374	− 1.876	0.670	6.627	− 1.484	2.579
HIM8	4.429	− 3.983	0.344	4.150	− 4.382	4.210	6.794	− 1.279	1.129	5.468	− 0.769	3.444	5.842	− 2.088	2.272
HIM9	3.722	− 5.956	0.197	4.051	− 2.006	0.002	6.116	− 0.597	1.717	5.407	− 0.565	1.370	5.877	− 2.054	0.792
HIM10	6.771	− 3.977	1.836	5.176	− 3.512	4.023	5.332	− 1.378	3.349	6.071	− 0.923	3.854	5.825	− 0.966	3.672
HIM11	3.775	− 6.079	0.898	6.998	− 2.086	3.842	8.678	− 1.065	2.876	5.087	− 0.881	1.854	5.948	− 1.015	1.202
HIM12	0.190	− 8.149	0.022	5.302	− 2.305	3.475	5.227	− 1.636	0.710	7.322	− 1.128	4.099	6.237	− 2.562	2.171
HIM13	4.523	− 4.537	0.014	4.840	− 0.664	3.305	6.181	− 2.966	3.523	5.281	− 0.503	3.981	5.905	− 1.136	2.218
HIM14	5.247	− 3.183	0.000	4.888	− 1.296	2.563	5.037	− 0.377	1.647	7.057	− 0.799	4.256	6.116	− 1.366	2.438
HIM15	4.633	− 4.173	0.180	3.756	− 0.710	2.072	5.188	− 1.559	1.143	5.570	− 1.125	3.718	6.238	− 1.708	2.443
HIM16	4.588	− 2.883	0.000	6.027	− 1.099	3.903	5.606	− 0.987	4.197	5.661	− 0.926	2.751	7.263	− 1.533	4.212
HIM17	3.944	− 4.806	0.236	5.329	− 0.590	2.798	4.830	− 1.682	1.617	6.234	− 0.830	3.912	5.366	− 1.257	3.022
HTZ1	0.593	− 7.518	0	4.221	− 0.692	1.975	3.993	− 0.539	0.003	5.284	− 0.566	1.432	5.227	− 1.045	0.903
HTZ2	− 1.770	− 8.477	0.000	4.055	− 1.438	2.877	4.388	− 1.665	1.170	4.100	− 0.546	3.017	4.563	− 0.976	1.266
HTZ3	4.649	− 5.870	0.148	5.104	− 0.861	3.922	4.243	− 1.539	1.933	5.304	− 1.370	1.398	5.138	− 1.217	1.930
HTZ4	− 3.169	− 12.002	0.482	4.173	− 1.898	1.864	4.654	− 1.627	1.128	5.163	− 0.745	1.304	5.084	− 1.143	1.018
HTZ5	4.021	− 12.325	0.246	3.215	− 1.481	4.232	3.256	− 6.374	2.317	2.382	− 5.263	1.238	4.623	− 0.951	1.280
HTZ6	6.605	− 3.866	1.6487	4.004	− 1.104	2.851	5.834	− 1.310	3.111	5.286	− 1.530	1.436	6.336	− 2.326	3.781
HTZ7	4.977	− 5.434	0.655	5.197	− 2.040	3.250	5.352	− 1.371	1.172	6.138	− 1.734	1.627	4.908	− 1.057	1.335
HTZ8	1.025	− 8.223	0.000	3.549	− 1.310	2.403	4.024	− 3.825	2.440	4.980	− 0.593	1.474	5.164	− 1.291	0.999
HTZ9	3.386	− 7.041	0.194	5.567	− 1.622	3.057	6.792	− 2.581	1.088	5.794	− 0.683	1.774	6.053	− 1.408	1.037
HTZ10	4.744	− 5.463	0.837	4.520	− 2.469	3	7.758	− 1.518	3.765	5.345	− 1.133	3.661	7.507	− 2.080	4.086
HTZ11	2.991	− 6.177	0	3.453	− 0.721	1.266	4.841	− 1.738	0.045	4.368	− 0.805	1.074	6.176	− 1.380	1.511
HTZ12	4.810	− 6.157	0.275	5.296	− 2.814	3.605	5.138	− 1.840	2.189	5.083	− 0.870	1.536	5.592	− 1.321	1.525
HTZ13	4.868	− 3.837	0	4.863	− 0.535	2.405	4.656	− 0.681	3.152	4.246	− 2.335	0.529	6.404	− 0.975	2.954
HTZ14	5.646	− 3.473	0	4.948	− 0.801	2.324	4.953	− 1.672	1.066	5.058	− 1.174	1.121	5.114	− 1.299	1.296
HTZ15	3.428	− 4.957	0.348	3.949	− 0.614	1.873	4.049	− 0.787	1.224	4.796	− 1.066	1.489	3.510	− 0.607	0.461
HTZ16	4.227	− 4.787	0.298	4.654	− 1.534	2.096	6.143	− 1.204	2.879	4.854	− 0.994	1.564	7.102	− 0.917	3.001
HTZ17	3.784	− 5.018	0.380	3.661	− 0.897	1.676	4.016	− 1.179	1.384	4.039	− 0.569	2.706	4.256	− 1.040	1.236
HTZ STD	4.722	− 1.084	1.063	3.319	− 0.890	3.033	3.564	− 0.272	2.367	3.394	− 0.882	1.169	4.237	− 0.801	1.855

## Discussion

HF is the most prevalent form of cardiovascular disease among the elderly. A complete studies of HF, comprising pathogenic factors, pathological processes, clinical manifestations, early clinical diagnosis, clinical prevention, and drug therapy targets urgency to be consistently analyzed. In the present investigation, bioinformatics analysis was engaged to explore HF biomarkers and the pathological processes in myocardial tissues, acquired from HF groups and non heart failure groups. We analyzed GSE141910 expression profiling by high throughput sequencing obtained 881 different genes between HF groups and non heart failure groups, 442 up regulated and 439 down regulated genes. HBA2 and HBA1 have a key role in hypertension [41], but these genes might be linked with development HF. SFRP4 was linked with progression of myocardial ischemia [42]. Emmens et al. [43] and Broch et al. [44] found that PENK (proenkephalin) and IL1RL1 were up regulated in HF. ALOX15B has lipid accumulation and inflammation activity and is highly expressed in atherosclerosis [45]. Studies have shown that expression of MYH6 was associated with hypertrophic cardiomyopathy [46].

In functional enrichment analysis, some genes involved with regulation of cardiovascular system processes were enriched in HF. Liu et al. [47], Kosugi et al. [48], McMacken et al. [49], Pan and Zhang [50], Li et al. [51] and Jiang et al. [52] presented that expression of HLA-DQA1, KDM5D, UCHL1, SAA1, ARG1 and LYVE1 were associated with progression of cardiomyopathy. Hou et al. [53] and Olesen et al. [54] demonstrated that DACT2 and KCND3 were found to be substantially related to atrial fibrillation. Ge and Concannon [55], Ferjeni et al. [56], Anquetil et al. [57], Glawe et al. [58], Kawabata et al. [59], Li et al. [60], Buraczynska et al. [61], Amini et al. [62], Yang et al. [63], Du Toit et al. [64], Hirose et al. [65], Zhang et al. [66], Griffin et al. [67], Zouidi et al. [68], Trombetta et al. [69], Alharbi et al. [70], Ikarashi et al. [71], Dharmadhikari et al. [72], Sutton et al. [73] and Deng et al. [74] reported that UBASH3A, ZAP70, IDO1, ITGAL (integrin subunit alpha L). ITGB7, RASGRP1, CNR1, SLC2A1, SLC11A1, GPR84, SSTR5, KCNB1, GLUL (glutamate-ammonia ligase), BANK1, CACNA1E, LGR5, AQP3, SIGLEC7, SSTR2 and DNER (delta/notch like EGF repeat containing) could be an index for diabetes, but these genes might be responsible for progression of HF. Experiments show that expression of FAP (fibroblast activation protein alpha) [75], THBS4 [76], CD27 [77], LEF1 [78], CTHRC1 [79], ESR1 [80], CXCL9 [81], SERPINA3 [82], TRPC4 [83], F13A1 [84], PIK3C2A [85], KCNIP2 [86] and GPR4 [87] contributed to myocardial infarction. MFAP4 [88], ALOX15 [89], COL1A1 [90], APOA1 [91], PDE5A [92], CX3CR1 [93], THY1 [94], GREM1 [95], FMOD (fibromodulin) [96], NPPA (natriuretic peptide A) [97], LTBP2 [98], LUM (lumican) [99], IL34 [100], NRG1 [101], CXCL14 [102], CXCL10 [103], ACE (angiotensin I converting enzyme) [104], CFTR (ystic fibrosis transmembrane conductance regulator) [105], S100A8 [106], S100A9 [106], HP (haptoglobin) [107], AGTR1 [108], ATP2A2 [109], IL10 [110], EDN1 [111], TLR2 [112], MCEMP1 [113], TPO (thyroid peroxidase) [114], CD163 [115], IL18R1 [116], KCNA7 [117] and CALCRL (calcitonin receptor like receptor) [118] have an important role in HF. Li et al. [119], Deckx et al. [120], Ichihara et al. [121] and Paik et al. [122] showed that the SERPINE2, OGN (osteoglycin), AGTR2 and WNT10B promoted cardiac interstitial fibrosis. Cai et al. [123], Mo et al. [124], Sun et al. [125], Martinelli et al. [126], Zhao et al. [127], Assimes et al. [128] and Piechota et al. [129] showed that CCR7, FCN1, ESM1, F8 (coagulation factor VIII), C1QTNF1, ALOX5 and MSR1 were an important target gene for coronary artery disease. STAB2 have been suggested to be associated with venous thromboembolic disease [130]. Genes such as COMP (cartilage oligomeric matrix protein) [131], CHI3L1 [132], PLA2G2A [133], P2RY12 [134], CR1 [135], HPSE (heparanase) [136], PTX3 [137] and SERPINE1 [138] were related to atherosclerosis. CCDC80 [139], CMA1 [140], MDK (midkine) [141], GNA14 [142], SCG2 [143], NPPB (natriuretic peptide B) [144], FGF10 [145], ARNTL (aryl hydrocarbon receptor nuclear translocator like) [146], WNK3 [147], EDNRB (endothelin receptor type B) [148], THBS1 [149], SELE (selectin E) [150], SLC4A7 [151], AQP4 [152] and KCNK3 [153] are thought to be responsible for progression of hypertension, but these genes might to be associated with progression of HF. CNTNAP2 [154], GLI2 [155], DPT (dermatopontin) [156], AEBP1 [157], ITIH5 [158], CXCL11 [159], GDNF (glial cell derived neurotrophic factor) [160], MCHR1 [161], FLT3 [162], ELANE (elastase, neutrophil expressed) [163], OSMR (oncostatin M receptor) [164] and IL15RA [165] are involved in development of obesity, but these genes might be key for progression of HF. CTSG (cathepsin G) is a protein coding gene plays important roles in aortic aneurysms [166]. Evidence from Safa et al. [167], Chen et al. [168], Zhou et al. [169], Hu et al. [170], Lou et al. [171], Zhang et al. [172] and Chen et al. [173] study indicated that the expression of CCL22, CCR1, FPR1, KNG1, CRISPLD2, CD38 and GPRC5A were linked with progression of ischemic heart disease. Li et al. [174] showed that STEAP3 expression can be associated with cardiac hypertrophy progression.

The HiPPIE interactome database was used to construct the PPI network, and modules analysis was performed. We finally screened out up regulated hub genes and down regulated hub genes, including ESR1, PYHIN1, PPP2R2B, LCK, TP63, PCLAF, CD247, CD2, CD5, CD48, CFTR, TK1, ECT2, FKBP5, S100A9 and S100A8 from the PPI network and its modules. TP63 might serve as a potential prognostic factor in cardiomyopathy [175]. The expression of FKBP5 is related to the progression of coronary artery disease [176]. CD247 plays a central role in hypertension [177], but this gene might be involved in the HF. PYHIN1, PPP2R2B, LCK (LCK proto-oncogene, Src family tyrosine kinase), PCLAF (PCNA clamp associated factor), TK1, ECT2, CD2, CD5 and CD48 might be the novel biomarker for HF.

The miRNet database and NetworkAnalyst database were used to construct the target gene—miRNA regulatory network and target gene—TF regulatory network. We finally screened out target genes, miRNA, TFs, including FSCN1, ESR1, TMEM30B, SCN2B, CENPA, FKBP5, PCLAF, CEP55, ATP2A2, TK1, APOA1, COL1A1, HBB, LCK, SOCS3, BCL6, ANLN, hsa-mir-4533, hsa-mir-548ac, hsa-mir-548i, hsa-mir-5585-3p, hsa-mir-6750-3p, hsa-mir-200c-3p, hsa-mir-1273 g-3p, hsa-mir-1244, hsa-mir-4789-3p, hsa-mir-766-3p, ESRRA, RERE, HMG20B, THRAP3, ATF1, MXD3, ARID4B, CBFB, TAF7 and CREM from the target gene—miRNA regulatory network and target gene—TF regulatory network. SCN2B [178] and SOCS3 [179] are considered as a markers for HF and might be a new therapeutic target. BCL6 levels are correlated with disease severity in patients with atherosclerosis [180]. A previous study showed that hsa-mir-1273 g-3p [181], hsa-mir-4789-3p [182] and ATF1 [183] could involved in hypertension, but these markers might be responsible for progression of HF. hsa-miR-518f, was demonstrated to be associated with cardiomyopathy [184]. An evidence demonstrating a role for ESRRA (estrogen related receptor alpha) [185] and THRAP3 [186] in diabetes, but these genes might be liable for development of HF. FSCN1, TMEM30B, CENPA (centromere protein A), CEP55, HBB (hemoglobin subunit beta), ANLN (anillin actin binding protein), hsa-mir-4533, hsa-mir-548ac, hsa-mir-548i, hsa-mir-5585-3p, hsa-mir-6750-3p, hsa-mir-200c-3p, hsa-mir-1244, RERE(arginine-glutamic acid dipeptide repeats), HMG20B, MXD3, ARID4B, CBFB (core-binding factor subunit beta), TAF7 and CREM (cAMP response element modulator) might be the novel biomarker for HF.

**Fig. 9:**
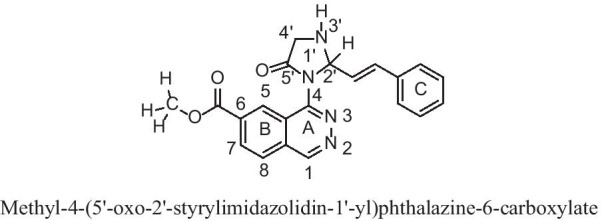
Structure of active designed molecule of HIM11

**Fig.10:**
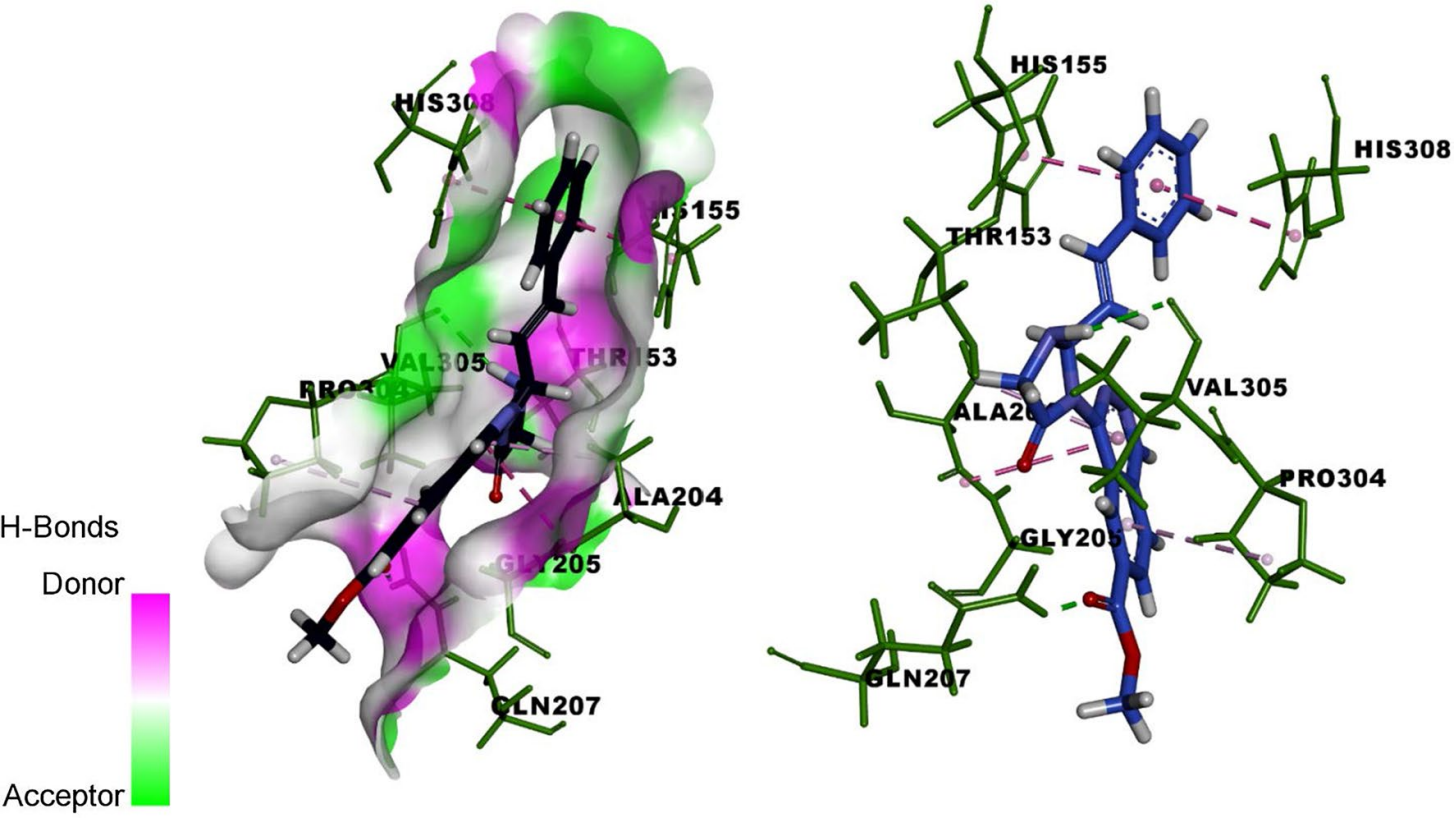
3D binding of molecule HIM11 with 2HV7

**Fig.11:**
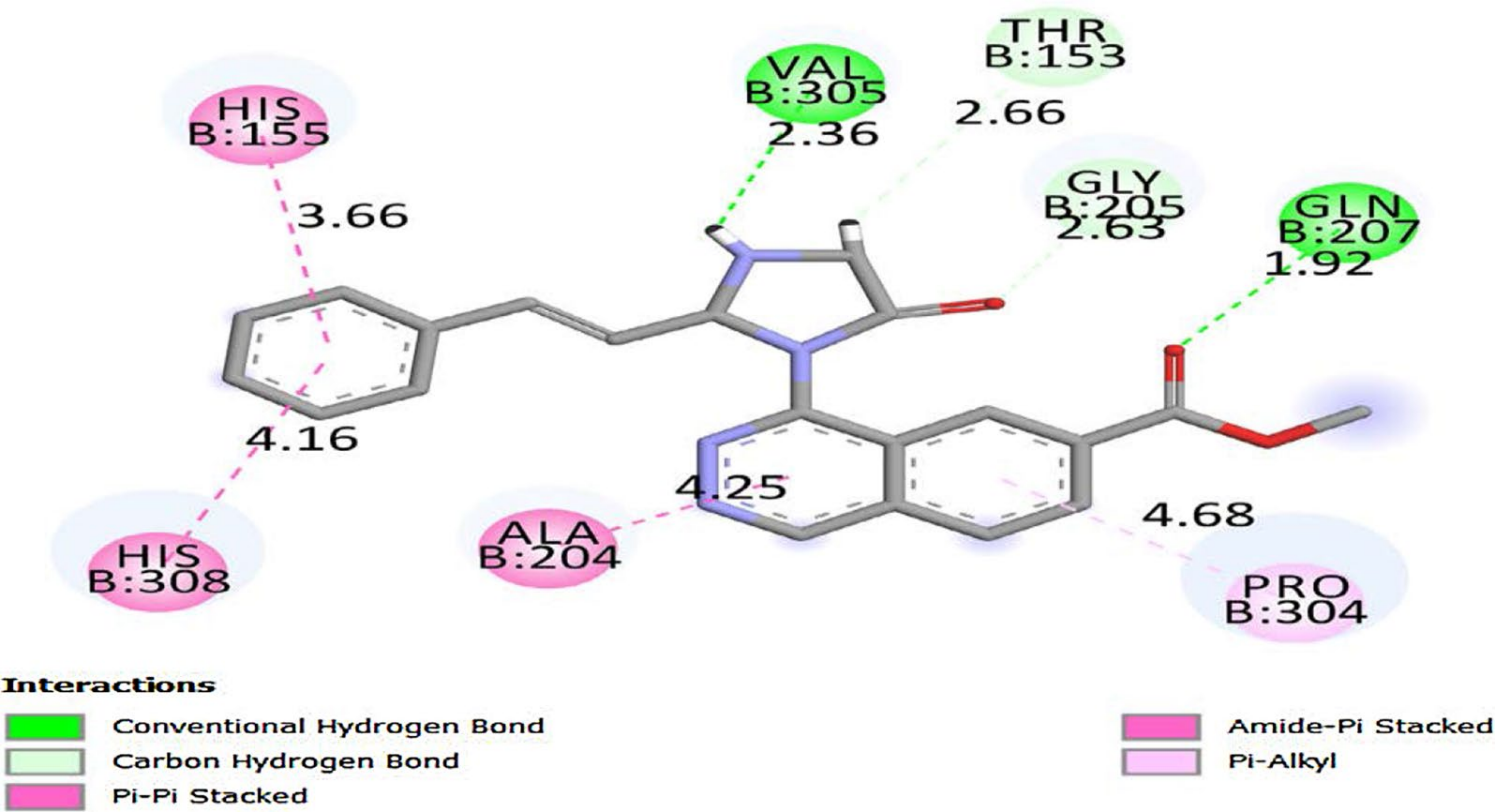
2D binding of molecule HIM11 with 2HV7

The molecules HIM6, HIM10 obtained good binding score of more 5 to 6.999 with all proteins and the molecules HIM11, HIM12, HIM14, HTZ9, HTZ10 and HTZ12 obtained binding score above 5 and less than 9 with PDB protein code of 2HV7, 3VD8 and 6RUR respectively. The molecule HIM11 obtained highest binding score of 8.678 with 2HV7 and its interaction with amino acids are molecule HIM11 (Fig. 9) has obtained with a high binding score with PDB protein 2HV7, the interactions of molecule is the C6 side chin acyl carbonyl C=O formed hydrogen bond interaction with amino acid GLN-207 with bond length 1.92 A° and 3’ N–H group of imidazole ring formed hydrogen bond interaction with VAL-305 with bond length 2.36 A° respectively. It also formed other interactions of carbon hydrogen bond of –CH_3_ group of carboxylate at C6 with PRO-304 and amide-pi stacked and pi–pi stacked interaction of electrons of aromatic ring A with ALA-204 and ring C with HIS-155 and HIS-308. Molecule formed pi-alkyl interaction of ring B with PRO-304 and all interactions with amino acids and bond length are depicted by 3D and 2D figures (Fig. 10 and Fig. 11).

## Conclusions

The present investigation aimed at characterizing the expression profiling by high throughput sequencing of the HF patients. Our bioinformatics analyses revealed key gene signatures as candidate biomarkers in HF. Hub genes (ESR1, PYHIN1, PPP2R2B, LCK, TP63, PCLAF, CFTR, TK1, ECT2 and FKBP5) were diagnosed as an essential genetic factors in HF. In general, DEGs linked with HF genes, including already known markers of HF and other HF related diseases, and novel biomarkers, were diagnosed. Potential implicated miRNAs and TFs were also diagnosed. The diagnosed hub genes might represent candidate diagnostic and prognostic biomarkers, and therapeutic targets. The current investigation reported novel genes and signaling pathways in HF, and further investigation is required.

## Supplementary Information

Additional file 1: Table S1. The statistical metrics for key differentially expressed genes (DEGs).

## Data Availability

The datasets supporting the conclusions of this article are available in the GEO (Gene Expression Omnibus) (https://www.ncbi.nlm.nih.gov/geo/) repository. [(GSE141910) (https://www.ncbi.nlm.nih.gov/geo/query/acc.cgi?acc=GSE141910].
