# Supplementary material for: Identification of candidate biomarkers and therapeutic agents for heart failure by bioinformatics analysis
Source: BMC Cardiovasc Disord. 2021 Jul 4;21:329. doi: 10.1186/s12872-021-02146-8 (PMC8256614; doi:10.1186/s12872-021-02146-8)
Supplement: Supplementary file 1 — The statistical metrics for key differentially expressed genes (DEGs). [file 12872_2021_2146_MOESM1_ESM.docx]

**Table S1** The statistical metrics for key differentially expressed genes (DEGs)

| **GeneSymbol** | **logFC** | **pValue** | **adj.P.Val** | **tvalue** | **Regulation** | **Gene Name** |
| --- | --- | --- | --- | --- | --- | --- |
| SEZ6L | 3.540921 | 3.57E-88 | 1.24E-84 | 26.74214 | Up | seizure related 6 homolog like |
| HBA2 | 3.52441 | 2.69E-34 | 1.06E-32 | 13.57556 | Up | hemoglobin subunit alpha 2 |
| FHAD1-AS1 | 3.514827 | 9.64E-90 | 4.01E-86 | 27.14088 | Up | FHAD1 antisense RNA 1 |
| COL22A1 | 3.442684 | 3.27E-74 | 4E-71 | 23.26846 | Up | collagen type XXII alpha 1 chain |
| SFRP4 | 3.439595 | 3.23E-87 | 9.58E-84 | 26.49986 | Up | secreted frizzled related protein 4 |
| HBB | 3.311845 | 4.49E-46 | 4.34E-44 | 16.48352 | Up | hemoglobin subunit beta |
| PENK | 3.292908 | 1.23E-69 | 8.22E-67 | 22.15479 | Up | proenkephalin |
| HBA1 | 3.239241 | 7.91E-35 | 3.22E-33 | 13.71027 | Up | hemoglobin subunit alpha 1 |
| APCDD1L | 3.102854 | 1.43E-70 | 9.91E-68 | 22.38121 | Up | APC down-regulated 1 like |
| FNDC1 | 2.952 | 8.2E-99 | 8.53E-95 | 29.4879 | Up | fibronectin type III domain containing 1 |
| SLC1A7 | 2.935446 | 3.45E-58 | 9.31E-56 | 19.39718 | Up | solute carrier family 1 member 7 |
| LYPD1 | 2.786956 | 1.94E-49 | 2.57E-47 | 17.29603 | Up | LY6/PLAUR domain containing 1 |
| FCER1A | 2.775152 | 5.58E-63 | 2.11E-60 | 20.5475 | Up | Fc fragment of IgE receptor Ia |
| HAPLN1 | 2.766834 | 6.66E-48 | 7.6E-46 | 16.92589 | Up | hyaluronan and proteoglycan link protein 1 |
| LEFTY2 | 2.748568 | 3.27E-71 | 2.52E-68 | 22.53707 | Up | left-right determination factor 2 |
| CAPN6 | 2.718519 | 3.14E-44 | 2.57E-42 | 16.03574 | Up | calpain 6 |
| UNC80 | 2.706223 | 6.44E-53 | 1.21E-50 | 18.13271 | Up | unc-80 homolog, NALCN channel complex subunit |
| CHRNA3 | 2.691289 | 3.77E-48 | 4.53E-46 | 16.98549 | Up | cholinergic receptor nicotinic alpha 3 subunit |
| ARMS2 | 2.677012 | 7.71E-60 | 2.39E-57 | 19.79325 | Up | age-related maculopathy susceptibility 2 |
| MXRA5 | 2.665146 | 7.01E-66 | 3.31E-63 | 21.24615 | Up | matrix remodeling associated 5 |
| CXCL10 | 2.648986 | 1.88E-39 | 1.06E-37 | 14.8648 | Up | C-X-C motif chemokine ligand 10 |
| LAMP5 | 2.634422 | 1.79E-67 | 8.84E-65 | 21.63104 | Up | lysosomal associated membrane protein family member 5 |
| GZMH | 2.585101 | 1.39E-52 | 2.54E-50 | 18.05238 | Up | granzyme H |
| COL10A1 | 2.58157 | 3.89E-42 | 2.71E-40 | 15.52492 | Up | collagen type X alpha 1 chain |
| TNMD | 2.561494 | 2.35E-41 | 1.55E-39 | 15.3336 | Up | tenomodulin |
| CRISPLD1 | 2.551697 | 1.75E-57 | 4.48E-55 | 19.22821 | Up | cysteine rich secretory protein LCCL domain containing 1 |
| MKRN2OS | 2.518052 | 1.81E-31 | 5.81E-30 | 12.85257 | Up | MKRN2 opposite strand |
| CMA1 | 2.49186 | 2.56E-41 | 1.68E-39 | 15.32454 | Up | chymase 1 |
| CRACD | 2.409728 | 7.61E-35 | 3.1E-33 | 13.71447 | Up | capping protein inhibiting regulator of actin dynamics |
| LOC339260 | 2.40851 | 9.48E-72 | 8.21E-69 | 22.66783 | Up | uncharacterized LOC339260 |
| CENPA | 2.378802 | 2.87E-29 | 7.86E-28 | 12.27893 | Up | centromere protein A |
| LINC02593 | 2.357621 | 6.61E-52 | 1.09E-49 | 17.8899 | Up | long intergenic non-protein coding RNA 2593 |
| LRRC55 | 2.35454 | 4.34E-43 | 3.3E-41 | 15.75782 | Up | leucine rich repeat containing 55 |
| AGTR2 | 2.346789 | 3.04E-39 | 1.69E-37 | 14.81336 | Up | angiotensin II receptor type 2 |
| GLIS1 | 2.270732 | 1.08E-41 | 7.24E-40 | 15.41668 | Up | GLIS family zinc finger 1 |
| AQP10 | 2.238434 | 5.77E-60 | 1.82E-57 | 19.82339 | Up | aquaporin 10 |
| TMEM130 | 2.225534 | 1.08E-32 | 3.78E-31 | 13.16693 | Up | transmembrane protein 130 |
| CXCL11 | 2.22214 | 3.56E-30 | 1.03E-28 | 12.51639 | Up | C-X-C motif chemokine ligand 11 |
| NKG7 | 2.203903 | 2.69E-41 | 1.75E-39 | 15.3192 | Up | natural killer cell granule protein 7 |
| ESM1 | 2.203875 | 1.56E-35 | 6.67E-34 | 13.88776 | Up | endothelial cell specific molecule 1 |
| NPPA | 2.194101 | 5.13E-29 | 1.38E-27 | 12.21264 | Up | natriuretic peptide A |
| FRZB | 2.175346 | 4.1E-91 | 2.13E-87 | 27.49106 | Up | frizzled related protein |
| SCUBE2 | 2.170552 | 1.48E-78 | 2.8E-75 | 24.33596 | Up | signal peptide, CUB domain and EGF like domain containing 2 |
| TGFB2-AS1 | 2.167994 | 1.32E-41 | 8.84E-40 | 15.39479 | Up | TGFB2 antisense RNA 1 (head to head) |
| ANKRD34C | 2.159555 | 2.72E-45 | 2.48E-43 | 16.29382 | Up | ankyrin repeat domain 34C |
| ZNF365 | 2.146095 | 9.54E-49 | 1.2E-46 | 17.1295 | Up | zinc finger protein 365 |
| KCNIP1 | 2.140052 | 9.06E-50 | 1.24E-47 | 17.37589 | Up | potassium voltage-gated channel interacting protein 1 |
| CD1C | 2.134028 | 3.98E-44 | 3.25E-42 | 16.01054 | Up | CD1c molecule |
| ASPN | 2.117196 | 3.21E-68 | 1.71E-65 | 21.8113 | Up | asporin |
| PPP2R2B | 2.092908 | 2.1E-59 | 6.14E-57 | 19.68888 | Up | protein phosphatase 2 regulatory subunit Bbeta |
| NPPB | 2.091998 | 1.57E-18 | 1.74E-17 | 9.274014 | Up | natriuretic peptide B |
| HRK | 2.091711 | 4.32E-44 | 3.5E-42 | 16.00206 | Up | harakiri, BCL2 interacting protein |
| LOC101928834 | 2.091398 | 4.58E-47 | 4.96E-45 | 16.72358 | Up | uncharacterized LOC101928834 |
| GZMB | 2.085361 | 2.05E-40 | 1.25E-38 | 15.10213 | Up | granzyme B |
| RTL3 | 2.083673 | 1.52E-53 | 3.09E-51 | 18.28344 | Up | retrotransposon Gag like 3 |
| CX3CR1 | 2.069035 | 3.8E-45 | 3.42E-43 | 16.25861 | Up | C-X3-C motif chemokine receptor 1 |
| COMP | 2.051651 | 1.51E-19 | 1.82E-18 | 9.579662 | Up | cartilage oligomeric matrix protein |
| CTSG | 2.049972 | 5.94E-37 | 2.83E-35 | 14.24398 | Up | cathepsin G |
| FAM180B | 2.034044 | 4.11E-41 | 2.64E-39 | 15.27385 | Up | family with sequence similarity 180 member B |
| MDK | 2.023427 | 5.33E-46 | 5.08E-44 | 16.46554 | Up | midkine |
| NFIA-AS2 | 2.011983 | 1.41E-51 | 2.25E-49 | 17.81082 | Up | NFIA antisense RNA 2 |
| FATE1 | 2.011836 | 5.31E-38 | 2.71E-36 | 14.50539 | Up | fetal and adult testis expressed 1 |
| PRKAG3 | 2.002215 | 4.44E-48 | 5.27E-46 | 16.96846 | Up | protein kinase AMP-activated non-catalytic subunit gamma 3 |
| COL9A1 | 2.001739 | 5.6E-52 | 9.31E-50 | 17.90721 | Up | collagen type IX alpha 1 chain |
| SSC5D | 1.998905 | 6.1E-72 | 5.51E-69 | 22.71447 | Up | scavenger receptor cysteine rich family member with 5 domains |
| ACE | 1.99576 | 1.54E-53 | 3.12E-51 | 18.28159 | Up | angiotensin I converting enzyme |
| GDF6 | 1.994884 | 6.98E-50 | 9.74E-48 | 17.40311 | Up | growth differentiation factor 6 |
| SHISA2 | 1.989108 | 1.04E-43 | 8.27E-42 | 15.90886 | Up | shisa family member 2 |
| XG | 1.986441 | 2.84E-34 | 1.11E-32 | 13.56967 | Up | Xg glycoprotein (Xg blood group) |
| LUM | 1.96673 | 4.91E-75 | 6.81E-72 | 23.47008 | Up | lumican |
| RGS4 | 1.958569 | 2.81E-47 | 3.09E-45 | 16.77466 | Up | regulator of G protein signaling 4 |
| FCGR3B | 1.945296 | 1.19E-31 | 3.87E-30 | 12.89978 | Up | Fc fragment of IgG receptor IIIb |
| HSH2D | 1.94243 | 6.51E-46 | 6.15E-44 | 16.44441 | Up | hematopoietic SH2 domain containing |
| WNT9A | 1.941347 | 4.74E-36 | 2.09E-34 | 14.018 | Up | Wnt family member 9A |
| SAMD11 | 1.937898 | 2.66E-28 | 6.76E-27 | 12.02359 | Up | sterile alpha motif domain containing 11 |
| LTB | 1.919135 | 1.96E-30 | 5.77E-29 | 12.5841 | Up | lymphotoxin beta |
| CTSW | 1.916821 | 3.18E-37 | 1.55E-35 | 14.31185 | Up | cathepsin W |
| ARMC4 | 1.913783 | 2.91E-50 | 4.17E-48 | 17.49459 | Up | armadillo repeat containing 4 |
| ATP1B4 | 1.909761 | 8.05E-19 | 9.12E-18 | 9.36208 | Up | ATPase Na+/K+ transporting family member beta 4 |
| TNNI1 | 1.901808 | 5.89E-21 | 8.11E-20 | 9.993524 | Up | troponin I1, slow skeletal type |
| CYP4F3 | 1.901148 | 4.61E-32 | 1.55E-30 | 13.0053 | Up | cytochrome P450 family 4 subfamily F member 3 |
| RYR3 | 1.890853 | 2.04E-46 | 2.07E-44 | 16.56646 | Up | ryanodine receptor 3 |
| NDP | 1.884805 | 7.22E-39 | 3.91E-37 | 14.72038 | Up | norrincystine knot growth factor NDP |
| SLAMF7 | 1.880253 | 9.55E-37 | 4.48E-35 | 14.1924 | Up | SLAM family member 7 |
| PI16 | 1.873783 | 2.52E-45 | 2.31E-43 | 16.30173 | Up | peptidase inhibitor 16 |
| OGN | 1.873487 | 6.47E-51 | 9.81E-49 | 17.65171 | Up | osteoglycin |
| BIRC7 | 1.867331 | 1.22E-20 | 1.61E-19 | 9.901493 | Up | baculoviral IAP repeat containing 7 |
| APLNR | 1.85994 | 8.58E-53 | 1.59E-50 | 18.10284 | Up | apelin receptor |
| ADAM18 | 1.857904 | 1.11E-25 | 2.24E-24 | 11.31964 | Up | ADAM metallopeptidase domain 18 |
| OASL | 1.857886 | 9.34E-50 | 1.27E-47 | 17.3727 | Up | 2'-5'-oligoadenylate synthetase like |
| CREG2 | 1.844542 | 3.04E-37 | 1.49E-35 | 14.31649 | Up | cellular repressor of E1A stimulated genes 2 |
| SIGLEC17P | 1.840393 | 6.23E-38 | 3.17E-36 | 14.48798 | Up | sialic acid binding Ig like lectin 17, pseudogene |
| LINC00861 | 1.837563 | 1.09E-39 | 6.29E-38 | 14.92381 | Up | long intergenic non-protein coding RNA 861 |
| GPR68 | 1.834869 | 2.31E-32 | 7.86E-31 | 13.0825 | Up | G protein-coupled receptor 68 |
| ISLR | 1.831354 | 1.03E-49 | 1.39E-47 | 17.36275 | Up | immunoglobulin superfamily containing leucine rich repeat |
| AK5 | 1.822757 | 3.92E-60 | 1.25E-57 | 19.8637 | Up | adenylate kinase 5 |
| FREM1 | 1.822486 | 3.96E-86 | 1.03E-82 | 26.22491 | Up | FRAS1 related extracellular matrix 1 |
| SYTL5 | 1.818907 | 5.97E-20 | 7.48E-19 | 9.698984 | Up | synaptotagmin like 5 |
| STAT4 | 1.815386 | 1.94E-40 | 1.18E-38 | 15.10812 | Up | signal transducer and activator of transcription 4 |
| SMOC2 | 1.808707 | 3.5E-105 | 7.3E-101 | 31.18302 | Up | SPARC related modular calcium binding 2 |
| SAMD3 | 1.804067 | 4.45E-45 | 3.97E-43 | 16.2419 | Up | sterile alpha motif domain containing 3 |
| VGLL2 | 1.802895 | 5.36E-18 | 5.67E-17 | 9.111967 | Up | vestigial like family member 2 |
| LINC01563 | 1.792042 | 1.87E-40 | 1.15E-38 | 15.11196 | Up | long intergenic non-protein coding RNA 1563 |
| KCNK17 | 1.791993 | 4.93E-29 | 1.32E-27 | 12.21731 | Up | potassium two pore domain channel subfamily K member 17 |
| TMEM119 | 1.786136 | 3.08E-34 | 1.2E-32 | 13.5608 | Up | transmembrane protein 119 |
| SLC16A9 | 1.78355 | 4.41E-59 | 1.24E-56 | 19.61146 | Up | solute carrier family 16 member 9 |
| GZMA | 1.780273 | 7.61E-43 | 5.69E-41 | 15.69824 | Up | granzyme A |
| OGDHL | 1.772273 | 6.58E-45 | 5.8E-43 | 16.20069 | Up | oxoglutarate dehydrogenase like |
| ATP1A4 | 1.772266 | 1.92E-44 | 1.6E-42 | 16.08796 | Up | ATPase Na+/K+ transporting subunit alpha 4 |
| GAP43 | 1.770722 | 1.3E-26 | 2.84E-25 | 11.57199 | Up | growth associated protein 43 |
| RASEF | 1.767816 | 9.28E-35 | 3.77E-33 | 13.69272 | Up | RAS and EF-hand domain containing |
| CHD5 | 1.767757 | 3.25E-36 | 1.46E-34 | 14.05911 | Up | chromodomain helicase DNA binding protein 5 |
| PRF1 | 1.765387 | 2.68E-42 | 1.88E-40 | 15.56459 | Up | perforin 1 |
| OXER1 | 1.757588 | 1.14E-51 | 1.86E-49 | 17.8326 | Up | oxoeicosanoid receptor 1 |
| LSP1 | 1.755261 | 7.18E-55 | 1.57E-52 | 18.6014 | Up | lymphocyte specific protein 1 |
| NRXN2 | 1.753031 | 1.35E-39 | 7.71E-38 | 14.90069 | Up | neurexin 2 |
| CXCL9 | 1.752192 | 1.37E-20 | 1.81E-19 | 9.886423 | Up | C-X-C motif chemokine ligand 9 |
| MYL1 | 1.741306 | 3.26E-23 | 5.46E-22 | 10.6376 | Up | myosin light chain 1 |
| DBH-AS1 | 1.740319 | 2.27E-43 | 1.76E-41 | 15.82628 | Up | DBH antisense RNA 1 |
| CERS1 | 1.737417 | 7.6E-28 | 1.84E-26 | 11.90259 | Up | ceramide synthase 1 |
| OPCML | 1.7359 | 8.75E-40 | 5.11E-38 | 14.94691 | Up | opioid binding protein/cell adhesion molecule like |
| APOA1 | 1.735578 | 1.38E-27 | 3.24E-26 | 11.83363 | Up | apolipoprotein A1 |
| STAB2 | 1.73447 | 1.05E-26 | 2.31E-25 | 11.5969 | Up | stabilin 2 |
| C16orf89 | 1.731539 | 1.26E-40 | 7.83E-39 | 15.15416 | Up | chromosome 16 open reading frame 89 |
| LRRC15 | 1.731261 | 5.75E-30 | 1.65E-28 | 12.46207 | Up | leucine rich repeat containing 15 |
| C1QTNF7 | 1.725627 | 7.28E-71 | 5.4E-68 | 22.45257 | Up | C1q and TNF related 7 |
| TMEM200A | 1.7209 | 4.9E-25 | 9.41E-24 | 11.14357 | Up | transmembrane protein 200A |
| LHCGR | 1.716273 | 2.18E-22 | 3.38E-21 | 10.40422 | Up | luteinizing hormone/choriogonadotropin receptor |
| PRSS35 | 1.713597 | 7.99E-34 | 3.01E-32 | 13.45573 | Up | serine protease 35 |
| S1PR5 | 1.710201 | 5.13E-30 | 1.48E-28 | 12.47513 | Up | sphingosine-1-phosphate receptor 5 |
| NTM | 1.707182 | 4.2E-65 | 1.94E-62 | 21.05873 | Up | neurotrimin |
| FCRL3 | 1.703236 | 3.65E-29 | 9.88E-28 | 12.25172 | Up | Fc receptor like 3 |
| KLHDC8A | 1.697472 | 3.11E-36 | 1.4E-34 | 14.06389 | Up | kelch domain containing 8A |
| MELTF-AS1 | 1.696996 | 1.04E-54 | 2.24E-52 | 18.56314 | Up | MELTF antisense RNA 1 |
| C1QTNF2 | 1.695983 | 1.18E-66 | 5.71E-64 | 21.4328 | Up | C1q and TNF related 2 |
| CYS1 | 1.688006 | 6.01E-69 | 3.57E-66 | 21.9875 | Up | cystin 1 |
| SLC6A4 | 1.687739 | 1.34E-23 | 2.31E-22 | 10.74587 | Up | solute carrier family 6 member 4 |
| LINC00670 | 1.682454 | 2.21E-52 | 3.9E-50 | 18.00406 | Up | long intergenic non-protein coding RNA 670 |
| COL14A1 | 1.681527 | 8.37E-50 | 1.15E-47 | 17.38423 | Up | collagen type XIV alpha 1 chain |
| AEBP1 | 1.678926 | 2.77E-45 | 2.51E-43 | 16.29209 | Up | AE binding protein 1 |
| TLL2 | 1.670045 | 4.29E-69 | 2.7E-66 | 22.023 | Up | tolloid like 2 |
| LOC107986163 | 1.669682 | 3.03E-33 | 1.1E-31 | 13.30833 | Up | uncharacterized LOC107986163 |
| NRK | 1.665994 | 9.57E-60 | 2.88E-57 | 19.7707 | Up | Nik related kinase |
| EXOC3L4 | 1.657167 | 1.47E-23 | 2.52E-22 | 10.73476 | Up | exocyst complex component 3 like 4 |
| HTR2A | 1.656339 | 9.15E-27 | 2.03E-25 | 11.61333 | Up | 5-hydroxytryptamine receptor 2A |
| HDC | 1.653783 | 5.15E-36 | 2.25E-34 | 14.00907 | Up | histidine decarboxylase |
| GSTM5 | 1.650191 | 4.21E-45 | 3.77E-43 | 16.24794 | Up | glutathione S-transferase mu 5 |
| CD3D | 1.641321 | 1.18E-31 | 3.86E-30 | 12.90043 | Up | CD3d molecule |
| ECRG4 | 1.632088 | 1.01E-46 | 1.06E-44 | 16.64023 | Up | ECRG4 augurin precursor |
| FAP | 1.627064 | 6.1E-37 | 2.9E-35 | 14.24099 | Up | fibroblast activation protein alpha |
| BPI | 1.624603 | 2.16E-31 | 6.91E-30 | 12.83272 | Up | bactericidal permeability increasing protein |
| CYP11A1 | 1.621266 | 4.82E-52 | 8.15E-50 | 17.92274 | Up | cytochrome P450 family 11 subfamily A member 1 |
| LCN12 | 1.619771 | 4.59E-32 | 1.55E-30 | 13.00586 | Up | lipocalin 12 |
| MFAP4 | 1.616857 | 5.78E-73 | 5.46E-70 | 22.96383 | Up | microfibril associated protein 4 |
| MYOC | 1.605119 | 1.96E-31 | 6.3E-30 | 12.84334 | Up | myocilin |
| THY1 | 1.604584 | 1.17E-22 | 1.87E-21 | 10.48087 | Up | Thy-1 cell surface antigen |
| ITGAL | 1.604327 | 4.27E-46 | 4.14E-44 | 16.48893 | Up | integrin subunit alpha L |
| KIAA1755 | 1.603838 | 2.97E-55 | 6.63E-53 | 18.69335 | Up | KIAA1755 |
| FMOD | 1.601342 | 5.22E-42 | 3.61E-40 | 15.49365 | Up | fibromodulin |
| GFRA3 | 1.59901 | 1.15E-35 | 4.93E-34 | 13.9215 | Up | GDNF family receptor alpha 3 |
| PHLDA1 | 1.593007 | 1.25E-63 | 4.89E-61 | 20.70408 | Up | pleckstrin homology like domain family A member 1 |
| SH2D1B | 1.592988 | 1.23E-30 | 3.7E-29 | 12.63695 | Up | SH2 domain containing 1B |
| IL34 | 1.590541 | 4.65E-46 | 4.48E-44 | 16.4798 | Up | interleukin 34 |
| ADAMTS14 | 1.590348 | 1.8E-36 | 8.26E-35 | 14.12324 | Up | ADAM metallopeptidase with thrombospondin type 1 motif 14 |
| APCDD1L-DT | 1.585375 | 8.02E-32 | 2.64E-30 | 12.94358 | Up | APCDD1L divergent transcript |
| CMTM2 | 1.584966 | 3.97E-22 | 5.98E-21 | 10.33056 | Up | CKLF like MARVEL transmembrane domain containing 2 |
| PROM1 | 1.584399 | 1.37E-53 | 2.85E-51 | 18.29382 | Up | prominin 1 |
| TENM4 | 1.57826 | 2.99E-46 | 2.97E-44 | 16.52634 | Up | teneurintransmembrane protein 4 |
| CD3E | 1.577059 | 1.44E-34 | 5.75E-33 | 13.64473 | Up | CD3e molecule |
| TMEM30B | 1.573717 | 8.62E-43 | 6.39E-41 | 15.68505 | Up | transmembrane protein 30B |
| SARDH | 1.564223 | 1.52E-43 | 1.2E-41 | 15.86877 | Up | sarcosine dehydrogenase |
| KLRB1 | 1.5636 | 2.78E-31 | 8.82E-30 | 12.80414 | Up | killer cell lectin like receptor B1 |
| NAPSB | 1.559889 | 6.93E-30 | 1.98E-28 | 12.44091 | Up | napsin B aspartic peptidase, pseudogene |
| SLCO4C1 | 1.559829 | 6.69E-31 | 2.06E-29 | 12.70541 | Up | solute carrier organic anion transporter family member 4C1 |
| GABRD | 1.559743 | 8.36E-25 | 1.58E-23 | 11.07971 | Up | gamma-aminobutyric acid type A receptor delta subunit |
| CD6 | 1.558091 | 6.44E-31 | 1.99E-29 | 12.70964 | Up | CD6 molecule |
| LINC02028 | 1.556023 | 2.42E-42 | 1.71E-40 | 15.57541 | Up | long intergenic non-protein coding RNA 2028 |
| RASL11B | 1.554174 | 6E-53 | 1.14E-50 | 18.14009 | Up | RAS like family 11 member B |
| TBX21 | 1.552213 | 4.5E-31 | 1.4E-29 | 12.74998 | Up | T-box transcription factor 21 |
| IL2RB | 1.546229 | 1.51E-39 | 8.57E-38 | 14.88881 | Up | interleukin 2 receptor subunit beta |
| HAAO | 1.54259 | 2.21E-46 | 2.23E-44 | 16.55819 | Up | 3-hydroxyanthranilate 3,4-dioxygenase |
| MS4A1 | 1.541164 | 1.65E-21 | 2.37E-20 | 10.15294 | Up | membrane spanning 4-domains A1 |
| LRRC17 | 1.539065 | 4.14E-53 | 8.04E-51 | 18.17887 | Up | leucine rich repeat containing 17 |
| CCL24 | 1.538643 | 5.69E-20 | 7.15E-19 | 9.70512 | Up | C-C motif chemokine ligand 24 |
| LINC00906 | 1.535912 | 2.36E-23 | 4E-22 | 10.67709 | Up | long intergenic non-protein coding RNA 906 |
| LMF1 | 1.535428 | 1.04E-41 | 6.99E-40 | 15.42078 | Up | lipase maturation factor 1 |
| TNFRSF4 | 1.535 | 8.46E-14 | 6.01E-13 | 7.761017 | Up | TNF receptor superfamily member 4 |
| P3H2 | 1.532878 | 2.18E-36 | 9.95E-35 | 14.10244 | Up | prolyl 3-hydroxylase 2 |
| LOC101926964 | 1.529325 | 1.15E-28 | 2.98E-27 | 12.12071 | Up | uncharacterized LOC101926964 |
| PTN | 1.528239 | 1.59E-62 | 5.8E-60 | 20.43819 | Up | pleiotrophin |
| EIF1AY | 1.527175 | 0.003828 | 0.007083 | 2.910526 | Up | eukaryotic translation initiation factor 1A Y-linked |
| DNAH8 | 1.526078 | 7.16E-28 | 1.73E-26 | 11.90949 | Up | dynein axonemal heavy chain 8 |
| GREM1 | 1.524172 | 2E-13 | 1.37E-12 | 7.633388 | Up | gremlin 1, DAN family BMP antagonist |
| CEACAM3 | 1.522791 | 2.87E-23 | 4.85E-22 | 10.65294 | Up | CEA cell adhesion molecule 3 |
| ATRNL1 | 1.52238 | 1.39E-22 | 2.19E-21 | 10.45995 | Up | attractin like 1 |
| FCN1 | 1.522239 | 4.41E-25 | 8.52E-24 | 11.15616 | Up | ficolin 1 |
| AMHR2 | 1.519307 | 1.07E-26 | 2.35E-25 | 11.59475 | Up | anti-Mullerian hormone receptor type 2 |
| CRHBP | 1.517324 | 7.94E-50 | 1.1E-47 | 17.38973 | Up | corticotropin releasing hormone binding protein |
| NME8 | 1.515954 | 5.23E-28 | 1.29E-26 | 11.94592 | Up | NME/NM23 family member 8 |
| ZAP70 | 1.514658 | 7.62E-33 | 2.68E-31 | 13.2059 | Up | zeta chain of T cell receptor associated protein kinase 70 |
| RPS4Y1 | 1.513872 | 0.003664 | 0.006805 | 2.924581 | Up | ribosomal protein S4 Y-linked 1 |
| LIPH | 1.512512 | 7.39E-38 | 3.72E-36 | 14.46955 | Up | lipase H |
| FAM181B | 1.509658 | 1.41E-29 | 3.94E-28 | 12.36002 | Up | family with sequence similarity 181 member B |
| LY9 | 1.506257 | 1.04E-25 | 2.1E-24 | 11.32764 | Up | lymphocyte antigen 9 |
| CCR7 | 1.505912 | 4.46E-24 | 7.96E-23 | 10.87865 | Up | C-C motif chemokine receptor 7 |
| FBXL16 | 1.500323 | 7.88E-30 | 2.24E-28 | 12.42638 | Up | F-box and leucine rich repeat protein 16 |
| IL17D | 1.492141 | 1.85E-48 | 2.26E-46 | 17.0602 | Up | interleukin 17D |
| FBLN7 | 1.490721 | 2.97E-38 | 1.54E-36 | 14.56812 | Up | fibulin 7 |
| CTD-3080P12.3 | 1.489908 | 3.27E-15 | 2.65E-14 | 8.231737 | Up | uncharacterized LOC101928857 |
| DIO2 | 1.489605 | 1.7E-50 | 2.45E-48 | 17.55098 | Up | iodothyroninedeiodinase 2 |
| ZBP1 | 1.487429 | 3.84E-33 | 1.37E-31 | 13.28194 | Up | Z-DNA binding protein 1 |
| SFTA1P | 1.486617 | 1.26E-25 | 2.52E-24 | 11.30493 | Up | surfactant associated 1, pseudogene |
| CD2 | 1.485796 | 9.06E-31 | 2.76E-29 | 12.6712 | Up | CD2 molecule |
| FCRL6 | 1.48399 | 2.5E-24 | 4.54E-23 | 10.94834 | Up | Fc receptor like 6 |
| FZD10-AS1 | 1.483735 | 3.65E-33 | 1.31E-31 | 13.28767 | Up | FZD10 antisense divergent transcript |
| CD1E | 1.483648 | 4.01E-21 | 5.57E-20 | 10.04196 | Up | CD1e molecule |
| COL16A1 | 1.481494 | 1.07E-46 | 1.11E-44 | 16.63474 | Up | collagen type XVI alpha 1 chain |
| EFCC1 | 1.476925 | 1.03E-45 | 9.65E-44 | 16.3961 | Up | EF-hand and coiled-coil domain containing 1 |
| ETV4 | 1.475328 | 1.73E-37 | 8.55E-36 | 14.37775 | Up | ETS variant transcription factor 4 |
| DLGAP1 | 1.473619 | 2.54E-25 | 4.99E-24 | 11.22147 | Up | DLG associated protein 1 |
| HYAL4 | 1.472457 | 1.68E-28 | 4.29E-27 | 12.07691 | Up | hyaluronidase 4 |
| MCHR1 | 1.471883 | 1.03E-24 | 1.94E-23 | 11.05502 | Up | melanin concentrating hormone receptor 1 |
| LOC101928731 | 1.467565 | 8.57E-36 | 3.7E-34 | 13.95348 | Up | uncharacterized LOC101928731 |
| TIGIT | 1.467506 | 9.66E-26 | 1.95E-24 | 11.33618 | Up | T cell immunoreceptor with Ig and ITIM domains |
| PRRT2 | 1.467191 | 4.38E-51 | 6.84E-49 | 17.69245 | Up | proline rich transmembrane protein 2 |
| CA3 | 1.462163 | 2.97E-20 | 3.81E-19 | 9.788456 | Up | carbonic anhydrase 3 |
| GADL1 | 1.461411 | 5.68E-20 | 7.14E-19 | 9.705276 | Up | glutamate decarboxylase like 1 |
| CD3G | 1.458347 | 4.25E-26 | 8.92E-25 | 11.4331 | Up | CD3g molecule |
| SPNS3 | 1.457263 | 4.56E-22 | 6.83E-21 | 10.31312 | Up | sphingolipid transporter 3 (putative) |
| FERMT1 | 1.456951 | 1.48E-33 | 5.45E-32 | 13.38789 | Up | fermitin family member 1 |
| GRIK4 | 1.454408 | 2.49E-31 | 7.95E-30 | 12.81649 | Up | glutamate ionotropic receptor kainate type subunit 4 |
| SDSL | 1.453371 | 8.03E-31 | 2.46E-29 | 12.68483 | Up | serine dehydratase like |
| NRG1 | 1.452944 | 9.93E-21 | 1.33E-19 | 9.927464 | Up | neuregulin 1 |
| TARP | 1.452369 | 1.3E-25 | 2.6E-24 | 11.30092 | Up | TCR gamma alternate reading frame protein |
| SCG2 | 1.450549 | 1.85E-16 | 1.69E-15 | 8.633202 | Up | secretogranin II |
| EGR2 | 1.448114 | 5.93E-26 | 1.22E-24 | 11.39379 | Up | early growth response 2 |
| GSG1L | 1.445137 | 1.2E-11 | 6.83E-11 | 7.00315 | Up | GSG1 like |
| SCARA3 | 1.444259 | 5.52E-63 | 2.11E-60 | 20.54857 | Up | scavenger receptor class A member 3 |
| UBASH3A | 1.439792 | 5.08E-25 | 9.75E-24 | 11.1393 | Up | ubiquitin associated and SH3 domain containing A |
| DMC1 | 1.439293 | 5.98E-61 | 2.04E-58 | 20.05974 | Up | DNA meiotic recombinase 1 |
| NGEF | 1.439001 | 1.44E-19 | 1.75E-18 | 9.585604 | Up | neuronal guanine nucleotide exchange factor |
| DNAJC22 | 1.437657 | 3.51E-29 | 9.52E-28 | 12.25626 | Up | DnaJ heat shock protein family (Hsp40) member C22 |
| SMPD3 | 1.436276 | 7.03E-22 | 1.04E-20 | 10.25951 | Up | sphingomyelinphosphodiesterase 3 |
| CCDC168 | 1.436207 | 4.17E-29 | 1.12E-27 | 12.23649 | Up | coiled-coil domain containing 168 |
| CRABP2 | 1.432348 | 7.38E-30 | 2.1E-28 | 12.43378 | Up | cellular retinoic acid binding protein 2 |
| PRPH | 1.430396 | 4.69E-28 | 1.16E-26 | 11.95832 | Up | peripherin |
| PALM3 | 1.430275 | 4.36E-28 | 1.08E-26 | 11.9668 | Up | paralemmin 3 |
| FOXS1 | 1.429428 | 2.74E-19 | 3.24E-18 | 9.502675 | Up | forkhead box S1 |
| ITIH5 | 1.429207 | 3.76E-74 | 4.34E-71 | 23.25374 | Up | inter-alpha-trypsin inhibitor heavy chain 5 |
| SKAP1 | 1.428481 | 2.6E-28 | 6.61E-27 | 12.02632 | Up | src kinase associated phosphoprotein 1 |
| ITGBL1 | 1.42419 | 1.72E-33 | 6.3E-32 | 13.37084 | Up | integrin subunit beta like 1 |
| COL8A2 | 1.424064 | 2.12E-45 | 1.95E-43 | 16.32026 | Up | collagen type VIII alpha 2 chain |
| KIRREL3 | 1.420945 | 8.98E-32 | 2.95E-30 | 12.93087 | Up | kirre like nephrin family adhesion molecule 3 |
| BEX1 | 1.420178 | 1.54E-22 | 2.41E-21 | 10.44694 | Up | brain expressed X-linked 1 |
| FCER2 | 1.416596 | 3.81E-22 | 5.76E-21 | 10.33555 | Up | Fc fragment of IgE receptor II |
| SLC24A2 | 1.415998 | 6.97E-21 | 9.53E-20 | 9.972135 | Up | solute carrier family 24 member 2 |
| TBC1D10C | 1.415564 | 3.36E-24 | 6.04E-23 | 10.91276 | Up | TBC1 domain family member 10C |
| ESR1 | 1.415141 | 6.38E-59 | 1.77E-56 | 19.57293 | Up | estrogen receptor 1 |
| SCN2B | 1.414446 | 3.55E-55 | 7.84E-53 | 18.67482 | Up | sodium voltage-gated channel beta subunit 2 |
| PADI4 | 1.413441 | 5.7E-17 | 5.47E-16 | 8.793997 | Up | peptidyl arginine deiminase 4 |
| OMG | 1.411113 | 2.11E-29 | 5.84E-28 | 12.31411 | Up | oligodendrocyte myelin glycoprotein |
| CTHRC1 | 1.410717 | 9.47E-28 | 2.26E-26 | 11.87721 | Up | collagen triple helix repeat containing 1 |
| LEF1 | 1.409598 | 1.53E-33 | 5.64E-32 | 13.38369 | Up | lymphoid enhancer binding factor 1 |
| SYTL1 | 1.405901 | 3.05E-23 | 5.12E-22 | 10.64567 | Up | synaptotagmin like 1 |
| CCL22 | 1.404475 | 2.6E-17 | 2.58E-16 | 8.900267 | Up | C-C motif chemokine ligand 22 |
| CLEC4F | 1.403759 | 3.69E-20 | 4.71E-19 | 9.760544 | Up | C-type lectin domain family 4 member F |
| P2RX6 | 1.403444 | 3.73E-27 | 8.5E-26 | 11.71788 | Up | purinergic receptor P2X 6 |
| TGFB2 | 1.399181 | 2.8E-29 | 7.65E-28 | 12.2821 | Up | transforming growth factor beta 2 |
| LINC00484 | 1.397493 | 1.11E-28 | 2.89E-27 | 12.12447 | Up | long intergenic non-protein coding RNA 484 |
| GALNT5 | 1.396805 | 7.38E-23 | 1.2E-21 | 10.53766 | Up | polypeptide N-acetylgalactosaminyltransferase 5 |
| LOC101929705 | 1.396659 | 2.98E-48 | 3.62E-46 | 17.01031 | Up | uncharacterized LOC101929705 |
| DACT2 | 1.393311 | 2.7E-16 | 2.43E-15 | 8.580885 | Up | dishevelled binding antagonist of beta catenin 2 |
| WNT10B | 1.388628 | 1.31E-24 | 2.44E-23 | 11.02636 | Up | Wnt family member 10B |
| MATN2 | 1.387237 | 1.33E-71 | 1.06E-68 | 22.63224 | Up | matrilin 2 |
| AZIN2 | 1.38529 | 1.78E-64 | 7.56E-62 | 20.90748 | Up | antizyme inhibitor 2 |
| B3GALT5 | 1.381072 | 1.64E-19 | 1.98E-18 | 9.568734 | Up | beta-1,3-galactosyltransferase 5 |
| LINC00968 | 1.380375 | 2.74E-31 | 8.72E-30 | 12.80576 | Up | long intergenic non-protein coding RNA 968 |
| RIMS4 | 1.378732 | 2.96E-24 | 5.34E-23 | 10.92819 | Up | regulating synaptic membrane exocytosis 4 |
| RASGRP1 | 1.37836 | 2.81E-28 | 7.1E-27 | 12.01751 | Up | RAS guanyl releasing protein 1 |
| CPXM2 | 1.376429 | 6.43E-39 | 3.5E-37 | 14.73275 | Up | carboxypeptidase X, M14 family member 2 |
| FMO1 | 1.375361 | 2.26E-21 | 3.2E-20 | 10.11404 | Up | flavin containing dimethylanilinemonoxygenase 1 |
| CD83 | 1.374802 | 5.06E-51 | 7.79E-49 | 17.67736 | Up | CD83 molecule |
| SYT17 | 1.374273 | 3.68E-62 | 1.32E-59 | 20.3506 | Up | synaptotagmin 17 |
| ABCG2 | 1.373113 | 9.52E-47 | 1E-44 | 16.64667 | Up | ATP binding cassette subfamily G member 2 (Junior blood group) |
| FAM133A | 1.369938 | 1.35E-18 | 1.5E-17 | 9.294288 | Up | family with sequence similarity 133 member A |
| FEZF1-AS1 | 1.36779 | 8.24E-23 | 1.34E-21 | 10.52416 | Up | FEZF1 antisense RNA 1 |
| DNAAF3 | 1.367564 | 6.54E-30 | 1.87E-28 | 12.44747 | Up | dynein axonemal assembly factor 3 |
| IGFN1 | 1.364677 | 5.51E-21 | 7.61E-20 | 10.00175 | Up | immunoglobulin like and fibronectin type III domain containing 1 |
| SH2D1A | 1.363761 | 7.86E-21 | 1.07E-19 | 9.957054 | Up | SH2 domain containing 1A |
| SRGAP3-AS4 | 1.363047 | 1.3E-18 | 1.45E-17 | 9.298963 | Up | SRGAP3 antisense RNA 4 |
| COL9A2 | 1.362168 | 4.17E-34 | 1.61E-32 | 13.52748 | Up | collagen type IX alpha 2 chain |
| SIT1 | 1.359564 | 7.31E-23 | 1.19E-21 | 10.5389 | Up | signaling threshold regulating transmembrane adaptor 1 |
| CHRDL1 | 1.359548 | 1.08E-18 | 1.21E-17 | 9.323916 | Up | chordin like 1 |
| RGS17 | 1.359486 | 6.69E-25 | 1.28E-23 | 11.10644 | Up | regulator of G protein signaling 17 |
| ACKR4 | 1.358827 | 1.17E-40 | 7.32E-39 | 15.16203 | Up | atypical chemokine receptor 4 |
| COL1A1 | 1.358302 | 1.03E-23 | 1.79E-22 | 10.77798 | Up | collagen type I alpha 1 chain |
| CD5 | 1.356328 | 1.97E-22 | 3.06E-21 | 10.41671 | Up | CD5 molecule |
| SOX8 | 1.353422 | 1.7E-41 | 1.13E-39 | 15.36824 | Up | SRY-box transcription factor 8 |
| LRRC77P | 1.351962 | 3.85E-39 | 2.11E-37 | 14.78807 | Up | leucine rich repeat containing 77, pseudogene |
| UNC5B-AS1 | 1.351117 | 1.06E-12 | 6.71E-12 | 7.381669 | Up | UNC5B antisense RNA 1 |
| SCG5 | 1.350517 | 2.08E-24 | 3.81E-23 | 10.97082 | Up | secretogranin V |
| LINC01267 | 1.348563 | 1.11E-18 | 1.25E-17 | 9.319702 | Up | long intergenic non-protein coding RNA 1267 |
| RXRG | 1.346393 | 4.44E-37 | 2.14E-35 | 14.2756 | Up | retinoid X receptor gamma |
| CD27 | 1.345829 | 1.13E-16 | 1.06E-15 | 8.700302 | Up | CD27 molecule |
| GLYATL2 | 1.342594 | 9.82E-35 | 3.98E-33 | 13.68647 | Up | glycine-N-acyltransferase like 2 |
| ANKRD24 | 1.342518 | 1.54E-25 | 3.07E-24 | 11.28066 | Up | ankyrin repeat domain 24 |
| INKA1 | 1.340521 | 8.59E-22 | 1.26E-20 | 10.23452 | Up | inka box actin regulator 1 |
| IFI44L | 1.338481 | 1E-44 | 8.57E-43 | 16.15631 | Up | interferon induced protein 44 like |
| MS4A2 | 1.33714 | 2.29E-16 | 2.08E-15 | 8.603622 | Up | membrane spanning 4-domains A2 |
| RHCG | 1.336804 | 4.25E-12 | 2.55E-11 | 7.166944 | Up | Rh family C glycoprotein |
| METTL21EP | 1.332538 | 1.34E-27 | 3.17E-26 | 11.83678 | Up | methyltransferase like 21E, pseudogene |
| CEMIP | 1.332429 | 4.08E-15 | 3.29E-14 | 8.200242 | Up | cell migration inducing hyaluronidase 1 |
| CD247 | 1.330672 | 4.07E-38 | 2.09E-36 | 14.53411 | Up | CD247 molecule |
| F2RL2 | 1.33014 | 1.01E-24 | 1.91E-23 | 11.05693 | Up | coagulation factor II thrombin receptor like 2 |
| IL31RA | 1.327493 | 7.49E-14 | 5.34E-13 | 7.779083 | Up | interleukin 31 receptor A |
| USP9Y | 1.322424 | 0.009551 | 0.016327 | 2.605368 | Up | ubiquitin specific peptidase 9 Y-linked |
| BRINP1 | 1.317235 | 1.88E-21 | 2.68E-20 | 10.13695 | Up | BMP/retinoic acid inducible neural specific 1 |
| MOXD1 | 1.313654 | 3.61E-40 | 2.16E-38 | 15.04169 | Up | monooxygenase DBH like 1 |
| MED12L | 1.312022 | 6.18E-28 | 1.5E-26 | 11.92652 | Up | mediator complex subunit 12L |
| LOC101927811 | 1.310553 | 1.22E-27 | 2.89E-26 | 11.84779 | Up | uncharacterized LOC101927811 |
| DNAJC27-AS1 | 1.309638 | 3.13E-32 | 1.06E-30 | 13.04877 | Up | DNAJC27 antisense RNA 1 |
| FAM225B | 1.309169 | 1.21E-25 | 2.43E-24 | 11.30969 | Up | family with sequence similarity 225 member B |
| SERPINE2 | 1.30842 | 1.21E-34 | 4.89E-33 | 13.66314 | Up | serpin family E member 2 |
| C1QTNF9 | 1.308078 | 6.34E-32 | 2.1E-30 | 12.96982 | Up | C1q and TNF related 9 |
| LMX1A | 1.305166 | 2.75E-22 | 4.22E-21 | 10.37562 | Up | LIM homeobox transcription factor 1 alpha |
| MIR181A2HG | 1.303865 | 6.57E-32 | 2.17E-30 | 12.96582 | Up | MIR181A2 host gene |
| THBS4 | 1.302766 | 3.46E-33 | 1.24E-31 | 13.2937 | Up | thrombospondin 4 |
| ROBO2 | 1.302226 | 4.74E-19 | 5.49E-18 | 9.431255 | Up | roundabout guidance receptor 2 |
| LOC105373878 | 1.300436 | 9.52E-38 | 4.77E-36 | 14.4422 | Up | uncharacterized LOC105373878 |
| ATP1B2 | 1.30027 | 1.37E-57 | 3.57E-55 | 19.25327 | Up | ATPase Na+/K+ transporting subunit beta 2 |
| FHAD1 | 1.29999 | 3.37E-26 | 7.14E-25 | 11.46057 | Up | forkhead associated phosphopeptide binding domain 1 |
| LCK | 1.297893 | 2.03E-30 | 5.98E-29 | 12.57993 | Up | LCK proto-oncogene, Src family tyrosine kinase |
| TMEM156 | 1.294685 | 1.97E-22 | 3.06E-21 | 10.41685 | Up | transmembrane protein 156 |
| LINC00211 | 1.293649 | 1.35E-30 | 4.05E-29 | 12.62631 | Up | long intergenic non-protein coding RNA 211 |
| PLEKHH2 | 1.29203 | 2.39E-30 | 7E-29 | 12.56159 | Up | pleckstrin homology, MyTH4 and FERM domain containing H2 |
| LINC01426 | 1.289853 | 9.64E-23 | 1.55E-21 | 10.50485 | Up | long intergenic non-protein coding RNA 1426 |
| ZMYND15 | 1.289009 | 7.84E-39 | 4.22E-37 | 14.71153 | Up | zinc finger MYND-type containing 15 |
| UCHL1 | 1.288567 | 6E-25 | 1.15E-23 | 11.11939 | Up | ubiquitin C-terminal hydrolase L1 |
| MYOZ1 | 1.288488 | 2.05E-25 | 4.04E-24 | 11.24716 | Up | myozenin 1 |
| MAP3K7CL | 1.288168 | 8.27E-21 | 1.12E-19 | 9.950544 | Up | MAP3K7 C-terminal like |
| PLCH2 | 1.287447 | 1.12E-18 | 1.26E-17 | 9.318392 | Up | phospholipase C eta 2 |
| MRC2 | 1.286807 | 6.94E-34 | 2.63E-32 | 13.47131 | Up | mannose receptor C type 2 |
| ADAMTSL1 | 1.286675 | 1.51E-31 | 4.89E-30 | 12.87241 | Up | ADAMTS like 1 |
| PDE4C | 1.285231 | 2.27E-38 | 1.19E-36 | 14.59704 | Up | phosphodiesterase 4C |
| CXCL14 | 1.281807 | 6.76E-16 | 5.83E-15 | 8.453562 | Up | C-X-C motif chemokine ligand 14 |
| GDNF | 1.280842 | 2.33E-24 | 4.25E-23 | 10.95683 | Up | glial cell derived neurotrophic factor |
| ASB18 | 1.280174 | 5.71E-37 | 2.73E-35 | 14.24819 | Up | ankyrin repeat and SOCS box containing 18 |
| HHIP | 1.278593 | 7.98E-19 | 9.06E-18 | 9.363129 | Up | hedgehog interacting protein |
| KDM5D | 1.277307 | 0.011648 | 0.019535 | 2.535361 | Up | lysine demethylase 5D |
| MIR3142HG | 1.277276 | 1.35E-26 | 2.93E-25 | 11.5679 | Up | MIR3142 host gene |
| LTBP2 | 1.274413 | 1.1E-34 | 4.45E-33 | 13.67374 | Up | latent transforming growth factor beta binding protein 2 |
| IRX6 | 1.271621 | 1.65E-28 | 4.22E-27 | 12.079 | Up | iroquoishomeobox 6 |
| CLEC11A | 1.271266 | 1.43E-22 | 2.25E-21 | 10.45611 | Up | C-type lectin domain containing 11A |
| IDO1 | 1.270315 | 2.09E-16 | 1.91E-15 | 8.615828 | Up | indoleamine 2,3-dioxygenase 1 |
| C1QTNF9B | 1.268225 | 7.19E-17 | 6.84E-16 | 8.76235 | Up | C1q and TNF related 9B |
| CRYM | 1.266414 | 1.15E-47 | 1.3E-45 | 16.86886 | Up | crystallin mu |
| EFCAB1 | 1.265871 | 4.84E-24 | 8.61E-23 | 10.86895 | Up | EF-hand calcium binding domain 1 |
| DDX3Y | 1.264424 | 0.013065 | 0.021716 | 2.494173 | Up | DEAD-box helicase 3 Y-linked |
| CHIT1 | 1.264407 | 4.27E-10 | 2.05E-09 | 6.417928 | Up | chitinase 1 |
| GNG8 | 1.2627 | 8.4E-11 | 4.37E-10 | 6.689199 | Up | G protein subunit gamma 8 |
| NT5E | 1.261748 | 1.1E-52 | 2.02E-50 | 18.07717 | Up | 5'-nucleotidase ecto |
| HCG20 | 1.261078 | 2.49E-24 | 4.52E-23 | 10.94906 | Up | HLA complex group 20 |
| NEURL1 | 1.260771 | 1.31E-24 | 2.45E-23 | 11.0258 | Up | neuralized E3 ubiquitin protein ligase 1 |
| EYS | 1.260064 | 7.24E-19 | 8.26E-18 | 9.375881 | Up | eyes shut homolog |
| GLI2 | 1.255414 | 6.95E-43 | 5.21E-41 | 15.70783 | Up | GLI family zinc finger 2 |
| LINC01133 | 1.254973 | 2.04E-14 | 1.54E-13 | 7.969277 | Up | long intergenic non-protein coding RNA 1133 |
| LOC105371795 | 1.253649 | 3.74E-17 | 3.66E-16 | 8.851004 | Up | uncharacterized LOC105371795 |
| DPT | 1.250345 | 5.78E-42 | 3.96E-40 | 15.48293 | Up | dermatopontin |
| PRR15 | 1.249204 | 2.22E-21 | 3.15E-20 | 10.11632 | Up | proline rich 15 |
| CTNNA2 | 1.248053 | 6.25E-22 | 9.25E-21 | 10.2742 | Up | catenin alpha 2 |
| HMCN2 | 1.24733 | 1.65E-24 | 3.05E-23 | 10.99834 | Up | hemicentin 2 |
| ITGB2-AS1 | 1.247241 | 6.45E-29 | 1.72E-27 | 12.18654 | Up | ITGB2 antisense RNA 1 |
| TLR7 | 1.246342 | 4.37E-26 | 9.15E-25 | 11.42984 | Up | toll like receptor 7 |
| HLA-DQA1 | 1.244186 | 3.69E-21 | 5.15E-20 | 10.05236 | Up | major histocompatibility complex, class II, DQ alpha 1 |
| TAC4 | 1.24286 | 1.46E-20 | 1.91E-19 | 9.878964 | Up | tachykinin precursor 4 |
| LRRC52-AS1 | 1.241509 | 7.78E-13 | 5.01E-12 | 7.428482 | Up | LRRC52 antisense RNA 1 |
| PPM1K-DT | 1.240771 | 1.97E-10 | 9.85E-10 | 6.547691 | Up | PPM1K divergent transcript |
| PIEZO2 | 1.240568 | 7.72E-16 | 6.63E-15 | 8.434952 | Up | piezo type mechanosensitive ion channel component 2 |
| CCDC80 | 1.239608 | 1.42E-30 | 4.24E-29 | 12.62065 | Up | coiled-coil domain containing 80 |
| DUSP15 | 1.239063 | 4.11E-35 | 1.7E-33 | 13.78207 | Up | dual specificity phosphatase 15 |
| GNLY | 1.238419 | 1.78E-27 | 4.16E-26 | 11.80405 | Up | granulysin |
| CNTNAP2 | 1.235959 | 5.04E-18 | 5.34E-17 | 9.11997 | Up | contactin associated protein like 2 |
| PRRX2 | 1.235523 | 3.93E-12 | 2.36E-11 | 7.178965 | Up | paired related homeobox 2 |
| DUOX2 | 1.23448 | 5.79E-28 | 1.41E-26 | 11.93408 | Up | dual oxidase 2 |
| GNA14 | 1.233753 | 1.61E-50 | 2.34E-48 | 17.55626 | Up | G protein subunit alpha 14 |
| PMP2 | 1.233661 | 5.07E-20 | 6.39E-19 | 9.719945 | Up | peripheral myelin protein 2 |
| FOXO6-AS1 | 1.231713 | 1.06E-20 | 1.42E-19 | 9.918623 | Up | FOXO6 antisense RNA 1 |
| MALRD1 | 1.231194 | 3.34E-18 | 3.59E-17 | 9.174723 | Up | MAM and LDL receptor class A domain containing 1 |
| PHF21B | 1.227657 | 1.22E-30 | 3.69E-29 | 12.63719 | Up | PHD finger protein 21B |
| OLFML1 | 1.224413 | 5.26E-65 | 2.38E-62 | 21.03513 | Up | olfactomedin like 1 |
| TRIM17 | 1.221292 | 5.11E-26 | 1.06E-24 | 11.41147 | Up | tripartite motif containing 17 |
| TSPAN10 | 1.220763 | 5.54E-31 | 1.72E-29 | 12.72652 | Up | tetraspanin 10 |
| IER3 | 1.220245 | 1.19E-22 | 1.9E-21 | 10.4788 | Up | immediate early response 3 |
| NUP62CL | 1.219897 | 2.88E-34 | 1.12E-32 | 13.56819 | Up | nucleoporin 62 C-terminal like |
| GPA33 | 1.219144 | 1.29E-23 | 2.22E-22 | 10.75057 | Up | glycoprotein A33 |
| CLEC9A | 1.21506 | 1.84E-16 | 1.69E-15 | 8.633518 | Up | C-type lectin domain containing 9A |
| HMGA2-AS1 | 1.214586 | 5.25E-21 | 7.26E-20 | 10.00787 | Up | HMGA2 antisense RNA 1 |
| LUARIS | 1.213922 | 1.79E-17 | 1.81E-16 | 8.950795 | Up | lncRNAupregulator of antiviral response interferon signaling |
| KCNN3 | 1.211097 | 5.05E-50 | 7.1E-48 | 17.43693 | Up | potassium calcium-activated channel subfamily N member 3 |
| MSS51 | 1.211068 | 4.05E-43 | 3.1E-41 | 15.76499 | Up | MSS51 mitochondrial translational activator |
| LOC286178 | 1.203156 | 3.35E-17 | 3.28E-16 | 8.866208 | Up | uncharacterized LOC286178 |
| BCL11B | 1.200808 | 5E-21 | 6.92E-20 | 10.01416 | Up | BAF chromatin remodeling complex subunit BCL11B |
| FSCN1 | 1.200742 | 5.5E-22 | 8.18E-21 | 10.29007 | Up | fascin actin-bundling protein 1 |
| ARSI | 1.199759 | 1.81E-14 | 1.37E-13 | 7.986748 | Up | arylsulfatase family member I |
| TP63 | 1.199611 | 1.25E-32 | 4.34E-31 | 13.15108 | Up | tumor protein p63 |
| APOA1-AS | 1.198437 | 1.23E-11 | 6.96E-11 | 6.999998 | Up | APOA1 antisense RNA |
| LINC01531 | 1.197352 | 5.61E-13 | 3.67E-12 | 7.478264 | Up | long intergenic non-protein coding RNA 1531 |
| CD40LG | 1.196777 | 8.21E-16 | 7.01E-15 | 8.42641 | Up | CD40 ligand |
| CAMK4 | 1.196139 | 1.86E-19 | 2.23E-18 | 9.552711 | Up | calcium/calmodulin dependent protein kinase IV |
| TM6SF2 | 1.194983 | 2.63E-30 | 7.66E-29 | 12.55083 | Up | transmembrane 6 superfamily member 2 |
| TRAF3IP3 | 1.193405 | 4.01E-36 | 1.79E-34 | 14.03636 | Up | TRAF3 interacting protein 3 |
| SIGLEC8 | 1.192 | 1.1E-18 | 1.23E-17 | 9.321523 | Up | sialic acid binding Ig like lectin 8 |
| WDR66 | 1.191782 | 5.11E-49 | 6.63E-47 | 17.19495 | Up | WD repeat domain 66 |
| ALOX15 | 1.189497 | 2.11E-14 | 1.59E-13 | 7.96447 | Up | arachidonate 15-lipoxygenase |
| KLHL35 | 1.186728 | 4.84E-19 | 5.59E-18 | 9.428585 | Up | kelch like family member 35 |
| ITGB7 | 1.184564 | 1.14E-39 | 6.55E-38 | 14.91886 | Up | integrin subunit beta 7 |
| SEC14L6 | 1.18353 | 2.24E-18 | 2.45E-17 | 9.227693 | Up | SEC14 like lipid binding 6 |
| PDIA2 | 1.181381 | 8.1E-21 | 1.1E-19 | 9.953216 | Up | protein disulfideisomerase family A member 2 |
| LGI1 | 1.180437 | 1.29E-19 | 1.57E-18 | 9.600238 | Up | leucine rich glioma inactivated 1 |
| PPDPF | 1.180084 | 2.23E-16 | 2.03E-15 | 8.60702 | Up | pancreatic progenitor cell differentiation and proliferation factor |
| SYTL2 | 1.179465 | 1.01E-34 | 4.07E-33 | 13.68374 | Up | synaptotagmin like 2 |
| ADAMTSL2 | 1.179365 | 2.6E-22 | 4E-21 | 10.38261 | Up | ADAMTS like 2 |
| FZD2 | 1.178996 | 1.82E-18 | 2E-17 | 9.255263 | Up | frizzled class receptor 2 |
| LAMB4 | 1.17768 | 7.69E-18 | 8.02E-17 | 9.063817 | Up | laminin subunit beta 4 |
| NRIP2 | 1.176935 | 9.58E-49 | 1.2E-46 | 17.12912 | Up | nuclear receptor interacting protein 2 |
| SULF1 | 1.175739 | 2.88E-38 | 1.49E-36 | 14.57145 | Up | sulfatase 1 |
| ETV7 | 1.175707 | 8.16E-17 | 7.74E-16 | 8.745045 | Up | ETS variant transcription factor 7 |
| ANKRD36BP2 | 1.171138 | 3.75E-18 | 4.02E-17 | 9.159248 | Up | ankyrin repeat domain 36B pseudogene 2 |
| COLQ | 1.170037 | 5.17E-28 | 1.27E-26 | 11.94726 | Up | collagen like tail subunit of asymmetric acetylcholinesterase |
| PODXL2 | 1.169919 | 8.66E-47 | 9.23E-45 | 16.65665 | Up | podocalyxin like 2 |
| LINC00501 | 1.169906 | 9.77E-31 | 2.97E-29 | 12.6627 | Up | long intergenic non-protein coding RNA 501 |
| CARD11 | 1.169111 | 1.27E-22 | 2.01E-21 | 10.47111 | Up | caspase recruitment domain family member 11 |
| ANO9 | 1.168436 | 6.51E-16 | 5.62E-15 | 8.458675 | Up | anoctamin 9 |
| SERTAD4 | 1.167618 | 5.18E-28 | 1.28E-26 | 11.94687 | Up | SERTA domain containing 4 |
| PYHIN1 | 1.16663 | 1.63E-21 | 2.34E-20 | 10.15473 | Up | pyrin and HIN domain family member 1 |
| CD48 | 1.165664 | 5.88E-26 | 1.21E-24 | 11.39481 | Up | CD48 molecule |
| C12orf75 | 1.164765 | 1.52E-28 | 3.92E-27 | 12.08817 | Up | chromosome 12 open reading frame 75 |
| HTR2B | 1.161887 | 2.28E-18 | 2.49E-17 | 9.225086 | Up | 5-hydroxytryptamine receptor 2B |
| ECM2 | 1.161863 | 9.81E-31 | 2.97E-29 | 12.66225 | Up | extracellular matrix protein 2 |
| PDE5A | 1.160025 | 1.39E-39 | 7.94E-38 | 14.89735 | Up | phosphodiesterase 5A |
| GZMK | 1.159035 | 4.82E-19 | 5.58E-18 | 9.429051 | Up | granzyme K |
| TEX41 | 1.15826 | 7.5E-23 | 1.22E-21 | 10.53572 | Up | testis expressed 41 |
| IL1RL1 | -4.16665 | 1.16E-92 | 8.03E-89 | -27.8886 | Down | interleukin 1 receptor like 1 |
| RNASE2 | -3.13727 | 1.02E-71 | 8.46E-69 | -22.6604 | Down | ribonuclease A family member 2 |
| ALOX15B | -3.04933 | 8.54E-42 | 5.82E-40 | -15.4414 | Down | arachidonate 15-lipoxygenase type B |
| LCN10 | -3.01762 | 7.59E-61 | 2.5E-58 | -20.0349 | Down | lipocalin 10 |
| TUBA3E | -2.99401 | 5.3E-73 | 5.25E-70 | -22.973 | Down | tubulin alpha 3e |
| TUBA3D | -2.93212 | 1.14E-70 | 8.14E-68 | -22.4056 | Down | tubulin alpha 3d |
| MYH6 | -2.7545 | 1.4E-76 | 2.23E-73 | -23.8495 | Down | myosin heavy chain 6 |
| PI15 | -2.74552 | 3.93E-40 | 2.34E-38 | -15.0328 | Down | peptidase inhibitor 15 |
| TUBA3C | -2.74348 | 1.64E-56 | 4E-54 | -18.9952 | Down | tubulin alpha 3c |
| CYP4B1 | -2.71576 | 9.65E-56 | 2.23E-53 | -18.8104 | Down | cytochrome P450 family 4 subfamily B member 1 |
| NPTX2 | -2.59581 | 1.15E-68 | 6.46E-66 | -21.9193 | Down | neuronal pentraxin 2 |
| AOX1 | -2.57613 | 8.34E-69 | 4.81E-66 | -21.953 | Down | aldehyde oxidase 1 |
| PLA2G2A | -2.55621 | 2.06E-43 | 1.6E-41 | -15.8368 | Down | phospholipase A2 group IIA |
| LCN6 | -2.54437 | 7.91E-59 | 2.16E-56 | -19.5506 | Down | lipocalin 6 |
| CD163 | -2.50965 | 1.12E-85 | 2.6E-82 | -26.1108 | Down | CD163 molecule |
| GNMT | -2.50909 | 2.21E-57 | 5.61E-55 | -19.2036 | Down | glycine N-methyltransferase |
| AQP4 | -2.50556 | 1.63E-36 | 7.51E-35 | -14.1345 | Down | aquaporin 4 |
| SCGN | -2.48857 | 7.7E-44 | 6.13E-42 | -15.9409 | Down | secretagogin, EF-hand calcium binding protein |
| FCN3 | -2.48214 | 5.69E-69 | 3.48E-66 | -21.9932 | Down | ficolin 3 |
| SLCO4A1 | -2.41668 | 4.74E-68 | 2.46E-65 | -21.7703 | Down | solute carrier organic anion transporter family member 4A1 |
| PPEF1 | -2.40843 | 4.16E-51 | 6.54E-49 | -17.6979 | Down | protein phosphatase with EF-hand domain 1 |
| SGPP2 | -2.38709 | 1.41E-45 | 1.31E-43 | -16.3629 | Down | sphingosine-1-phosphate phosphatase 2 |
| SAA1 | -2.36387 | 6.15E-14 | 4.41E-13 | -7.80794 | Down | serum amyloid A1 |
| IL1R2 | -2.33348 | 8.84E-47 | 9.38E-45 | -16.6544 | Down | interleukin 1 receptor type 2 |
| OLAH | -2.32381 | 1.23E-49 | 1.65E-47 | -17.3439 | Down | oleoyl-ACP hydrolase |
| LCN15 | -2.30949 | 1.71E-34 | 6.83E-33 | -13.6252 | Down | lipocalin 15 |
| CYP4Z1 | -2.2978 | 4.89E-54 | 1.03E-51 | -18.4015 | Down | cytochrome P450 family 4 subfamily Z member 1 |
| OVOS2 | -2.29611 | 2.76E-28 | 6.99E-27 | -12.0196 | Down | alpha-2-macroglobulin like 1 pseudogene |
| IL10 | -2.26244 | 1.07E-46 | 1.11E-44 | -16.6348 | Down | interleukin 10 |
| CD177 | -2.24129 | 1.27E-25 | 2.55E-24 | -11.3037 | Down | CD177 molecule |
| GALNT15 | -2.23547 | 2.82E-69 | 1.83E-66 | -22.067 | Down | polypeptide N-acetylgalactosaminyltransferase 15 |
| FKBP5 | -2.20125 | 1.02E-64 | 4.52E-62 | -20.9657 | Down | FKBP prolylisomerase 5 |
| HOPX | -2.19678 | 2.33E-39 | 1.3E-37 | -14.842 | Down | HOP homeobox |
| MCEMP1 | -2.16877 | 7.4E-33 | 2.6E-31 | -13.2092 | Down | mast cell expressed membrane protein 1 |
| SYN2 | -2.13215 | 4.46E-44 | 3.61E-42 | -15.9986 | Down | synapsin II |
| SERPINA3 | -2.12094 | 2.93E-28 | 7.39E-27 | -12.0128 | Down | serpin family A member 3 |
| TCF24 | -2.09501 | 6.7E-26 | 1.37E-24 | -11.3795 | Down | transcription factor 24 |
| CFTR | -2.08432 | 2.72E-36 | 1.23E-34 | -14.0784 | Down | CF transmembrane conductance regulator |
| RARRES1 | -2.08128 | 1.7E-44 | 1.43E-42 | -16.1007 | Down | retinoic acid receptor responder 1 |
| EREG | -2.0761 | 5.3E-22 | 7.89E-21 | -10.2947 | Down | epiregulin |
| LBP | -2.0676 | 1.07E-20 | 1.43E-19 | -9.91761 | Down | lipopolysaccharide binding protein |
| C4orf54 | -2.05065 | 8.08E-20 | 9.99E-19 | -9.66017 | Down | chromosome 4 open reading frame 54 |
| LGI3 | -2.04234 | 5.28E-32 | 1.77E-30 | -12.9903 | Down | leucine rich repeat LGI family member 3 |
| BLM | -2.03762 | 5.04E-74 | 5.51E-71 | -23.2226 | Down | BLM RecQ like helicase |
| SULT1B1 | -2.03126 | 2.66E-49 | 3.5E-47 | -17.263 | Down | sulfotransferase family 1B member 1 |
| C1orf105 | -2.02075 | 2.12E-41 | 1.4E-39 | -15.3444 | Down | chromosome 1 open reading frame 105 |
| ADAMTS4 | -2.01588 | 5.48E-45 | 4.87E-43 | -16.2199 | Down | ADAM metallopeptidase with thrombospondin type 1 motif 4 |
| HMGCS2 | -2.00935 | 9.11E-18 | 9.44E-17 | -9.04123 | Down | 3-hydroxy-3-methylglutaryl-CoA synthase 2 |
| VSIG4 | -1.98626 | 3.18E-76 | 4.71E-73 | -23.7619 | Down | V-set and immunoglobulin domain containing 4 |
| STAC2 | -1.96935 | 2.46E-34 | 9.76E-33 | -13.5853 | Down | SH3 and cysteine rich domain 2 |
| FGF10 | -1.92961 | 4.3E-26 | 9.02E-25 | -11.4317 | Down | fibroblast growth factor 10 |
| LYVE1 | -1.92131 | 1.57E-62 | 5.8E-60 | -20.4397 | Down | lymphatic vessel endothelial hyaluronan receptor 1 |
| LAD1 | -1.91982 | 9.62E-41 | 6.02E-39 | -15.1832 | Down | ladinin 1 |
| WNK3 | -1.9035 | 3.09E-61 | 1.09E-58 | -20.1286 | Down | WNK lysine deficient protein kinase 3 |
| DHRS7C | -1.88942 | 1.04E-19 | 1.28E-18 | -9.62709 | Down | dehydrogenase/reductase 7C |
| NMRAL2P | -1.86141 | 1.01E-41 | 6.86E-40 | -15.4235 | Down | NmrA like redox sensor 2, pseudogene |
| GMNC | -1.83731 | 6.65E-29 | 1.76E-27 | -12.1831 | Down | geminin coiled-coil domain containing |
| METTL7B | -1.83099 | 2.07E-40 | 1.25E-38 | -15.1014 | Down | methyltransferase like 7B |
| PLA2G4F | -1.81396 | 4.24E-24 | 7.58E-23 | -10.8848 | Down | phospholipase A2 group IVF |
| SLC11A1 | -1.8014 | 5.26E-44 | 4.24E-42 | -15.9811 | Down | solute carrier family 11 member 1 |
| LINC00964 | -1.77887 | 1.45E-36 | 6.73E-35 | -14.1472 | Down | long intergenic non-protein coding RNA 964 |
| LINC02207 | -1.76058 | 3.51E-29 | 9.52E-28 | -12.2562 | Down | long intergenic non-protein coding RNA 2207 |
| OVCH1 | -1.75228 | 1.32E-37 | 6.59E-36 | -14.4065 | Down | ovochymase 1 |
| RHBDL3 | -1.74833 | 8.3E-31 | 2.54E-29 | -12.681 | Down | rhomboid like 3 |
| MGST1 | -1.73713 | 2.06E-39 | 1.16E-37 | -14.8552 | Down | microsomal glutathione S-transferase 1 |
| MARCO | -1.7321 | 5.53E-16 | 4.82E-15 | -8.48151 | Down | macrophage receptor with collagenous structure |
| SLC25A18 | -1.69891 | 5.06E-39 | 2.77E-37 | -14.7586 | Down | solute carrier family 25 member 18 |
| MANCR | -1.6978 | 1.89E-40 | 1.15E-38 | -15.1112 | Down | mitotically associated long non coding RNA |
| TBC1D29P | -1.6943 | 1.92E-27 | 4.46E-26 | -11.7954 | Down | TBC1 domain family member 29, pseudogene |
| IL17RB | -1.68748 | 5.8E-43 | 4.39E-41 | -15.727 | Down | interleukin 17 receptor B |
| C5orf64 | -1.66664 | 9.16E-29 | 2.4E-27 | -12.1463 | Down | chromosome 5 open reading frame 64 |
| TDRD9 | -1.65083 | 3.47E-52 | 5.96E-50 | -17.9571 | Down | tudor domain containing 9 |
| CNTN3 | -1.65075 | 1.17E-29 | 3.28E-28 | -12.3817 | Down | contactin 3 |
| SSTR5 | -1.64589 | 1.42E-10 | 7.2E-10 | -6.60229 | Down | somatostatin receptor 5 |
| FAM155B | -1.64483 | 3.91E-31 | 1.22E-29 | -12.7659 | Down | family with sequence similarity 155 member B |
| TMEFF2 | -1.63641 | 9.72E-21 | 1.3E-19 | -9.9302 | Down | transmembrane protein with EGF like and two follistatin like domains 2 |
| SIGLEC9 | -1.61765 | 9.97E-57 | 2.47E-54 | -19.0468 | Down | sialic acid binding Ig like lectin 9 |
| IL18R1 | -1.61068 | 2.84E-55 | 6.41E-53 | -18.698 | Down | interleukin 18 receptor 1 |
| AQP3 | -1.59794 | 1.16E-48 | 1.44E-46 | -17.1093 | Down | aquaporin 3 (Gill blood group) |
| SLC38A8 | -1.59297 | 8.76E-15 | 6.82E-14 | -8.09114 | Down | solute carrier family 38 member 8 |
| TMIGD3 | -1.59103 | 1.26E-42 | 9.18E-41 | -15.6445 | Down | transmembrane and immunoglobulin domain containing 3 |
| MT1A | -1.58793 | 1.47E-13 | 1.02E-12 | -7.67924 | Down | metallothionein 1A |
| CA14 | -1.58283 | 5.77E-28 | 1.41E-26 | -11.9346 | Down | carbonic anhydrase 14 |
| CPM | -1.5817 | 1.23E-50 | 1.8E-48 | -17.5848 | Down | carboxypeptidase M |
| ETNPPL | -1.55969 | 1.83E-29 | 5.09E-28 | -12.3302 | Down | ethanolamine-phosphate phospho-lyase |
| SLC66A1L | -1.54109 | 1.16E-43 | 9.14E-42 | -15.8978 | Down | solute carrier family 66 member 1 like |
| GPR84 | -1.5373 | 1.18E-21 | 1.71E-20 | -10.1951 | Down | G protein-coupled receptor 84 |
| HOOK1 | -1.53589 | 2.71E-43 | 2.09E-41 | -15.8079 | Down | hook microtubule tethering protein 1 |
| CHDH | -1.52384 | 2.82E-55 | 6.41E-53 | -18.6987 | Down | choline dehydrogenase |
| SERTM1 | -1.52131 | 7.14E-21 | 9.74E-20 | -9.96917 | Down | serine rich and transmembrane domain containing 1 |
| SHISA3 | -1.51927 | 2.09E-36 | 9.56E-35 | -14.107 | Down | shisa family member 3 |
| PCSK1 | -1.50962 | 2.47E-21 | 3.49E-20 | -10.1028 | Down | proproteinconvertasesubtilisin/kexin type 1 |
| ADH1B | -1.49531 | 3.76E-35 | 1.57E-33 | -13.7917 | Down | alcohol dehydrogenase 1B (class I), beta polypeptide |
| CATSPERB | -1.48402 | 3.22E-35 | 1.35E-33 | -13.8089 | Down | cation channel sperm associated auxiliary subunit beta |
| CD38 | -1.48372 | 1.86E-52 | 3.3E-50 | -18.0222 | Down | CD38 molecule |
| POM121L9P | -1.47176 | 1.12E-54 | 2.41E-52 | -18.5546 | Down | POM121 transmembranenucleoporin like 9, pseudogene |
| LINC01166 | -1.47139 | 3.94E-20 | 5E-19 | -9.75233 | Down | long intergenic non-protein coding RNA 1166 |
| RDH10-AS1 | -1.47137 | 1.52E-36 | 7.04E-35 | -14.142 | Down | RDH10 antisense RNA 1 |
| CACNA1E | -1.46899 | 4.75E-19 | 5.5E-18 | -9.43103 | Down | calcium voltage-gated channel subunit alpha1 E |
| PAQR5 | -1.45849 | 2.86E-37 | 1.4E-35 | -14.323 | Down | progestin and adipoQ receptor family member 5 |
| LINC02388 | -1.45595 | 2.84E-15 | 2.32E-14 | -8.25187 | Down | long intergenic non-protein coding RNA 2388 |
| LINC01134 | -1.44716 | 4.56E-31 | 1.42E-29 | -12.7486 | Down | long intergenic non-protein coding RNA 1134 |
| HPCAL4 | -1.44324 | 1.12E-20 | 1.49E-19 | -9.91235 | Down | hippocalcin like 4 |
| BMP7 | -1.43955 | 1.41E-20 | 1.85E-19 | -9.88326 | Down | bone morphogenetic protein 7 |
| SERPINE1 | -1.43704 | 5.19E-17 | 5.01E-16 | -8.80674 | Down | serpin family E member 1 |
| KNG1 | -1.43341 | 1.09E-20 | 1.45E-19 | -9.916 | Down | kininogen 1 |
| MCM10 | -1.42685 | 7.91E-27 | 1.76E-25 | -11.6304 | Down | minichromosome maintenance 10 replication initiation factor |
| SLC22A16 | -1.41592 | 1.05E-21 | 1.52E-20 | -10.2097 | Down | solute carrier family 22 member 16 |
| LOC105378909 | -1.41396 | 3.53E-27 | 8.05E-26 | -11.7245 | Down | uncharacterized LOC105378909 |
| TMEM151B | -1.41097 | 3.2E-11 | 1.74E-10 | -6.84623 | Down | transmembrane protein 151B |
| WNT9B | -1.40828 | 2.89E-27 | 6.65E-26 | -11.7476 | Down | Wnt family member 9B |
| ST6GALNAC3 | -1.4044 | 8.83E-77 | 1.53E-73 | -23.8986 | Down | ST6 N-acetylgalactosaminide alpha-2,6-sialyltransferase 3 |
| ZBTB16 | -1.40189 | 1.08E-26 | 2.37E-25 | -11.594 | Down | zinc finger and BTB domain containing 16 |
| LINC01088 | -1.39856 | 2.94E-29 | 8.01E-28 | -12.2765 | Down | long intergenic non-protein coding RNA 1088 |
| ANPEP | -1.39173 | 1.79E-35 | 7.6E-34 | -13.8727 | Down | alanylaminopeptidase, membrane |
| CHST9 | -1.39105 | 2.86E-17 | 2.83E-16 | -8.88726 | Down | carbohydrate sulfotransferase 9 |
| CHAC2 | -1.39049 | 4.46E-51 | 6.91E-49 | -17.6907 | Down | ChaCcation transport regulator homolog 2 |
| ANKRD2 | -1.38594 | 1.02E-21 | 1.49E-20 | -10.2128 | Down | ankyrin repeat domain 2 |
| EDNRB | -1.37805 | 1.43E-45 | 1.32E-43 | -16.3619 | Down | endothelin receptor type B |
| TMEM132B | -1.37526 | 5.39E-29 | 1.44E-27 | -12.207 | Down | transmembrane protein 132B |
| SSTR2 | -1.36986 | 6.6E-38 | 3.34E-36 | -14.4818 | Down | somatostatin receptor 2 |
| TFCP2L1 | -1.36492 | 1.52E-30 | 4.54E-29 | -12.6127 | Down | transcription factor CP2 like 1 |
| LMAN1L | -1.35734 | 9.77E-15 | 7.57E-14 | -8.07551 | Down | lectin, mannose binding 1 like |
| NSG1 | -1.35624 | 1.61E-34 | 6.43E-33 | -13.6323 | Down | neuronal vesicle trafficking associated 1 |
| SCGB1D2 | -1.34714 | 1.13E-14 | 8.67E-14 | -8.05497 | Down | secretoglobin family 1D member 2 |
| S1PR3 | -1.33974 | 6.86E-68 | 3.48E-65 | -21.7316 | Down | sphingosine-1-phosphate receptor 3 |
| HP | -1.33891 | 2.46E-11 | 1.36E-10 | -6.88868 | Down | haptoglobin |
| TMTC1 | -1.33154 | 7.96E-61 | 2.58E-58 | -20.0299 | Down | transmembrane O-mannosyltransferase targeting cadherins 1 |
| TOGARAM2 | -1.33092 | 1.63E-16 | 1.49E-15 | -8.65076 | Down | TOG array regulator of axonemal microtubules 2 |
| SDR16C5 | -1.32851 | 1.79E-22 | 2.78E-21 | -10.4291 | Down | short chain dehydrogenase/reductase family 16C member 5 |
| RGR | -1.3272 | 3.63E-16 | 3.22E-15 | -8.54004 | Down | retinal G protein coupled receptor |
| S100A9 | -1.32587 | 2.6E-19 | 3.08E-18 | -9.50952 | Down | S100 calcium binding protein A9 |
| FCGR3A | -1.32476 | 1.14E-35 | 4.89E-34 | -13.9225 | Down | Fc fragment of IgG receptor IIIa |
| KRT7 | -1.3219 | 3.25E-11 | 1.77E-10 | -6.84385 | Down | keratin 7 |
| HS6ST2 | -1.31832 | 5.38E-20 | 6.76E-19 | -9.71245 | Down | heparansulfate 6-O-sulfotransferase 2 |
| SERPINA5 | -1.3169 | 3.35E-31 | 1.06E-29 | -12.7831 | Down | serpin family A member 5 |
| SRARP | -1.31534 | 3.97E-17 | 3.86E-16 | -8.84306 | Down | steroid receptor associated and regulated protein |
| CEP55 | -1.3114 | 5.49E-18 | 5.79E-17 | -9.10864 | Down | centrosomal protein 55 |
| SLC7A11 | -1.30737 | 3.72E-21 | 5.19E-20 | -10.0514 | Down | solute carrier family 7 member 11 |
| ELANE | -1.30624 | 1.4E-12 | 8.75E-12 | -7.33899 | Down | elastase, neutrophil expressed |
| LOC100419170 | -1.30099 | 6.81E-16 | 5.87E-15 | -8.4525 | Down | toll like receptor 2 pseudogene |
| TENT5B | -1.29835 | 4.23E-30 | 1.22E-28 | -12.4969 | Down | terminal nucleotidyltransferase 5B |
| KCNB1 | -1.29075 | 8.19E-36 | 3.55E-34 | -13.9585 | Down | potassium voltage-gated channel subfamily B member 1 |
| GPRC5A | -1.29031 | 7.12E-23 | 1.16E-21 | -10.5421 | Down | G protein-coupled receptor class C group 5 member A |
| FGF5 | -1.28935 | 1.24E-16 | 1.16E-15 | -8.68752 | Down | fibroblast growth factor 5 |
| CFAP77 | -1.28763 | 5.19E-34 | 1.99E-32 | -13.5033 | Down | cilia and flagella associated protein 77 |
| PDE11A | -1.28742 | 1.21E-22 | 1.92E-21 | -10.4774 | Down | phosphodiesterase 11A |
| TMEM178B | -1.28381 | 1.72E-36 | 7.93E-35 | -14.1282 | Down | transmembrane protein 178B |
| MATN3 | -1.28258 | 9.51E-31 | 2.89E-29 | -12.6658 | Down | matrilin 3 |
| SBK3 | -1.28125 | 1.17E-16 | 1.09E-15 | -8.69549 | Down | SH3 domain binding kinase family member 3 |
| GRIP1 | -1.27293 | 3.11E-18 | 3.36E-17 | -9.18402 | Down | glutamate receptor interacting protein 1 |
| PART1 | -1.27148 | 6.39E-23 | 1.05E-21 | -10.5554 | Down | prostate androgen-regulated transcript 1 |
| TIMP4 | -1.26931 | 2.11E-35 | 8.86E-34 | -13.8552 | Down | TIMP metallopeptidase inhibitor 4 |
| EHD4-AS1 | -1.26754 | 9.1E-30 | 2.58E-28 | -12.4099 | Down | EHD4 antisense RNA 1 |
| VSTM2L | -1.26748 | 8.09E-16 | 6.92E-15 | -8.4284 | Down | V-set and transmembrane domain containing 2 like |
| RHAG | -1.26165 | 7.36E-20 | 9.13E-19 | -9.67218 | Down | Rh associated glycoprotein |
| PLIN2 | -1.25946 | 1.41E-53 | 2.89E-51 | -18.2914 | Down | perilipin 2 |
| MYOT | -1.25393 | 2.67E-30 | 7.78E-29 | -12.549 | Down | myotilin |
| LOC100287036 | -1.24828 | 4.78E-16 | 4.2E-15 | -8.50169 | Down | uncharacterized LOC100287036 |
| MEI4 | -1.24458 | 8.2E-20 | 1.01E-18 | -9.65827 | Down | meiotic double-stranded break formation protein 4 |
| NPPC | -1.24339 | 4.79E-13 | 3.16E-12 | -7.50207 | Down | natriuretic peptide C |
| FMO5 | -1.24005 | 7.97E-24 | 1.4E-22 | -10.8086 | Down | flavin containing dimethylanilinemonoxygenase 5 |
| KCNK1 | -1.23892 | 1.6E-40 | 9.84E-39 | -15.1288 | Down | potassium two pore domain channel subfamily K member 1 |
| SYT13 | -1.23357 | 1.52E-22 | 2.38E-21 | -10.4493 | Down | synaptotagmin 13 |
| PRODH | -1.22635 | 1.2E-21 | 1.74E-20 | -10.1925 | Down | proline dehydrogenase 1 |
| FMN1 | -1.22607 | 2.26E-31 | 7.21E-30 | -12.8276 | Down | formin 1 |
| LINC02248 | -1.22281 | 5.24E-10 | 2.49E-09 | -6.38341 | Down | long intergenic non-protein coding RNA 2248 |
| DEPDC1B | -1.22028 | 5.87E-20 | 7.36E-19 | -9.70121 | Down | DEP domain containing 1B |
| DCXR-DT | -1.21729 | 4.05E-22 | 6.09E-21 | -10.3281 | Down | DCXR divergent transcript |
| CPAMD8 | -1.21453 | 4.03E-42 | 2.79E-40 | -15.5212 | Down | C3 and PZP like alpha-2-macroglobulin domain containing 8 |
| FPR1 | -1.21425 | 9.03E-25 | 1.71E-23 | -11.0706 | Down | formyl peptide receptor 1 |
| NQO1 | -1.21175 | 9.69E-37 | 4.54E-35 | -14.1908 | Down | NAD(P)H quinone dehydrogenase 1 |
| MT1X | -1.21115 | 1.52E-17 | 1.54E-16 | -8.97267 | Down | metallothionein 1X |
| GCOM1 | -1.20921 | 2.46E-17 | 2.45E-16 | -8.90776 | Down | GRINL1A complex locus 1 |
| SIGLEC10 | -1.20658 | 4.38E-23 | 7.28E-22 | -10.6014 | Down | sialic acid binding Ig like lectin 10 |
| REELD1 | -1.20551 | 4.1E-20 | 5.2E-19 | -9.74706 | Down | reeler domain containing 1 |
| TMEM63C | -1.20356 | 1.46E-12 | 9.14E-12 | -7.33185 | Down | transmembrane protein 63C |
| CHL1 | -1.19884 | 8.63E-22 | 1.26E-20 | -10.2341 | Down | cell adhesion molecule L1 like |
| FOSL1 | -1.19066 | 2.83E-11 | 1.55E-10 | -6.86606 | Down | FOS like 1, AP-1 transcription factor subunit |
| KIAA1549 | -1.18909 | 1.17E-44 | 9.94E-43 | -16.1398 | Down | KIAA1549 |
| ADAMTS9 | -1.18748 | 1.39E-47 | 1.55E-45 | -16.8488 | Down | ADAM metallopeptidase with thrombospondin type 1 motif 9 |
| F13A1 | -1.17752 | 1.45E-30 | 4.33E-29 | -12.6184 | Down | coagulation factor XIII A chain |
| AASS | -1.177 | 7.57E-23 | 1.23E-21 | -10.5345 | Down | aminoadipate-semialdehyde synthase |
| LUCAT1 | -1.17537 | 2.09E-19 | 2.5E-18 | -9.5374 | Down | lung cancer associated transcript 1 |
| CDH16 | -1.17513 | 1.51E-15 | 1.26E-14 | -8.34112 | Down | cadherin 16 |
| ADAMTS5 | -1.17106 | 2.15E-29 | 5.93E-28 | -12.3122 | Down | ADAM metallopeptidase with thrombospondin type 1 motif 5 |
| KCNIP2 | -1.17026 | 9.89E-23 | 1.59E-21 | -10.5018 | Down | potassium voltage-gated channel interacting protein 2 |
| WWC1 | -1.1615 | 2.31E-15 | 1.9E-14 | -8.28103 | Down | WW and C2 domain containing 1 |
| S100A3 | -1.15808 | 2.16E-13 | 1.48E-12 | -7.62156 | Down | S100 calcium binding protein A3 |
| AGTR1 | -1.15766 | 3.66E-28 | 9.17E-27 | -11.987 | Down | angiotensin II receptor type 1 |
| SERPINF2 | -1.15346 | 1.27E-22 | 2.01E-21 | -10.4714 | Down | serpin family F member 2 |
| PRRG4 | -1.14332 | 5.89E-24 | 1.04E-22 | -10.8453 | Down | proline rich and Gla domain 4 |
| TC2N | -1.14268 | 2.27E-26 | 4.85E-25 | -11.5069 | Down | tandem C2 domains, nuclear |
| TPO | -1.13971 | 1.9E-43 | 1.48E-41 | -15.8455 | Down | thyroid peroxidase |
| GCKR | -1.13956 | 2.58E-13 | 1.75E-12 | -7.59508 | Down | glucokinase regulator |
| GPR82 | -1.13898 | 3.09E-16 | 2.77E-15 | -8.56224 | Down | G protein-coupled receptor 82 |
| CDC45 | -1.13722 | 7.61E-19 | 8.64E-18 | -9.36946 | Down | cell division cycle 45 |
| HERC2P3 | -1.13576 | 2.68E-10 | 1.32E-09 | -6.49664 | Down | hect domain and RLD 2 pseudogene 3 |
| ANGPTL4 | -1.13308 | 3.75E-09 | 1.63E-08 | -6.04163 | Down | angiopoietin like 4 |
| KCNA7 | -1.13228 | 3.56E-12 | 2.15E-11 | -7.1944 | Down | potassium voltage-gated channel subfamily A member 7 |
| ESRP2 | -1.12695 | 3.32E-22 | 5.04E-21 | -10.3527 | Down | epithelial splicing regulatory protein 2 |
| ART3 | -1.12533 | 1.64E-43 | 1.28E-41 | -15.861 | Down | ADP-ribosyltransferase 3 |
| IL20RA | -1.12045 | 3.43E-18 | 3.69E-17 | -9.17123 | Down | interleukin 20 receptor subunit alpha |
| KCNS2 | -1.12023 | 9.08E-15 | 7.05E-14 | -8.08602 | Down | potassium voltage-gated channel modifier subfamily S member 2 |
| MBNL3 | -1.11831 | 3.63E-17 | 3.54E-16 | -8.85536 | Down | muscleblind like splicing regulator 3 |
| GRB14 | -1.11785 | 4.49E-48 | 5.3E-46 | -16.9673 | Down | growth factor receptor bound protein 14 |
| MELK | -1.11646 | 6.76E-13 | 4.38E-12 | -7.44996 | Down | maternal embryonic leucine zipper kinase |
| NAMPT | -1.11563 | 4.32E-36 | 1.92E-34 | -14.0282 | Down | nicotinamidephosphoribosyltransferase |
| TLR2 | -1.11382 | 1.37E-33 | 5.06E-32 | -13.3964 | Down | toll like receptor 2 |
| ADGRF4 | -1.1107 | 7.39E-09 | 3.09E-08 | -5.92035 | Down | adhesion G protein-coupled receptor F4 |
| TTLL6 | -1.10862 | 3.1E-17 | 3.06E-16 | -8.87646 | Down | tubulin tyrosine ligase like 6 |
| KCND3 | -1.10777 | 6.54E-58 | 1.74E-55 | -19.3306 | Down | potassium voltage-gated channel subfamily D member 3 |
| GPSM2 | -1.09924 | 5.94E-32 | 1.98E-30 | -12.9771 | Down | G protein signaling modulator 2 |
| CAMSAP3 | -1.0914 | 2.63E-11 | 1.44E-10 | -6.87822 | Down | calmodulin regulated spectrin associated protein family member 3 |
| NID1 | -1.08953 | 2E-53 | 3.99E-51 | -18.2549 | Down | nidogen 1 |
| MOG | -1.08574 | 8.19E-13 | 5.26E-12 | -7.42074 | Down | myelin oligodendrocyte glycoprotein |
| PALLD | -1.08474 | 2.46E-46 | 2.47E-44 | -16.5468 | Down | palladin, cytoskeletal associated protein |
| MPP3 | -1.08422 | 7.46E-37 | 3.52E-35 | -14.2192 | Down | membrane palmitoylated protein 3 |
| THBS1 | -1.08344 | 2.37E-19 | 2.82E-18 | -9.52148 | Down | thrombospondin 1 |
| SLCO2A1 | -1.0825 | 3.32E-35 | 1.39E-33 | -13.8055 | Down | solute carrier organic anion transporter family member 2A1 |
| SPOCK3 | -1.07804 | 2.78E-08 | 1.09E-07 | -5.67797 | Down | SPARC (osteonectin), cwcv and kazal like domains proteoglycan 3 |
| CR1 | -1.07745 | 6.04E-16 | 5.23E-15 | -8.46919 | Down | complement C3b/C4b receptor 1 (Knops blood group) |
| TUBB4A | -1.07702 | 7.72E-19 | 8.77E-18 | -9.36749 | Down | tubulin beta 4A class IVa |
| F8 | -1.07543 | 1.03E-33 | 3.85E-32 | -13.4275 | Down | coagulation factor VIII |
| ZNF366 | -1.07245 | 1.55E-09 | 6.99E-09 | -6.19713 | Down | zinc finger protein 366 |
| ADGRD1 | -1.07197 | 3.68E-56 | 8.89E-54 | -18.9109 | Down | adhesion G protein-coupled receptor D1 |
| LINC01844 | -1.06936 | 1.81E-20 | 2.35E-19 | -9.85152 | Down | long intergenic non-protein coding RNA 1844 |
| SLA | -1.06836 | 1.06E-25 | 2.14E-24 | -11.3249 | Down | Src like adaptor |
| DNER | -1.06766 | 5.57E-16 | 4.85E-15 | -8.48051 | Down | delta/notch like EGF repeat containing |
| C20orf197 | -1.06693 | 7.83E-15 | 6.13E-14 | -8.1072 | Down | chromosome 20 open reading frame 197 |
| FAM83F | -1.06644 | 1.85E-29 | 5.14E-28 | -12.3289 | Down | family with sequence similarity 83 member F |
| SSTR5-AS1 | -1.06089 | 8.95E-06 | 2.52E-05 | -4.50452 | Down | SSTR5 antisense RNA 1 |
| VIT | -1.0607 | 1.39E-32 | 4.82E-31 | -13.139 | Down | vitrin |
| LRRC8E | -1.06061 | 2.76E-19 | 3.26E-18 | -9.50137 | Down | leucine rich repeat containing 8 VRAC subunit E |
| STOX1 | -1.05648 | 1.79E-20 | 2.33E-19 | -9.85271 | Down | storkhead box 1 |
| PKHD1L1 | -1.05526 | 2.52E-10 | 1.24E-09 | -6.50688 | Down | PKHD1 like 1 |
| ALOX5 | -1.05509 | 5.75E-39 | 3.14E-37 | -14.7448 | Down | arachidonate 5-lipoxygenase |
| SMTNL2 | -1.05192 | 4.46E-31 | 1.39E-29 | -12.7511 | Down | smoothelin like 2 |
| SPC24 | -1.05123 | 1.24E-28 | 3.22E-27 | -12.1119 | Down | SPC24 component of NDC80 kinetochore complex |
| OSMR | -1.04998 | 3.8E-35 | 1.58E-33 | -13.7905 | Down | oncostatin M receptor |
| PPM1E | -1.04982 | 1.48E-07 | 5.31E-07 | -5.35894 | Down | protein phosphatase, Mg2+/Mn2+ dependent 1E |
| SAMSN1 | -1.04852 | 2.33E-19 | 2.78E-18 | -9.52335 | Down | SAM domain, SH3 domain and nuclear localization signals 1 |
| CD109 | -1.04808 | 1.53E-42 | 1.1E-40 | -15.6243 | Down | CD109 molecule |
| CBS | -1.04794 | 1.09E-28 | 2.84E-27 | -12.1267 | Down | cystathionine beta-synthase |
| GPR12 | -1.04772 | 2E-14 | 1.51E-13 | -7.97205 | Down | G protein-coupled receptor 12 |
| SYN3 | -1.04745 | 2.11E-24 | 3.86E-23 | -10.969 | Down | synapsin III |
| CCR1 | -1.04606 | 5.34E-27 | 1.2E-25 | -11.6762 | Down | C-C motif chemokine receptor 1 |
| GRB7 | -1.04361 | 2.79E-19 | 3.29E-18 | -9.50014 | Down | growth factor receptor bound protein 7 |
| PPL | -1.04306 | 6.42E-43 | 4.83E-41 | -15.7162 | Down | periplakin |
| LOC100507516 | -1.04141 | 1.94E-20 | 2.52E-19 | -9.84253 | Down | uncharacterized LOC100507516 |
| GPR183 | -1.04045 | 2.19E-20 | 2.83E-19 | -9.827 | Down | G protein-coupled receptor 183 |
| TLCD2 | -1.04031 | 3.05E-23 | 5.12E-22 | -10.6458 | Down | TLC domain containing 2 |
| PTX3 | -1.0381 | 9.31E-10 | 4.3E-09 | -6.28492 | Down | pentraxin 3 |
| KLHL32 | -1.03311 | 1.69E-18 | 1.87E-17 | -9.26454 | Down | kelch like family member 32 |
| ZDHHC20 | -1.03268 | 6.12E-07 | 2.03E-06 | -5.07664 | Down | zinc finger DHHC-type containing 20 |
| CNR1 | -1.03125 | 3.36E-14 | 2.48E-13 | -7.89646 | Down | cannabinoid receptor 1 |
| RPGR | -1.03109 | 2.64E-38 | 1.38E-36 | -14.5809 | Down | retinitis pigmentosaGTPase regulator |
| TFEC | -1.02784 | 2.48E-18 | 2.7E-17 | -9.21407 | Down | transcription factor EC |
| MSR1 | -1.02631 | 2.77E-23 | 4.69E-22 | -10.6573 | Down | macrophage scavenger receptor 1 |
| CDRT1 | -1.02519 | 1.15E-19 | 1.4E-18 | -9.6148 | Down | CMT1A duplicated region transcript 1 |
| FREM2 | -1.02482 | 1.96E-09 | 8.73E-09 | -6.15591 | Down | FRAS1 related extracellular matrix 2 |
| F5 | -1.02393 | 6.87E-10 | 3.22E-09 | -6.3371 | Down | coagulation factor V |
| SMIM9 | -1.01786 | 2.38E-19 | 2.83E-18 | -9.52073 | Down | small integral membrane protein 9 |
| NECTIN1 | -1.01749 | 6.29E-46 | 5.97E-44 | -16.4481 | Down | nectin cell adhesion molecule 1 |
| ZFP57 | -1.01535 | 0.0101 | 0.017165 | -2.5858 | Down | ZFP57 zinc finger protein |
| LINC02289 | -1.01329 | 1.62E-28 | 4.16E-27 | -12.0808 | Down | long intergenic non-protein coding RNA 2289 |
| CDKL5 | -1.01252 | 9.24E-06 | 2.6E-05 | -4.49752 | Down | cyclin dependent kinase like 5 |
| EIF4EBP1 | -1.01195 | 8.78E-18 | 9.12E-17 | -9.04606 | Down | eukaryotic translation initiation factor 4E binding protein 1 |
| LOC100129434 | -1.0107 | 1.39E-21 | 2.01E-20 | -10.1743 | Down | uncharacterized LOC100129434 |
| ITPKA | -1.01004 | 8.87E-11 | 4.6E-10 | -6.68031 | Down | inositol-trisphosphate 3-kinase A |
| HAS2 | -1.00952 | 1.03E-17 | 1.07E-16 | -9.02455 | Down | hyaluronan synthase 2 |
| PCNT | -1.00784 | 4.48E-52 | 7.63E-50 | -17.9305 | Down | pericentrin |
| NUDT4 | -1.00756 | 4.52E-36 | 2E-34 | -14.0233 | Down | nudix hydrolase 4 |
| ELF3 | -1.00732 | 1.12E-10 | 5.76E-10 | -6.6416 | Down | E74 like ETS transcription factor 3 |
| B3GALT1 | -1.00415 | 1.6E-17 | 1.62E-16 | -8.96561 | Down | beta-1,3-galactosyltransferase 1 |
| SIGLEC14 | -1.0039 | 5.59E-10 | 2.65E-09 | -6.37216 | Down | sialic acid binding Ig like lectin 14 |
| NUDT4B | -0.99985 | 5.54E-10 | 2.63E-09 | -6.37373 | Down | nudix hydrolase 4B |
| CLEC7A | -0.99973 | 9.85E-23 | 1.58E-21 | -10.5023 | Down | C-type lectin domain containing 7A |
| RNF157 | -0.99959 | 1.36E-31 | 4.4E-30 | -12.8846 | Down | ring finger protein 157 |
| STEAP4 | -0.99897 | 2.2E-31 | 7.03E-30 | -12.8307 | Down | STEAP4 metalloreductase |
| KCNK3 | -0.99891 | 2.34E-20 | 3.03E-19 | -9.8184 | Down | potassium two pore domain channel subfamily K member 3 |
| SAMHD1 | -0.99873 | 6.11E-53 | 1.15E-50 | -18.1382 | Down | SAM and HD domain containing deoxynucleoside triphosphate triphosphohydrolase 1 |
| INSYN1 | -0.99843 | 1.37E-27 | 3.22E-26 | -11.8347 | Down | inhibitory synaptic factor 1 |
| INKA2 | -0.99831 | 6.14E-61 | 2.06E-58 | -20.057 | Down | inka box actin regulator 2 |
| BCAT1 | -0.99623 | 1.02E-28 | 2.68E-27 | -12.1336 | Down | branched chain amino acid transaminase 1 |
| SLC5A1 | -0.99614 | 1.53E-30 | 4.56E-29 | -12.6121 | Down | solute carrier family 5 member 1 |
| SLC38A4 | -0.99485 | 9.56E-11 | 4.94E-10 | -6.6679 | Down | solute carrier family 38 member 4 |
| CSDC2 | -0.99426 | 2.67E-21 | 3.77E-20 | -10.0929 | Down | cold shock domain containing C2 |
| ETNK2 | -0.99114 | 1.11E-30 | 3.37E-29 | -12.6479 | Down | ethanolamine kinase 2 |
| PHACTR3 | -0.98446 | 3.47E-12 | 2.1E-11 | -7.19855 | Down | phosphatase and actin regulator 3 |
| INKA2-AS1 | -0.98382 | 1.85E-30 | 5.48E-29 | -12.5904 | Down | INKA2 antisense RNA 1 |
| LGR5 | -0.98318 | 2.63E-11 | 1.45E-10 | -6.8778 | Down | leucine rich repeat containing G protein-coupled receptor 5 |
| LGR6 | -0.98193 | 2.9E-23 | 4.89E-22 | -10.6518 | Down | leucine rich repeat containing G protein-coupled receptor 6 |
| ADGRF5 | -0.98027 | 7.74E-38 | 3.89E-36 | -14.4646 | Down | adhesion G protein-coupled receptor F5 |
| LOC105370401 | -0.9795 | 2.07E-07 | 7.27E-07 | -5.29359 | Down | uncharacterized LOC105370401 |
| ADAMTS15 | -0.97867 | 3.08E-41 | 1.99E-39 | -15.3047 | Down | ADAM metallopeptidase with thrombospondin type 1 motif 15 |
| C1QTNF1 | -0.97823 | 6.59E-19 | 7.53E-18 | -9.38821 | Down | C1q and TNF related 1 |
| SIGLEC7 | -0.97434 | 1.41E-21 | 2.03E-20 | -10.1729 | Down | sialic acid binding Ig like lectin 7 |
| TRHDE | -0.97406 | 2.27E-09 | 1E-08 | -6.13046 | Down | thyrotropin releasing hormone degrading enzyme |
| LILRA6 | -0.97359 | 2.41E-21 | 3.41E-20 | -10.106 | Down | leukocyte immunoglobulin like receptor A6 |
| SERPINB8 | -0.97086 | 2.03E-34 | 8.05E-33 | -13.6069 | Down | serpin family B member 8 |
| LRRN3 | -0.97031 | 2.16E-14 | 1.62E-13 | -7.96079 | Down | leucine rich repeat neuronal 3 |
| MT1M | -0.96838 | 6.61E-09 | 2.79E-08 | -5.94048 | Down | metallothionein 1M |
| WDR86-AS1 | -0.9683 | 3.6E-10 | 1.74E-09 | -6.44704 | Down | WDR86 antisense RNA 1 |
| PCK1 | -0.96425 | 0.00069 | 0.001457 | -3.42273 | Down | phosphoenolpyruvatecarboxykinase 1 |
| EDN1 | -0.96378 | 1.44E-25 | 2.87E-24 | -11.289 | Down | endothelin 1 |
| KLF10 | -0.96307 | 1.82E-29 | 5.05E-28 | -12.3313 | Down | Kruppel like factor 10 |
| MANEA | -0.96278 | 5.36E-17 | 5.16E-16 | -8.80226 | Down | mannosidaseendo-alpha |
| TUBAL3 | -0.96258 | 6.11E-13 | 3.98E-12 | -7.46512 | Down | tubulin alpha like 3 |
| PMFBP1 | -0.96247 | 6.09E-12 | 3.59E-11 | -7.11034 | Down | polyamine modulated factor 1 binding protein 1 |
| DAAM2-AS1 | -0.96155 | 1.13E-30 | 3.41E-29 | -12.6465 | Down | DAAM2 antisense RNA 1 |
| ABCB1 | -0.96049 | 1.45E-23 | 2.49E-22 | -10.7363 | Down | ATP binding cassette subfamily B member 1 |
| ELL2 | -0.95986 | 1.16E-29 | 3.25E-28 | -12.3828 | Down | elongation factor for RNA polymerase II 2 |
| HIF3A | -0.95866 | 2.09E-33 | 7.63E-32 | -13.3492 | Down | hypoxia inducible factor 3 subunit alpha |
| ZBED6 | -0.95784 | 0.000413 | 0.000904 | -3.56438 | Down | zinc finger BED-type containing 6 |
| GFI1B | -0.95657 | 2.87E-12 | 1.75E-11 | -7.22798 | Down | growth factor independent 1B transcriptional repressor |
| HMOX2 | -0.95588 | 9.44E-45 | 8.14E-43 | -16.1626 | Down | hemeoxygenase 2 |
| TK1 | -0.95442 | 7.38E-13 | 4.77E-12 | -7.43647 | Down | thymidine kinase 1 |
| SLC36A4 | -0.95143 | 6.25E-49 | 8.02E-47 | -17.1738 | Down | solute carrier family 36 member 4 |
| TMEM132C | -0.95116 | 3.19E-18 | 3.45E-17 | -9.18058 | Down | transmembrane protein 132C |
| STEAP3 | -0.95098 | 3.34E-49 | 4.37E-47 | -17.2393 | Down | STEAP3 metalloreductase |
| PCLAF | -0.9505 | 3.17E-10 | 1.54E-09 | -6.46829 | Down | PCNA clamp associated factor |
| ALOX5AP | -0.94867 | 2.9E-25 | 5.66E-24 | -11.2059 | Down | arachidonate 5-lipoxygenase activating protein |
| SLC1A1 | -0.94735 | 1.64E-32 | 5.67E-31 | -13.1204 | Down | solute carrier family 1 member 1 |
| EHF | -0.94691 | 3.32E-10 | 1.62E-09 | -6.4604 | Down | ETS homologous factor |
| CCDC68 | -0.94642 | 4.3E-18 | 4.58E-17 | -9.14123 | Down | coiled-coil domain containing 68 |
| BCL6 | -0.94633 | 5.81E-61 | 2.01E-58 | -20.0628 | Down | BCL6 transcription repressor |
| SLC31A2 | -0.94625 | 2.68E-53 | 5.31E-51 | -18.224 | Down | solute carrier family 31 member 2 |
| TXNRD1 | -0.94184 | 6.66E-33 | 2.35E-31 | -13.2209 | Down | thioredoxinreductase 1 |
| GGT5 | -0.93863 | 5.73E-17 | 5.5E-16 | -8.79326 | Down | gamma-glutamyltransferase 5 |
| GRAP2 | -0.93849 | 7.28E-23 | 1.19E-21 | -10.5394 | Down | GRB2 related adaptor protein 2 |
| IGSF22 | -0.9363 | 5.2E-23 | 8.57E-22 | -10.5805 | Down | immunoglobulin superfamily member 22 |
| MARVELD2 | -0.93616 | 4.75E-29 | 1.28E-27 | -12.2216 | Down | MARVEL domain containing 2 |
| SLC19A2 | -0.93586 | 3.58E-19 | 4.2E-18 | -9.46764 | Down | solute carrier family 19 member 2 |
| SELE | -0.93559 | 3.88E-06 | 1.15E-05 | -4.68889 | Down | selectin E |
| LINC01235 | -0.93519 | 2.15E-08 | 8.55E-08 | -5.7253 | Down | long intergenic non-protein coding RNA 1235 |
| RAB39A | -0.93499 | 3E-11 | 1.64E-10 | -6.85672 | Down | RAB39A, member RAS oncogene family |
| ANLN | -0.93394 | 6.42E-10 | 3.02E-09 | -6.3487 | Down | anillin actin binding protein |
| CLSPN | -0.93196 | 9.75E-13 | 6.21E-12 | -7.39409 | Down | claspin |
| ACPP | -0.93112 | 4.27E-15 | 3.43E-14 | -8.19383 | Down | acid phosphatase, prostate |
| LRGUK | -0.92812 | 1.91E-12 | 1.18E-11 | -7.29067 | Down | leucine rich repeats and guanylate kinase domain containing |
| ARG1 | -0.9274 | 1.06E-06 | 3.42E-06 | -4.96388 | Down | arginase 1 |
| P2RY12 | -0.92637 | 1.78E-10 | 8.9E-10 | -6.56544 | Down | purinergic receptor P2Y12 |
| ARNTL | -0.92612 | 7.2E-18 | 7.53E-17 | -9.07262 | Down | aryl hydrocarbon receptor nuclear translocator like |
| LINC01684 | -0.92272 | 1.34E-13 | 9.32E-13 | -7.69327 | Down | long intergenic non-protein coding RNA 1684 |
| C1orf162 | -0.92206 | 1.85E-35 | 7.8E-34 | -13.8696 | Down | chromosome 1 open reading frame 162 |
| TRPC4 | -0.92014 | 2.7E-18 | 2.94E-17 | -9.20265 | Down | transient receptor potential cation channel subfamily C member 4 |
| AIF1L | -0.91594 | 1.75E-52 | 3.13E-50 | -18.0287 | Down | allograft inflammatory factor 1 like |
| KIF18B | -0.91286 | 4.17E-10 | 2.01E-09 | -6.42185 | Down | kinesin family member 18B |
| CRISPLD2 | -0.9128 | 1.26E-38 | 6.7E-37 | -14.66 | Down | cysteine rich secretory protein LCCL domain containing 2 |
| AMD1 | -0.91169 | 1.46E-48 | 1.79E-46 | -17.0851 | Down | adenosylmethionine decarboxylase 1 |
| FLT3 | -0.91165 | 2.93E-14 | 2.17E-13 | -7.91632 | Down | fms related tyrosine kinase 3 |
| ARRDC4 | -0.91072 | 3.74E-26 | 7.89E-25 | -11.4482 | Down | arrestin domain containing 4 |
| SLC4A7 | -0.91068 | 3.18E-13 | 2.13E-12 | -7.56366 | Down | solute carrier family 4 member 7 |
| TOP2A | -0.9091 | 2.87E-09 | 1.26E-08 | -6.08903 | Down | DNA topoisomerase II alpha |
| CENPM | -0.90902 | 4.38E-08 | 1.68E-07 | -5.59272 | Down | centromere protein M |
| S100A8 | -0.90893 | 7.54E-10 | 3.51E-09 | -6.32114 | Down | S100 calcium binding protein A8 |
| SIRPB2 | -0.90766 | 3.38E-19 | 3.98E-18 | -9.47503 | Down | signal regulatory protein beta 2 |
| MEDAG | -0.90694 | 8.83E-23 | 1.43E-21 | -10.5157 | Down | mesenteric estrogen dependent adipogenesis |
| SLC2A1 | -0.90689 | 9.89E-45 | 8.49E-43 | -16.1578 | Down | solute carrier family 2 member 1 |
| SLC52A3 | -0.90653 | 5.43E-32 | 1.82E-30 | -12.9872 | Down | solute carrier family 52 member 3 |
| SOCS3 | -0.90502 | 2.05E-10 | 1.02E-09 | -6.54144 | Down | suppressor of cytokine signaling 3 |
| PDK4 | -0.90267 | 1.31E-10 | 6.69E-10 | -6.61548 | Down | pyruvate dehydrogenase kinase 4 |
| GLUL | -0.90223 | 8.16E-46 | 7.67E-44 | -16.4208 | Down | glutamate-ammonia ligase |
| HPSE | -0.90161 | 2.07E-23 | 3.53E-22 | -10.6928 | Down | heparanase |
| GABRR2 | -0.90116 | 2.86E-06 | 8.66E-06 | -4.75471 | Down | gamma-aminobutyric acid type A receptor rho2 subunit |
| FAM111B | -0.90086 | 1.25E-08 | 5.12E-08 | -5.82455 | Down | family with sequence similarity 111 member B |
| SNORD101 | -0.90003 | 2.16E-11 | 1.19E-10 | -6.90987 | Down | small nucleolar RNA, C/D box 101 |
| WASF1 | -0.89554 | 6.06E-27 | 1.36E-25 | -11.6614 | Down | WASP family member 1 |
| KIF5C | -0.89536 | 4.84E-25 | 9.31E-24 | -11.145 | Down | kinesin family member 5C |
| MARK3 | -0.89489 | 6.12E-82 | 1.27E-78 | -25.1753 | Down | microtubule affinity regulating kinase 3 |
| SLC30A10 | -0.89439 | 1.02E-12 | 6.49E-12 | -7.38702 | Down | solute carrier family 30 member 10 |
| TGFBR3 | -0.89378 | 5.11E-39 | 2.79E-37 | -14.7576 | Down | transforming growth factor beta receptor 3 |
| FCER1G | -0.89232 | 1.4E-26 | 3.05E-25 | -11.5633 | Down | Fc fragment of IgE receptor Ig |
| KLHL41 | -0.89223 | 5.91E-33 | 2.09E-31 | -13.2341 | Down | kelch like family member 41 |
| EDN2 | -0.89167 | 6.37E-09 | 2.69E-08 | -5.94703 | Down | endothelin 2 |
| CHI3L1 | -0.89162 | 3.32E-05 | 8.66E-05 | -4.20254 | Down | chitinase 3 like 1 |
| SFRP5 | -0.89029 | 1.32E-07 | 4.77E-07 | -5.38124 | Down | secreted frizzled related protein 5 |
| LAPTM5 | -0.88976 | 1.02E-22 | 1.64E-21 | -10.4975 | Down | lysosomal protein transmembrane 5 |
| SLC9A7 | -0.88906 | 6.45E-22 | 9.53E-21 | -10.2702 | Down | solute carrier family 9 member A7 |
| C8orf88 | -0.88787 | 7.65E-51 | 1.14E-48 | -17.6342 | Down | chromosome 8 open reading frame 88 |
| CLEC4G | -0.88685 | 7.21E-07 | 2.37E-06 | -5.04324 | Down | C-type lectin domain family 4 member G |
| GPAT2 | -0.88402 | 1.9E-17 | 1.91E-16 | -8.94253 | Down | glycerol-3-phosphate acyltransferase 2, mitochondrial |
| SCN3A | -0.88292 | 8.31E-14 | 5.91E-13 | -7.76375 | Down | sodium voltage-gated channel alpha subunit 3 |
| BANK1 | -0.88095 | 1E-10 | 5.18E-10 | -6.66001 | Down | B cell scaffold protein with ankyrin repeats 1 |
| SEC14L2 | -0.88054 | 1.42E-15 | 1.19E-14 | -8.34963 | Down | SEC14 like lipid binding 2 |
| LANCL3 | -0.87923 | 1.15E-10 | 5.92E-10 | -6.63674 | Down | LanC like 3 |
| E2F2 | -0.87786 | 2.5E-09 | 1.1E-08 | -6.11349 | Down | E2F transcription factor 2 |
| IL15RA | -0.87627 | 2.9E-47 | 3.17E-45 | -16.7715 | Down | interleukin 15 receptor subunit alpha |
| ACR | -0.87563 | 6.53E-15 | 5.15E-14 | -8.13328 | Down | acrosin |
| ATP2A2 | -0.8755 | 1.39E-30 | 4.18E-29 | -12.6227 | Down | ATPase sarcoplasmic/endoplasmic reticulum Ca2+ transporting 2 |
| MAMDC2 | -0.87509 | 9.15E-18 | 9.49E-17 | -9.04054 | Down | MAM domain containing 2 |
| MYZAP | -0.87372 | 3.64E-05 | 9.46E-05 | -4.18075 | Down | myocardial zonulaadherens protein |
| EBLN2 | -0.87327 | 3.93E-06 | 1.17E-05 | -4.68593 | Down | endogenous Bornavirus like nucleoprotein 2 |
| USP31 | -0.87252 | 1.22E-40 | 7.61E-39 | -15.1575 | Down | ubiquitin specific peptidase 31 |
| LRMDA | -0.87106 | 1.27E-21 | 1.83E-20 | -10.1859 | Down | leucine rich melanocyte differentiation associated |
| GPR4 | -0.87078 | 8.84E-33 | 3.09E-31 | -13.1895 | Down | G protein-coupled receptor 4 |
| OPN4 | -0.87003 | 1.81E-11 | 1.01E-10 | -6.9378 | Down | opsin 4 |
| PPIP5K2 | -0.86979 | 1.84E-24 | 3.38E-23 | -10.9854 | Down | diphosphoinositolpentakisphosphate kinase 2 |
| SNAI3 | -0.86358 | 4.07E-11 | 2.19E-10 | -6.8073 | Down | snail family transcriptional repressor 3 |
| CDCP1 | -0.86318 | 2.13E-16 | 1.94E-15 | -8.6134 | Down | CUB domain containing protein 1 |
| PAPSS2 | -0.86311 | 1.62E-26 | 3.51E-25 | -11.5461 | Down | 3'-phosphoadenosine 5'-phosphosulfate synthase 2 |
| KIF14 | -0.86236 | 6.12E-09 | 2.59E-08 | -5.95432 | Down | kinesin family member 14 |
| LNCOG | -0.86037 | 2.38E-07 | 8.3E-07 | -5.2659 | Down | lncRNAosteogenesis associated |
| FAM107A | -0.85818 | 2.59E-40 | 1.57E-38 | -15.0771 | Down | family with sequence similarity 107 member A |
| ANKRD7 | -0.85782 | 2.72E-14 | 2.02E-13 | -7.92723 | Down | ankyrin repeat domain 7 |
| AKR1C1 | -0.85714 | 6.87E-23 | 1.12E-21 | -10.5464 | Down | aldo-ketoreductase family 1 member C1 |
| LINC02177 | -0.85669 | 7.6E-08 | 2.83E-07 | -5.48796 | Down | long intergenic non-protein coding RNA 2177 |
| LSAMP | -0.85556 | 9.78E-14 | 6.9E-13 | -7.73966 | Down | limbic system associated membrane protein |
| DOCK9 | -0.8547 | 6.23E-22 | 9.22E-21 | -10.2746 | Down | dedicator of cytokinesis 9 |
| MS4A4A | -0.85425 | 1.02E-21 | 1.48E-20 | -10.2137 | Down | membrane spanning 4-domains A4A |
| LINC01128 | -0.85327 | 5.03E-32 | 1.69E-30 | -12.9958 | Down | long intergenic non-protein coding RNA 1128 |
| HK3 | -0.85159 | 2.75E-16 | 2.48E-15 | -8.57828 | Down | hexokinase 3 |
| SNTG2 | -0.84977 | 2.78E-16 | 2.5E-15 | -8.57682 | Down | syntrophin gamma 2 |
| EPB41L4B | -0.84874 | 8.53E-24 | 1.49E-22 | -10.8005 | Down | erythrocyte membrane protein band 4.1 like 4B |
| LOC105378721 | -0.84723 | 1.25E-26 | 2.73E-25 | -11.577 | Down | uncharacterized LOC105378721 |
| CALCRL | -0.84699 | 2.1E-13 | 1.43E-12 | -7.62605 | Down | calcitonin receptor like receptor |
| CTH | -0.84525 | 2.34E-27 | 5.43E-26 | -11.7723 | Down | cystathionine gamma-lyase |
| ANXA3 | -0.84508 | 2.77E-39 | 1.55E-37 | -14.8233 | Down | annexin A3 |
| NCEH1 | -0.84422 | 1.43E-27 | 3.35E-26 | -11.8296 | Down | neutral cholesterol ester hydrolase 1 |
| RASD1 | -0.84259 | 3.42E-06 | 1.03E-05 | -4.71643 | Down | ras related dexamethasone induced 1 |
| MERTK | -0.84249 | 1.24E-28 | 3.23E-27 | -12.1114 | Down | MER proto-oncogene, tyrosine kinase |
| TCHH | -0.84214 | 3.38E-11 | 1.83E-10 | -6.83748 | Down | trichohyalin |
| APOB | -0.84165 | 1.21E-17 | 1.24E-16 | -9.00323 | Down | apolipoprotein B |
| EPN3 | -0.84144 | 2.21E-12 | 1.36E-11 | -7.26849 | Down | epsin 3 |
| LOC101927858 | -0.84129 | 3.29E-13 | 2.2E-12 | -7.55892 | Down | uncharacterized LOC101927858 |
| ECT2 | -0.83943 | 2.39E-17 | 2.39E-16 | -8.91154 | Down | epithelial cell transforming 2 |
| TMEM100 | -0.83941 | 2.92E-15 | 2.38E-14 | -8.24789 | Down | transmembrane protein 100 |
| MYBL2 | -0.83919 | 1.2E-07 | 4.34E-07 | -5.40031 | Down | MYB proto-oncogene like 2 |
| AFF2 | -0.83824 | 1.98E-10 | 9.87E-10 | -6.54735 | Down | AF4/FMR2 family member 2 |
| VEPH1 | -0.83738 | 2.57E-11 | 1.41E-10 | -6.8819 | Down | ventricular zone expressed PH domain containing 1 |
| RAET1E | -0.83675 | 8.68E-17 | 8.2E-16 | -8.73659 | Down | retinoic acid early transcript 1E |
| IRAK3 | -0.8363 | 1.24E-19 | 1.51E-18 | -9.60519 | Down | interleukin 1 receptor associated kinase 3 |
| ARMC12 | -0.83589 | 1.42E-15 | 1.19E-14 | -8.34925 | Down | armadillo repeat containing 12 |
| HPR | -0.83392 | 6.8E-11 | 3.58E-10 | -6.72376 | Down | haptoglobin-related protein |
| MT1JP | -0.83257 | 1.41E-06 | 4.47E-06 | -4.9042 | Down | metallothionein 1J, pseudogene |
| PIK3C2A | -0.83111 | 7.57E-15 | 5.93E-14 | -8.11208 | Down | phosphatidylinositol-4-phosphate 3-kinase catalytic subunit type 2 alpha |
| FGR | -0.8308 | 1.35E-26 | 2.94E-25 | -11.5676 | Down | FGR proto-oncogene, Src family tyrosine kinase |
| NHLRC2 | -0.83012 | 1.09E-13 | 7.65E-13 | -7.72382 | Down | NHL repeat containing 2 |
